# The Ugi4CR as effective tool to access promising anticancer isatin-based α-acetamide carboxamide oxindole hybrids

**DOI:** 10.3762/bjoc.20.104

**Published:** 2024-05-27

**Authors:** Carolina S Marques, Aday González-Bakker, José M Padrón

**Affiliations:** 1 LAQV-REQUIMTE, University of Évora, Institute for Research and Advanced Studies, Rua Romão Ramalho, 59, 7000-641, Évora, Portugal; 2 BioLab, Instituto Universitario de Bio-Orgánica Antonio González (IUBO-AG), Universidad de La Laguna, PO Box 456, 38200, La Laguna, Spain

**Keywords:** cancer, GI_50_, isatin, oxindole, Ugi4CR

## Abstract

Considering early-stage drug discovery programs, the Ugi four-component reaction is a valuable, flexible, and pivotal tool, facilitating the creation of two new amide bonds in a one-pot fashion to effectively yield the desired α-aminoacylamides. Here, we highlight the reputation of this reaction approach to access number and scaffold diversity of a library of isatin-based α-acetamide carboxamide oxindole hybrids, promising anticancer agents, in a mild and fast sustainable reaction process. The library was tested against six human solid tumor cell lines, among them, non-small cell lung carcinoma, cervical adenocarcinoma, breast cancer and colon adenocarcinoma. The most potent compounds **8d**, **8h** and **8k** showed GI_50_ values in the range of 1–10 μM.

## Introduction

**Figure 1:**
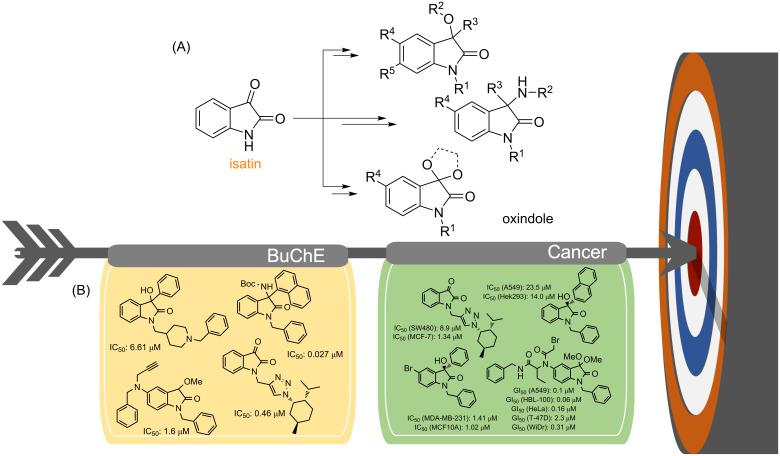
(A) Accessing libraries of oxindole hybrids using commercially available isatin as starting material and (B) most potent oxindole derivatives as BuChE inhibitors and anticancer agents, by Marques et al. [10–16].

Meticulous attention has been given by chemists regarding process formation of new bonds and synthesis of new scaffolds. In drug discovery and development, medicinal chemists struggle everyday towards the creation of new synthetic methods, driven by the increasing complexity of the molecules and taking into consideration economic and social aspects. Multicomponent reactions (MCRs) are remarkable tools which demonstrated great potential for more sustainable production of active pharmaceutical ingredients (API’s). These flexible and versatile one-pot transformations in which three or more reagents are combined to access a new complex scaffold with remarkable atom economy, cost and time-effective and mainly diminishing waste production is a conscientious boost for structural diversity and sustainability [1–3]. The well-known Passerini, Ugi, Mannich, Biginelli, Hantzsch and Strecker reactions are some examples of the classic MCRs, representing the easygoing generation of a collection of small-molecules essential for structure–activity relationships (SAR). The isocyanide-based Ugi reaction is one of the most resourceful tools and still broadly studied MCR, generating multifunctional libraries of α-aminoacylamide derivatives, or Ugi adducts, with stereochemistry control [4–5]. Unquestionable potential of application in the pharmaceutical industry is recognizable by the number of APIs obtained by this reaction approach [6–7]. The oxindole framework is a privileged unit, recognized massively by its extensive biological applications [8–9]. In the last few years we have been active in isatin modification using new synthetic approaches, anticipating the creation of new libraries of small-molecule hybrids with potential as cholinesterase inhibitors [10–13], important to treat neurodegenerative diseases, and anticancer agents [14–16] (Figure 1).

Cancer is a complex, cureless and fatal disease, oftentimes diagnosed worldwide. Being one of the leading causes of death worldwide, it is expected an increase of 47% with 28.4 million cases diagnosed, in 2040 [17–18]. Despite long years of research, there is still an urgent need to find novel, effective and safe drugs for cancer therapy.

**Scheme 1:**
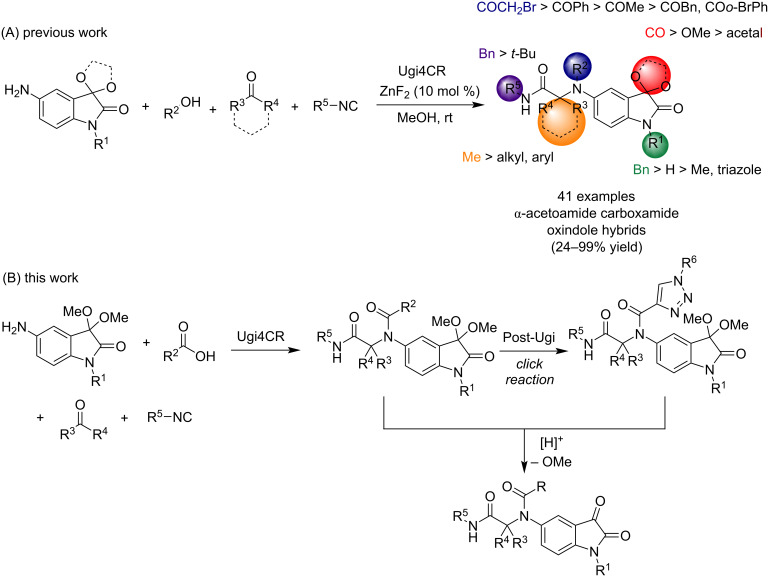
(A) Library of isatin-based α-acetamide carboxamide oxindole derivatives obtained using an Ugi four-component reaction (Ugi4CR). (B) The synthetic strategy reported in this work.

Recently, focusing on the design of more potent anticancer drug candidates using more sustainable synthetic processes, we report a new Ugi four-component reaction approach for easy access to Ugi-derived isatin-peptoids in moderate to excellent yields (up to 99% yield). Some selected compounds were screened against five human solid tumor cell lines: lung (A549), breast (HBL-100 and T-47D), cervix (HeLa) and colon (WiDr). Preliminary SAR studies have revealed the preference of the *N*-benzylisatin structure over the 3,3-protected-oxindole, aliphatic chain on the acid component and small aliphatic chain on the aldehyde component to increase the antiproliferative activity. Also, benzyl isocyanide was favored over the aliphatic one (Scheme 1A) [16]. Considering the value of amide groups in drug discovery [19], the feasibility of running the isatin-based Ugi reaction [16,20–23] and the potential of the bis-amide-oxindole type derivatives as anticancer agents, a second family was synthesized, and screened for their anticancer activities (Scheme 1B).

## Results and Discussion

### Synthesis

**Scheme 2:**
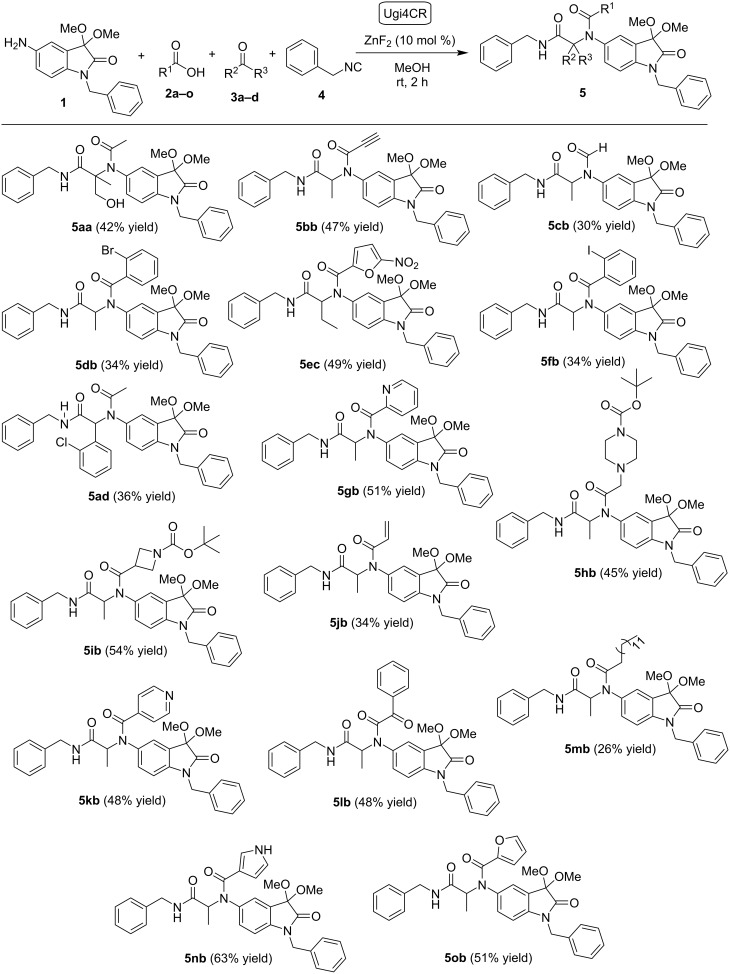
Library of α-acetamide carboxamide oxindole hybrids **5** accessed via the Ugi4CR.

**Figure 2:**
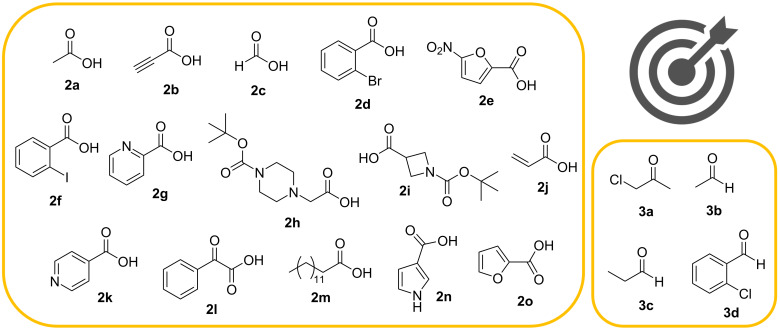
Carboxylic acids **2** and aldehydes/ketones **3** used in the Ugi4CR.

Underlining sustainability and economically favored processes, a second family of α-acetamide carboxamide oxindole derivatives **5** was obtained using the previously optimized Ugi4CR approach [16] (Scheme 2 and Figure 2). Taking into account the preliminary SAR studies reported for the first family of Ugi-derived isatin-peptoids, the second family was obtained using 5-amino-1-benzyl-3,3-dimethoxyindolin-2-one (**1**) [12] and benzyl isocyanide (**4**), as amine and isocyanide components, respectively. Different carboxylic acids **2** and aldehydes/ketones **3** were evaluated using ZnF_2_ as catalyst (10 mol %) and MeOH as the solvent (Scheme 2 and Figure 2).

A library of α-acetamide carboxamide oxindole hybrids **5** was obtained in moderate yields (26–63%), at room temperature, in short time (2 hours), proving the efficiency and the generality of this methodology. Aliphatic (**2a**, **2c** and **2m**), aromatic (**2d**, **2f**, **2g**, **2k** and **2l**), heterocyclic (**2e**, **2h**, **2i**, **2n** and **2o**), alkyne **2b** and alkene **2j** carboxylic acids were used successfully in this MCR, demonstrating a great reaction scope (Scheme 2 and Figure 2). Remarkably, the best yields were obtained when heterocyclic carboxylic acid components like 1*H*-pyrrole-3-carboxylic acid (**2n**), 2-furoic acid (**2o**) and 5-nitrofuran-2-carboxylic acid (**2e**) were used. The corresponding products **5nb**, **5ob** and **5ec** were achieved in 63, 51 and 49% yields, respectively. 1-Boc-azetidine-3-carboxylic acid (**2i**) also gave the corresponding product **5ib** in 54% yield. Considering the carbonyl component, 1-chloropropan-2-one (**3a**) was used to access the corresponding Ugi adduct **5aa** in 42% yield (Scheme 2 and Figure 2). Interestingly, *N*-benzyl-2-(*N*-(1-benzyl-3,3-dimethoxy-2-oxoindolin-5-yl)acetamido)-3-hydroxy-2-methylpropanamide (**5aa**) was obtained rather than the predictable compound with a 3-chloro-2-methylpropanamide group. We believe that a nucleophilic substitution occurs due to the presence of acetic acid (**2a**) as reaction component. Aliphatic aldehydes with small chains (**3b** and **3c**) were used successfully in the reaction approach, as expected. Also, aromatic 2-chlorobenzaldehyde (**3d**) was used and the desired compound **5ad** was obtained in 36% yield (Scheme 2 and Figure 2).

**Scheme 3:**
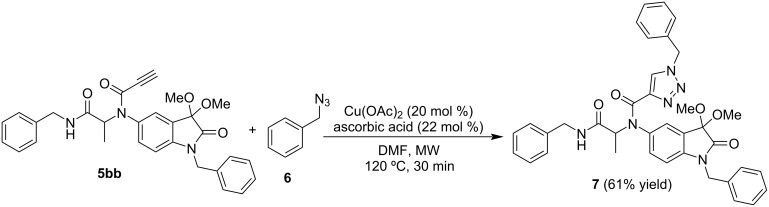
Microwave-assisted CuAAC reaction to access α-acetamide carboxamide 1,2,3-triazole oxindole hybrid **7**.

Like the oxindole scaffold, 1,2,3-triazole is also considered a privileged unit in drug discovery since compounds having this structure have a broad spectrum of biological activities, and have been widely used to create anticancer drug candidates [24–25]. The copper-catalyzed azide–alkyne cycloaddition (CuAAC) reaction, or commonly entitled “click” reaction, is a widely and straightforward tool to access the 1,2,3-triazole ring [26–27]. Due to the presence of an alkyne group on the Ugi-adduct **5bb** (Scheme 2) we decided to use the CuAAC reaction to introduce a 1,2,3-triazole unit into the scaffold. Benzyl azide (**6**), obtained using a previously reported procedure [27], was used in the CuAAC reaction. The α-acetamide carboxamide 1,2,3-triazole oxindole hybrid **7** was easily obtained in 61% yield using Cu(OAc)_2_ as catalyst, ascorbic acid, DMF as solvent, and microwave reaction conditions (120 ºC, 30 minutes) (Scheme 3).

**Scheme 4:**
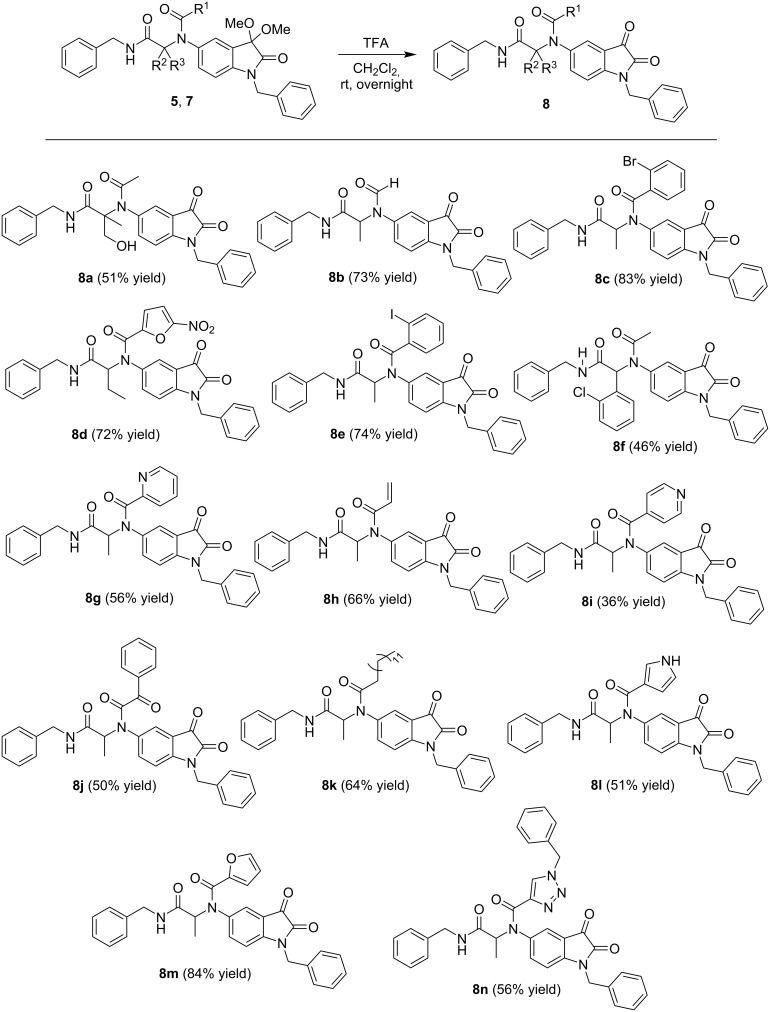
Library of α-acetamide carboxamide isatin hybrids **8** easy accessed via deprotection reaction on the Ugi-adducts **5** and **7**. TFA: trifluoroacetic acid.

Resourcefulness of the Ugi4CR and preliminary SAR studies [16] lead us to synthesize a third library of oxindole derivatives, using trifluoroacetic acid (TFA), under mild reaction conditions, to afford the corresponding α-acetamide carboxamide isatin hybrids **8** from the 3-protected oxindole counterparts **5** and **7**, in moderate to good yields (Scheme 4). The best yield was obtained when 3-protected oxindole derivatives **5** possess an aromatic or heterocyclic unit substituted in the 5-amide position of the oxindole ring. Compounds **8c**, **8d**, **8e** and **8m** were obtained in 83, 72, 74 and 84% yield, respectively. An exception was noticed for *N*-heterocycle units (pyridine and 1*H*-pyrrole) substituted in the same position, since compounds **8g**, **8l** and **8i** were obtained in 56, 51 and 36% yield, respectively. The 1,2,3-triazole hybrid isatin compound **8n** was obtained in 56% yield. Compounds **5ib** and **5hb**, with *N*-Boc protected-heterocycle units in the 5-amide position of the oxindole ring (Scheme 2) failed to afford the corresponding 3-deprotected isatin hybrids, since only decomposition byproducts (not identified) were obtained.

### Antiproliferative activity

**Figure 3:**
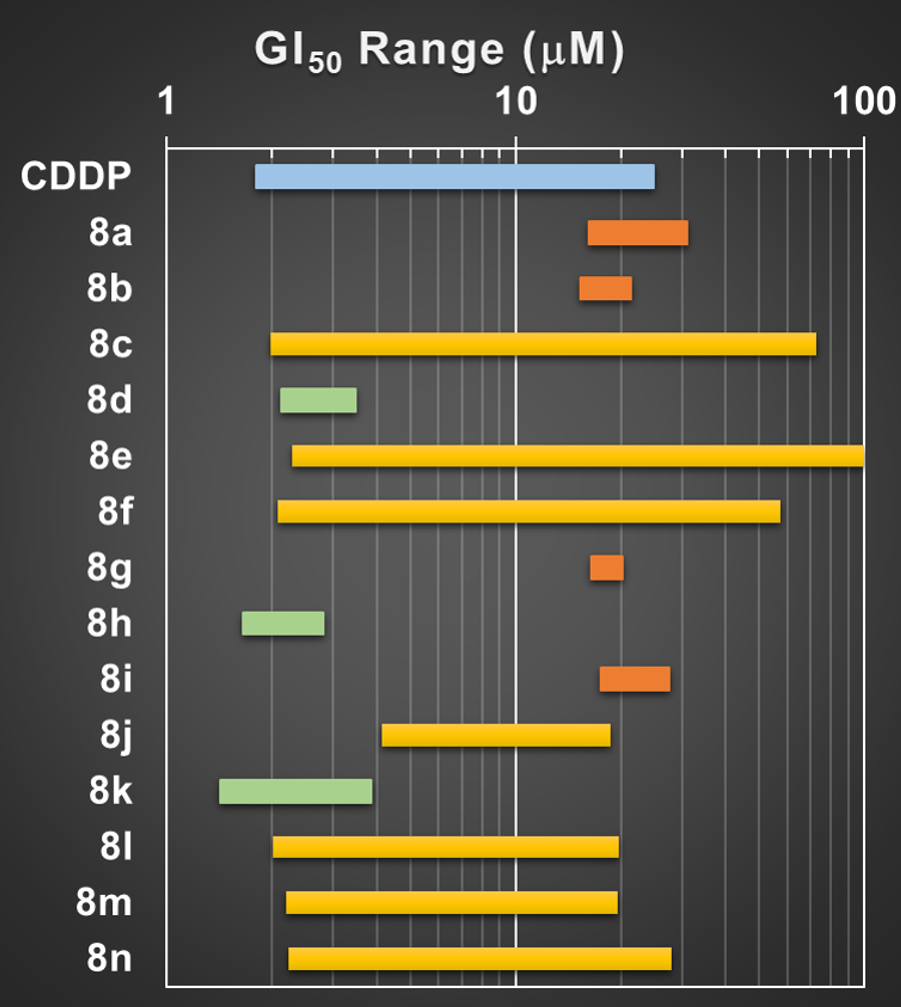
GI_50_ range plot against human solid tumor cell lines of investigated α-acetamide carboxamide isatin hybrids. Green most potent, yellow intermediate, red less potent.

Considering the potential antiproliferative activity of these compounds, we screened 14 α-acetamide carboxamide isatin hybrids against six human solid tumor cell lines. The panel of cell lines comprised non-small cell lung carcinoma A549 and SW1573, cervical adenocarcinoma HeLa, breast cancer HBL-100 and T-47D, and colon adenocarcinoma WiDr. The half-maximal growth inhibitory concentration (GI_50_) values after 48 hours of exposure were calculated for each compound (Table S1, Supporting Information File 1). The standard anticancer drug cisplatin (CDDP) was used as positive control. The results are viewed as GI_50_ range plot (Figure 3). The compounds were classified in three groups according to the GI_50_ range plot. The first group included the most active compounds **8d**, **8h** and **8k**. These compounds exhibited antiproliferative effects in the range of 1–10 µM against all cell lines. The second group comprised the less potent compounds, which were **8a**, **8b**, **8g** and **8i**. In this group, the GI_50_ values were higher than 10 µM in all cell lines tested. Finally, the third group enclosed the compounds that displayed a larger GI_50_ range with relevant activity against some cell lines (GI_50_ < 10 µM), but less potent against the others (GI_50_ > 10 µM). Some structure–activity relationships derived from the GI_50_ values. The presence of a nitro group at the furan moiety enhanced the activity (**8d** > **8m**). Presumably, the nitro group made **8d** the most potent analogue bearing an aromatic amide (**8c**, **8e**, **8g**, **8i**, **8l–n**). For the aliphatic amides, the most potent derivatives were **8h** and **8k**. The former is an α,β-unsaturated amide, which could react with nucleophiles inside the cell and thus explain its relative potency. The latter bears a long aliphatic side chain (thirteen carbon atoms), which could allow anchoring to cell membranes, representing a potential target. Overall, the results of the biological activity allow speculating that the compounds from the series **8a–n** might exhibit diverse mode of actions. Taking all these considerations as a whole, further studies of the biological activity of compounds **8d**, **8h** and **8k** might provide insights into the mode of action. Generally, the biological results point out the relevance of these isatin hybrids as privileged scaffolds for the development of new therapeutically relevant substances.

## Conclusion

Two new families of α-acetamide carboxamide oxindole and isatin hybrids were synthesized efficiently using the sustainable and efficient Ugi4CR approach. Easy access to isatin from the 3-protected oxindole scaffold was demonstrated using mild reaction conditions. Flexibility of the carboxylic acid component and also the carbonyl one (ketone/aldehyde) was exhibited in the library of Ugi adducts obtained in moderate to good yields, in a fast and clean reaction process. Among the library of α-acetamide carboxamide isatin hybrids, 14 were tested regarding their antiproliferative activity. Compounds **8d**, **8h** and **8k** were found to be the most potent ones, with GI_50_ values in the range of 1–10 μM. Further studies on the mode of action and lead-discovery are in progress and will be reported shortly.

## Supporting Information

File 1 Experimental procedures, analytical data, NMR spectra and biological assays.

## Data Availability

All data that supports the findings of this study is available in the published article and/or the supporting information to this article.
