# Supplementary material for: The Ugi4CR as effective tool to access promising anticancer isatin-based α-acetamide carboxamide oxindole hybrids
Source: Beilstein J Org Chem. 2024 May 27;20:1213–20. doi: 10.3762/bjoc.20.104 (PMC11181168; doi:10.3762/bjoc.20.104)
Supplement: File 1 — Experimental procedures, analytical data, NMR spectra and biological assays. [file Beilstein_J_Org_Chem-20-1213-s001.pdf]

BEILSTEIN JOURNAL OF ORGANIC CHEMISTRY

Supporting Information

for

The Ugi4CR as effective tool to access promising anticancer isatin-based α-acetamide carboxamide oxindole hybrids

Carolina S. Marques, Aday González-Bakker and José M. Padrón

Beilstein J. Org. Chem. **2024,** *20,* 1213–1220. doi:10.3762/bjoc.20.104

Experimental procedures, analytical data, NMR spectra and biological assays

License and Terms: This is a supporting information file under the terms of the Creative Commons Attribution License (https://creativecommons.org/licenses/by/4.0). Please note that the reuse, redistribution and reproduction in particular requires that the author(s) and source are credited and that individual graphics may be subject to special legal provisions.
The license is subject to the *Beilstein Journal of Organic Chemistry* terms and conditions: (https://www.beilstein-journals.org/bjoc/terms)

Table of Contents

Page

1. Experimental section S2

1.1. General remarks S2

1.2. 5-Amino-3-protected oxindole component S2

1.3. The Ugi4CR: general procedure S3

1.4. CuAAC reaction S7

1.5. Deprotection of methoxy group S8

2. Biological assays S11

2.1. Cell lines and culture S11

2.2. Antiproliferative tests S12

3. References S13

4. ^{1}H and ^{13}C NMR spectra S14

1. Experimental section

1.1. General remarks

All reagents were obtained from Sigma–Aldrich, Acros, Alfa Aesar and TCI and were used as received. Solvents were used as received. Reactions were conducted in round-bottom flasks, or in a Radley's® 12-position carousel reactor, or in vials or beakers. Microwave reactions were conducted with a Biotage Initator+ Microwave System with an automated position system. Column chromatography was carried out on silica gel (Carlo Erba, 40–63 µm, 60Å). Thin-layer chromatography (TLC) was carried out on aluminium-backed Kieselgel 60 F254 plates (Merck and Macherey–Nagel). Plates were visualized either by UV light or with phosphomolybdic acid in ethanol. NMR spectra were recorded with a Bruker Avance III instrument (400 MHz). Chemical shifts (δ) are given in parts per million (ppm) with respect to the solvent ($CDCl_3$, ^1H: δ = 7.26 ppm, ^{13}C: δ = 77.2 ppm). Coupling constants (*J*) are reported in Hz and refer to apparent peak multiplicities. Splitting patterns are reported as s (singlet), d (doublet), dd (doublet of doublets), t (triplet), q (quadruplet), m (multiplet), br (broad). Mass spectra (MS) were recorded with a quadrupole mass spectrometer Waters ZQ4000 (Chemistry department, University of Salamanca). The ionization was performed by ESI and the samples were infused in methanol.

Benzyl azide **6** was synthesized using a previously literature reported microwave method.[1]

NBS: *N*-bromosuccinimide; TMOF: trimethyl orthoformate; *p*TsOH: *para*-toluenesulfonic acid; DMSO: dimethyl sulfoxide; TFA: trifluoroacetic acid.

1.2. 5-Amino-3-protected oxindole component

The synthesis and characterization of 5-amino-1-benzyl-3,3-dimethoxyindolin-2-one **1** was already reported in literature by our group [2-4]. A general scheme is given in Scheme S1.

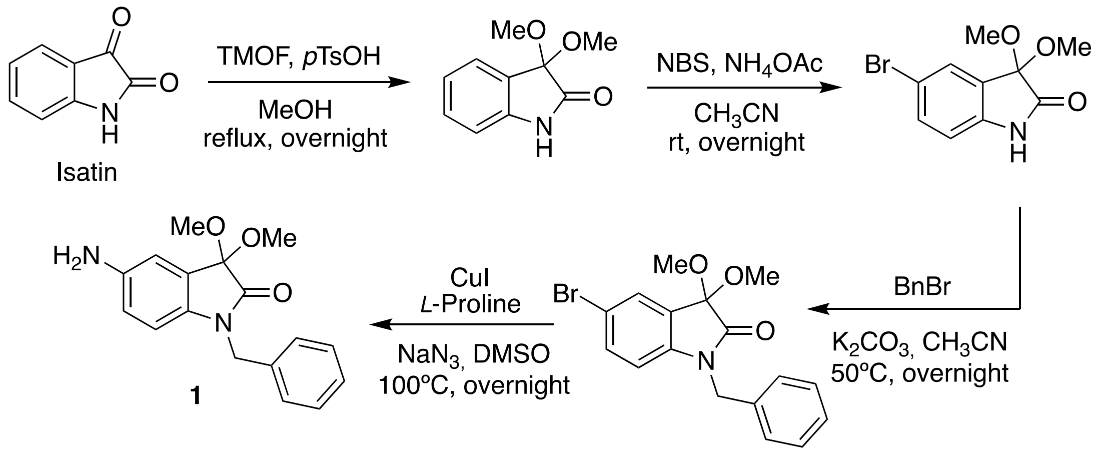

Scheme S1. Synthetic path to access 5-amino-1-benzyl-3,3-dimethoxyindolin-2-one **1**.[2-4]

1.3. The Ugi4CR: General Procedure

In a glass vial with a magnetic stirrer was added the corresponding 5-amino-1-benzyl-3,3-dimethoxyindolin-2-one **1** (1 equivalent), the carboxylic acid **2a–o** (1.5 equivalents), the ketone/aldehyde **3a–d** (1.5 equivalents), the benzyl isocyanide **4** (1.5 equivalents), ZnF_2 (10 mol %) and MeOH (2–3 mL). The vial was closed with a plastic cap and the reaction mixture was left stirring for 2 hours at room temperature. The solvent was evaporated under reduced pressure and the crude reaction mixture purified in a short chromatographic glass column with SiO_2 flash using CH_2Cl_2:AcOEt (5:1), (1:1) as eluents.

N-Benzyl-2-(N-(1-benzyl-3,3-dimethoxy-2-oxoindolin-5-yl)acetamido)-3-hydroxy-2-methylpropanamide **5aa**: **1** (90.1 mg, 0.3 mmol), **2a** (30 µL, 0.45 mmol, 1.5 equivalents), **3a** (40 µL, 0.45 mmol, 1.5 equivalents), **4** (55 µL, 0.45 mmol, 1.5 equivalents), ZnF_2 (3.1 mg, 0.03 mmol, 10 mol %) and MeOH (2 mL) were used to obtain the corresponding **5aa** as a pale yellow oil (70 mg, 42% yield). ^{1}H NMR ($CDCl_3$, 400 MHz) δ: 1.45 (s, 3H, CH_3), 1.99 (s, 3H, CH_3), 3.55 (s, 3H, OCH_3), 3.56 (s, 3H, OCH_3), 4.32-4.40 (m, 3H, CH_2), 4.48-4.54 (dd, *J*= 8 Hz, 1H, CH_2), 4.80 (s, 2H, CH_2), 6.38-6.41 (m, 1H, Ar), 6.45-6.47 (d, *J*= 8 Hz, 1H, Ar), 6.76 (m, 1H, Ar), 7.19-7.36 (m, 10H, Ar).^{13}C NMR ($CDCl_3$, 100 MHz) δ: 20.4, 20.8, 43.6, 43.7, 51.0, 51.0, 61.6, 67.5, 97.3, 110.3, 115.1, 118.2, 126.2, 127.4, 127.7, 127.8, 127.9, 128.8, 129.0, 135.5, 135.6, 138.0, 139.8, 170.5, 171.0, 172.6.

N-(1-Benzyl-3,3-dimethoxy-2-oxoindolin-5-yl)-N-(1-(benzylamino)-1-oxopropan-2-yl)propiolamide **5bb**: **1** (115.1 mg, 0.39 mmol), **2b** (36 µL, 0.58 mmol, 1.5 equivalents), **3b** (33 µL, 0.58 mmol, 1.5 equivalents), **4** (71 µL, 0.58 mmol, 1.5 equivalents), ZnF_2 (4.0 mg, 0.039 mmol, 10 mol %) and MeOH (2 mL) were used to obtain the corresponding **5bb** as a pale yellow foam type solid (92.8 mg, 47% yield). ^{1}H NMR ($CDCl_3$, 400 MHz) δ: 1.11-1.13 (*d*, *J*= 8 Hz, 3H, CH_3), 2.91 (s, 1H, CH), 3.52 (s, 3H, OCH_3), 3.58 (s, 3H, OCH_3), 4.36-4.47 (m, 2H, CH_2), 4.83 (s, 2H, CH_2), 5.05-5.10 (q, *J*= 12 Hz, 1H, CH), 6.67-6.69 (d, *J*= 8 Hz, 1H, Ar), 6.91-6.94 (m, 1H, NH), 7.09-7.12 (m, 1H, Ar), 7.23-7.34 (m, 11H, Ar).^{13}C APT NMR ($CDCl_3$, 100 MHz) δ: 14.9, 43.6, 43.7, 50.8, 50.9, 54.2, 76.0, 81.8, 96.6, 109.8, 125.5, 126.9, 127.4, 127.4, 127.6, 128.0, 128.7, 129.0, 132.2, 132.4, 134.9, 138.1, 142.9, 154.2, 170.4, 171.0.

N-Benzyl-2-(N-(1-benzyl-3,3-dimethoxy-2-oxoindolin-5-yl)formamido)propenamide **5cb**: **1** (75.9 mg, 0.25 mmol), **2c** (14 µL, 0.38 mmol, 1.5 equivalents), **3b** (21 µL, 0.38 mmol, 1.5 equivalents), **4** (46 µL, 0.38 mmol, 1.5 equivalents), ZnF_2 (2.6 mg, 0.025 mmol, 10 mol %) and MeOH (2 mL) were used to obtain the corresponding **5cb** as a pale yellow foam type solid (36 mg, 30% yield). ^{1}H NMR ($CDCl_3$, 400 MHz) δ: 1.19-1.21 (d, *J*= 8 Hz, 3H, CH_3), 3.57 (s, 3H, OCH_3), 3.60 (s, 3H, OCH_3), 4.38-4.50 (m, 2H, CH_2), 4.85 (s, 2H, CH_2), 5.05-5.10 (q, *J*= 12 Hz, 1H, CH), 6.66-6.68 (d, *J*= 8 Hz, 1H, Ar), 6.89-6.92 (s br, 1H, NH), 7.02-7.05 (m, 1H, Ar), 7.23-7.35 (m, 11H, Ar), 8.22 (s, 1H, CHO).^{13}C APT NMR ($CDCl_3$, 100 MHz) δ: 14.3, 43.7, 43.7, 50.9, 51.0, 52.4, 96.6, 110.3, 124.7, 126.2, 127.4, 127.6, 127.7, 128.1, 128.8, 129.1, 130.2, 132.4, 134.9, 138.1, 142.4, 163.6, 170.7.

N-(1-Benzyl-3,3-dimethoxy-2-oxoindolin-5-yl)-N-(1-(benzylamino)-1-oxopropan-2-yl)-2-bromobenzamide **5db**: **1** (102.9 mg, 0.35 mmol), **2d** (105 mg, 0.52 mmol, 1.5 equivalents), **3b** (23 μL, 0.52 mmol, 1.5 equivalents), **4** (63 μL, 0.52 mmol, 1.5 equivalents), ZnF_2 (3.6 mg, 0.035 mmol, 10 mol %) and MeOH (2 mL) were used to obtain the corresponding **5db** as a pale yellow foam type solid (75.9 mg, 34% yield). ^{1}H NMR ($CDCl_3$, 400 MHz) δ: 1.19-1.20 (d, *J*= 4 Hz, 3H, CH_3), 3.31 (s br, 3H, OCH_3), 3.47 (s, 3H, OCH_3), 4.34-4.39 (dd, *J*= 4 Hz, 1H, CH_2), 4.58-4.63 (dd, *J*= 4 Hz, 1H, CH_2), 4.72 (s br, 2H, CH_2), 5.35-5.40 (q, *J*= 12 Hz, 1H, CH), 6.45-6.47 (d, *J*= 8 Hz, 1H, Ar), 6.97-7.01 (t, *J*= 8 Hz, 1H, Ar), 7.05-7.06 (m, 2H, Ar), 7.17-7.19 (m, 4H, Ar), 7.24-7.34 (m, 9H, Ar).^{13}C APT NMR ($CDCl_3$, 100 MHz) δ: 14.8, 43.6, 43.8, 50.9, 54.3, 96.8, 109.6, 119.2, 125.1, 126.9, 127.3, 127.6, 127.9, 128.2, 128.4, 128.8, 128.9, 130.2, 132.6, 134.9, 138.1, 138.2, 142.3, 170.0, 170.9.

N-(1-Benzyl-3,3-dimethoxy-2-oxoindolin-5-yl)-N-(1-(benzylamino)-1-oxobutan-2-yl)-5-nitrofuran-2-carboxamide **5ec**: **1** (83.8 mg, 0.28 mmol), **2e** (66 mg, 0.42 mmol, 1.5 equivalents), **3c** (30 μL, 0.42 mmol, 1.5 equivalents), **4** (51 μL, 0.42 mmol, 1.5 equivalents), ZnF_2 (2.9 mg, 0.028 mmol, 10 mol %) and MeOH (2 mL) were used to obtain the corresponding **5ec** as a pale yellow foam type solid (83.9 mg, 49% yield). ^{1}H NMR ($CDCl_3$, 400 MHz) δ: 0.92-0.96 (m, 3H, CH_3), 1.53 (s br, 1H, CH_2), 1.71-1.73 (s br, 1H, CH_2), 3.50 (s br, 3H, OCH_3), 3.55 (s, 3H, OCH_3), 4.38-4.47 (m, 2H, CH_2), 4.85-4.89 (m, 2H, CH_2), 4.97-5.01 (q, *J*= 10 Hz, 1H, CH), 6.14 (s br, 1H, NH), 6.64-6.66 (d, *J*= 8 Hz, 1H, Ar), 7.02-7.03 (m, 2H, Ar), 7.22-7.33 (m, 12H, Ar).^{13}C APT NMR ($CDCl_3$, 100 MHz) δ: 10.9, 22.3, 43.6, 50.9, 50.9, 61.9, 96.6, 111.0, 118.5, 126.5, 127.2, 127.5, 127.7, 128.0, 128.7, 129.0, 132.1, 132.3, 134.7, 138.1, 143.3, 147.1, 151.5, 158.1, 169.5, 170.9.

N-(1-Benzyl-3,3-dimethoxy-2-oxoindolin-5-yl)-N-(1-(benzylamino)-1-oxopropan-2-yl)-2-iodobenzamide **5fb**: **1** (84.3 mg, 0.28 mmol), **2f** (104 mg, 0.42 mmol, 1.5 equivalents), **3b** (24 μL, 0.42 mmol, 1.5 equivalents), **4** (51 μL, 0.42 mmol, 1.5 equivalents), ZnF_2 (2.9 mg, 0.028 mmol, 10 mol %) and MeOH (2 mL) were used to obtain the corresponding **5fb** as a pale yellow foam type solid (65.6 mg, 34% yield). ^{1}H NMR ($CDCl_3$, 400 MHz) δ: 1.21-1.23 (d, *J*= 8 Hz, 3H, CH_3), 3.32 (s br, 3H, OCH_3), 3.49 (s, 3H, OCH_3), 4.38-4.43 (dd, *J*= 8 Hz, 1H, CH_2), 4.58-4.63 (dd, *J*= 8 Hz, 1H, CH_2), 5.35-5.41 (q, *J*= 12 Hz, 1H, CH), 6.47-6.49 (d, *J*= 8 Hz, 1H, Ar), 6.82-6.86 (t, *J*= 8 Hz, 1H, Ar), 7.07-7.13 (m, 2H, Ar), 7.16-7.20 (m, 4H, Ar), 7.27-7.37 (m, 8H, Ar), 7.57-7.59 (d, *J*= 8 Hz, 1H, Ar).^{13}C APT NMR ($CDCl_3$, 100 MHz) δ: 14.9, 43.7, 43.9, 51.0, 54.4, 93.1, 96.8, 109.6, 125.2, 127.3, 127.6, 127.7, 128.0, 128.2, 128.9, 129.0, 130.1, 132.7, 134.9, 138.3, 139.1, 141.9, 142.4, 171.0. 171.4.

N-Benzyl-2-(N-(1-benzyl-3,3-dimethoxy-2-oxoindolin-5-yl)acetamido)-2-(2-chlorophenyl)acetamide **5ad**: **1** (122.7 mg, 0.41 mmol), **2a** (35 μL, 0.62 mmol, 1.5 equivalents), **3d** (70 μL, 0.62 mmol, 1.5 equivalents), **4** (75 μL, 0.62 mmol, 1.5 equivalents), ZnF_2 (4.2 mg, 0.041 mmol, 10 mol %) and MeOH (2 mL) were used to obtain the corresponding **5ad** as a pale brown foam type solid (88.3 mg, 36% yield). ^{1}H NMR ($CDCl_3$, 400 MHz) δ: 1.86 (s, 3H, CH_3), 3.11 (s br, 3H, OCH_3), 3.54 (s, 3H, OCH_3), 4.48-4.50 (d, *J*= 8 Hz, 2H, CH_2), 4.79-4.82 (s br, 2H, CH_2), 6.16-6.19 (m, 1H, CH), 6.48 (s, 1H, Ar), 6.90-6.94 (t, *J*= 8 Hz, 1H, Ar), 7.05-7.12 (m, 2H, Ar), 7.18-7.32 (m, 13H, Ar).^{13}C APT NMR ($CDCl_3$, 100 MHz) δ: 23.3,

43.6, 43.9, 50.9, 51.0, 61.2, 96.8, 109.8, 125.4, 127.0, 127.3, 127.6, 127.7, 127.9, 128.8, 129.0, 129.6, 130.1, 131.8, 132.4, 134.9, 135.0, 135.4, 137.8, 142.2, 169.7, 171.0, 171.5.

N-(1-Benzyl-3,3-dimethoxy-2-oxoindolin-5-yl)-N-(1-(benzylamino)-1-oxopropan-2-yl)picolinamide **5gb**: **1** (200 mg, 0.67 mmol), **2g** (123.1 mg, 1.0 mmol, 1.5 equivalents), **3b** (56 µL, 1.0 mmol, 1.5 equivalents), **4** (122 µL, 1.0 mmol, 1.5 equivalents), ZnF_2 (7.0 mg, 0.067 mmol, 10 mol %) and MeOH (3 mL) were used to obtain the corresponding **5gb** as a white foam type solid (191.3 mg, 51% yield). ^{1}H NMR ($CDCl_3$, 400 MHz) δ: 1.23-1.25 (d, *J*= 8 Hz, 3H, CH_3), 3.35 (s br, 3H, OCH_3), 3.49 (s, 3H, OCH_3), 4.48-4.56 (m, 2H, CH_2), 4.76 (s br, 2H, CH_2), 5.31 (s br, 1H, CH), 6.48-6-50 (d, *J*= 8 Hz, 1H, Ar), 6.92-6.95 (m, 1H, Ar), 7.14 (s br, 2H, Ar), 7.20-7.34 (m, 12H, Ar), 7.60 (s br, 1H, NH), 8.28 (s br, 1H, Ar).^{13}C NMR ($CDCl_3$, 100 MHz) δ: 15.0, 43.6, 43.7, 50.9, 72.0, 96.8, 109.7, 125.0, 127.1, 127.3, 127.5, 127.7, 127.8, 127.9, 128.8, 129.0, 131.9, 135.0, 136.8, 138.4, 141.9, 148.4, 154.0, 171.0.

tert-Butyl 4-(2-((1-benzyl-3,3-dimethoxy-2-oxoindolin-5-yl)(1-(benzylamino)-1-oxopropan-2-yl)amino)-2-oxoethyl)piperazine-1-carboxylate **5hb**: **1** (108.6 mg, 0.36 mmol), **2h** (135 mg, 0.55 mmol, 1.5 equivalents), **3b** (31 µL, 0.55 mmol, 1.5 equivalents), **4** (67 µL, 0.55 mmol, 1.5 equivalents), ZnF_2 (3.8 mg, 0.036 mmol, 10 mol %) and MeOH (2 mL) were used to obtain the corresponding **5hb** as a pale yellow foam type solid (110.4 mg, 45% yield). ^{1}H NMR ($CDCl_3$, 400 MHz) δ: 1.42 (s, 9H, CH_3), 2.24-2.26 (m, 4H, CH_2), 3.32 (s br, 4H, CH_2), 3.45-3.62 (s br, 6H, OCH_3), 4.31-4.36 (m, 1H, CH_2), 4.46-4.48 (m, 1H, CH_2), 4.85 (s br, 2H, CH_2), 5.14-5.19 (q, *J*= 10 Hz,1H, CH), 6.65 (s br, 1H, Ar), 6.96 (s br, 2H, Ar + NH), 7.15 (m, 1H, Ar), 7.27-7.36 (m, 10H, Ar).^{13}C NMR ($CDCl_3$, 100 MHz) δ: 14.8, 28.5, 43.6, 43.7, 50.9, 53.0, 53.7, 60.4, 79.8, 96.6, 109.7, 110.5, 126.4, 126.8, 127.4, 127.5, 127.9, 128.1, 128.8, 129.1, 132.2, 134.9, 138.4, 142.7, 154.7, 170.7, 171.1.

tert-Butyl 3-((1-benzyl-3,3-dimethoxy-2-oxoindolin-5-yl)(1-(benzylamino)-1-oxopropan-2-yl)carbamoyl)azetidine-1-carboxylate **5ib**: **1** (125.1 mg, 0.42 mmol), **2i** (135 mg, 0.63 mmol, 1.5 equivalents), **3b** (35 µL, 0.63 mmol, 1.5 equivalents), **4** (77 µL, 0.63 mmol, 1.5 equivalents), ZnF_2 (4.3 mg, 0.042 mmol, 10 mol %) and MeOH (2 mL) were used to obtain the corresponding **5ib** as a pale yellow foam type solid (146.5 mg, 54% yield). ^{1}H NMR ($CDCl_3$, 400 MHz) δ: 1.04-1.10 (m, 3H, CH_3), 1.39 (s, 9H, CH_3), 3.06-3.10 (m, 1H, CH), 3.47-3.68 (m, 8H, OCH_3 + CH_2), 3.85-3.91 (m, 1H, CH_2), 4.06-4.08 (m, 1H, CH_2), 4.35-4.51 (m, 2H, CH_2), 4.81-4.87 (m, 2H, CH_2), 5.12-5.13 (m,1H, CH), 6.62-6.70 (m, 1H, Ar), 6.85-6.89 (m, 2H, Ar), 7.05 (s br, 1H, NH), 7.28-7.35 (m, 10H, Ar).^{13}C NMR ($CDCl_3$, 100 MHz) δ: 15.0, 28.4, 32.3, 43.6, 43.8, 50.9, 51.0, 54.2, 79.9, 96.4, 109.9, 110.6, 126.6, 127.5, 127.6, 127.7, 127.9, 128.1, 128.8, 129.5, 132.0, 132.2, 134.9, 138.3, 143.0, 156.1, 170.9, 171.1, 172.8.

N-(1-Benzyl-3,3-dimethoxy-2-oxoindolin-5-yl)-N-(1-(benzylamino)-1-oxopropan-2-yl)acrylamide **5jb**: **1** (122.5 mg, 0.41 mmol), **2j** (43 µL, 0.62 mmol, 1.5 equivalents), **3b** (35 µL, 0.62 mmol, 1.5 equivalents), **4** (75 µL, 0.62 mmol, 1.5 equivalents), ZnF_2 (4.2 mg, 0.041 mmol, 10 mol %) and MeOH (2 mL) were used to obtain the corresponding **5jb** as a pale yellow foam type solid (71 mg, 34%

yield). ^{1}H NMR ($CDCl_3$, 400 MHz) δ: 1.10-1.12 (d, *J*= 8 Hz, 3H, CH_3), 3.51-3.58 (m br, 6H, OCH_3), 4.41-4.45 (m, 2H, CH_2), 4.84 (s, 2H, CH_2), 5.26-5.31 (q, *J*= 12 Hz, 1H, CH), 5.53-5.56 (d, *J*= 20 Hz, 1H, CH_2), 5.89-5.95 (m, 1H, CH), 6.33-6.38 (d, *J*= 20 Hz,1H, CH), 6.66-6.68 (d, *J*= 8 Hz, 1H, Ar), 6.91-6.93 (m, 1H, NH), 7.12-7.14 (m, 2H, Ar), 7.24-7.36 (m, 10H, Ar).^{13}C APT NMR ($CDCl_3$, 100 MHz) δ: 14.8, 43.5, 43.7, 50.8, 50.9, 53.7, 96.5, 127.4, 127.5, 127.7, 128.0, 128.1, 128.4, 128.7, 128.8, 129.0, 129.1, 131.2, 132.4, 134.9, 135.0, 138.4, 142.6, 166.9, 170.9, 171.2, 171.4.

N-(1-Benzyl-3,3-dimethoxy-2-oxoindolin-5-yl)-N-(1-(benzylamino)-1-oxopropan-2-yl)isonicotinamide **5kb**: **1** (109.2 mg, 0.37 mmol), **2k** (68 mg, 0.55 mmol, 1.5 equivalents), **3b** (31 µL, 0.55 mmol, 1.5 equivalents), **4** (67 µL, 0.55 mmol, 1.5 equivalents), ZnF_2 (3.8 mg, 0.037 mmol, 10 mol %) and MeOH (2 mL) were used to obtain the corresponding **5kb** as a white foam type solid (99.5 mg, 48% yield). ^{1}H NMR ($CDCl_3$, 400 MHz) δ: 1.21-1.22 (d, *J*= 4 Hz, 3H, CH_3), 3.24 (s br, 3H, OCH_3), 3.48 (s br, 3H, OCH_3), 4.37-4.42 (dd, *J*= 8 Hz, 1H, CH_2), 4.54-4.59 (dd, *J*= 8 Hz, 1H, CH_2), 4.78 (s br, 2H, CH_2), 5.26-5.31 (q, *J*= 12 Hz, 1H, CH), 6.48-6.51 (m, 1H, Ar), 6.90-7.02 (m,1H, CH), 6.66-6.68 (d, *J*= 8 Hz, 1H, Ar), 6.91-6.93 (m, 1H, NH), 7.12-7.14 (m, 2H, Ar), 7.24-7.36 (m, 10H, Ar).^{13}C APT NMR ($CDCl_3$, 100 MHz) δ: 14.8, 43.5, 43.7, 50.8, 50.9, 53.7, 96.5, 127.4, 127.5, 127.7, 128.0, 128.1, 128.4, 128.7, 128.8, 129.0, 129.1, 131.2, 132.4, 134.9, 135.0, 138.4, 142.6, 166.9, 170.9, 171.2, 171.4.

N-Benzyl-2-(N-(1-benzyl-3,3-dimethoxy-2-oxoindolin-5-yl)-2-oxo-2-phenylacetamido)propanamide **5lb**: **1** (165.3 mg, 0.55 mmol), **2l** (125 mg, 0.83 mmol, 1.5 equivalents), **3b** (47 µL, 0.83 mmol, 1.5 equivalents), **4** (101 µL, 0.83 mmol, 1.5 equivalents), ZnF_2 (5.7 mg, 0.055 mmol, 10 mol %) and MeOH (3 mL) were used to obtain the corresponding **5lb** as a pale yellow foam type solid (156.4 mg, 48% yield). ^{1}H NMR ($CDCl_3$, 400 MHz) δ: 1.22-1.23 (d, *J*= 4 Hz, 3H, CH_3), 3.29-3.34 (m br, 6H, OCH_3), 4.41-4.46 (dd, *J*= 16 Hz, 1H, CH_2), 4.60-4.65 (dd, *J*= 8 Hz, 1H, CH_2), 4.74 (s br, 2H, CH_2), 5.13-5.18 (q, *J*= 10 Hz, 1H, CH), 6.50-6.52 (d, *J*= 8 Hz, 1H, Ar), 6.85-6.88 (m br,1H, NH), 7.11-7.13 (d, *J*= 8 Hz, 1H, Ar), 7.18-7.20 (m, 2H, Ar), 7.27-7.40 (m, 11H, Ar), 7.50-7.54 (t, *J*= 8 Hz, 1H, Ar), 7.74-7.76 (d, *J*= 8 Hz, 2H, Ar).^{13}C APT NMR ($CDCl_3$, 100 MHz) δ: 15.0, 43.6, 43.8, 50.7, 50.8, 54.7, 96.5, 109.9, 125.4, 127.2, 127.4, 127.6, 127.6, 127.8, 128.0, 128.8, 128.9, 129.0, 129.0, 129.5, 129.7, 130.4, 133.1, 133.2, 134.6, 134.8, 138.1, 143.0, 146.8, 168.4, 170.8, 170.9, 190.5.

N-(1-Benzyl-3,3-dimethoxy-2-oxoindolin-5-yl)-N-(1-(benzylamino)-1-oxopropan-2-yl)tetradecanamide **5mb**: **1** (128.1 mg, 0.43 mmol), **2m** (146 mg, 0.64 mmol, 1.5 equivalents), **3b** (36 µL, 0.64 mmol, 1.5 equivalents), **4** (78 µL, 0.64 mmol, 1.5 equivalents), ZnF_2 (4.4 mg, 0.043 mmol, 10 mol %) and MeOH (3 mL) were used to obtain the corresponding **5mb** as an orange oil (75.2 mg, 26% yield). ^{1}H NMR ($CDCl_3$, 400 MHz) δ: 0.85-0.89 (t, *J*= 8 Hz, 3H, CH_3), 1.04-1.29 (m, 22H, CH_2), 1.48-1.50 (d, *J*= 8 Hz, 3H, CH_3), 1.87-2.05 (m, 2H, CH_2), 3.44-3.58 (m br, 6H, OCH_3), 4.31-4.37 (m, 1H, CH_2), 4.45-4.52 (m, 1H, CH_2), 4.79-4.87 (m, 2H, CH_2), 5.18-5.25 (m, 1H, CH), 6.67 (s br, 1H, NH), 6.89-6.92 (m br,1H, Ar), 7.10 (m, 2H, Ar), 7.25-7.36 (m, 10H, Ar). ^{13}C APT NMR ($CDCl_3$, 100 MHz) δ: 14.2, 14.9, 22.7, 25.4, 29.3, 29.4, 29.4, 29.5, 29.7, 29.7, 29.7, 32.0, 35.0, 43.2, 43.4, 43.7, 50.9, 53.4, 70.7, 96.6, 110.5, 113.8, 117.5, 127.4, 127.4, 127.7, 127.7,

128.0, 128.7, 128.8, 128.9, 129.0, 133.2, 135.0, 138.5, 142.5, 170.9, 171.5, 174.7.

N-(1-Benzyl-3,3-dimethoxy-2-oxoindolin-5-yl)-N-(1-(benzylamino)-1-oxopropan-2-yl)-1H-pyrrole-3-carboxamide **5nb**: **1** (107.8 mg, 0.36 mmol), **2n** (60 mg, 0.54 mmol, 1.5 equivalents), **3b** (30 µL, 0.54 mmol, 1.5 equivalents), **4** (66 µL, 0.54 mmol, 1.5 equivalents), ZnF_2 (3.7 mg, 0.036 mmol, 10 mol %) and MeOH (2 mL) were used to obtain the corresponding **5nb** as pale yellow foam type solid (125.5 mg, 63% yield). ^{1}H NMR ($CDCl_3$, 400 MHz) δ: 1.18-1.20 (d, *J*= 8 Hz, 3H, CH_3), 3.50 (s, 3H, OCH_3), 3.55 (s br, 3H, OCH_3), 4.40-4.48 (m, 2H, CH_2), 4.82-4.84 (m, 1H, CH_2), 4.94-4.98 (m, 1H, CH_2), 5.36-5.38 (m, 1H, CH), 5.92-5.95 (m, 1H, CH), 6.70-6.72 (d, *J*= 8 Hz,1H, Ar), 6.82-6.83 (m, 1H, CH), 7.07-7.09 (m, 1H, CH), 7.22-7.38 (m, 12H, Ar), 9.72 (s br, 1H, NH). ^{13}C APT NMR ($CDCl_3$, 100 MHz) δ: 15.0, 43.6, 43.8, 50.9, 51.0, 54.5, 96.8, 110.4, 114.6, 121.9, 124.5, 127.4, 127.5, 127.7, 128.1, 128.7, 129.1, 133.2, 135.0, 138.4, 143.1, 162.4, 171.1, 171.4.

N-(1-Benzyl-3,3-dimethoxy-2-oxoindolin-5-yl)-N-(1-(benzylamino)-1-oxopropan-2-yl)furan-2-carboxamide **5ob**: **1** (144.1 mg, 0.48 mmol), **2o** (81 mg, 0.72 mmol, 1.5 equivalents), **3b** (40 µL, 0.72 mmol, 1.5 equivalents), **4** (88 µL, 0.72 mmol, 1.5 equivalents), ZnF_2 (5.0 mg, 0.048 mmol, 10 mol %) and MeOH (3 mL) were used to obtain the corresponding **5ob** as pale brown foam type solid (136.1 mg, 51% yield). ^{1}H NMR ($CDCl_3$, 400 MHz) δ: 1.17-1.19 (d, *J*= 8 Hz, 3H, CH_3), 3.47 (s, 3H, OCH_3), 3.53 (s, 3H, OCH_3), 4.42-4.43 (d, *J*= 4 Hz, 2H, CH_2), 4.81-4.86 (m br, 2H, CH_2), 5.32-5.37 (q, *J*= 12 Hz, 1H, CH_2), 5.64-5.65 (d, *J*= 4 Hz, 1H, CH), 6.19-6.20 (m, 1H, CH), 6.66-6.68 (d, *J*= 8 Hz,1H, Ar), 7.01-7.04 (m, 1H, CH), 7.17-7.35 (m, 12H, Ar).^{13}C APT NMR ($CDCl_3$, 100 MHz) δ: 14.9, 43.5, 43.7, 50.8, 50.8, 54.5, 96.6, 110.1, 111.4, 117.4, 127.2, 127.3, 127.3, 127.7, 128.0, 128.6, 129.0, 132.6, 132.9, 134.9, 138.3, 142.9, 145.1, 146.4, 160.1, 171.0, 171.0.

1.4. CuAAC reaction

In a microwave vial was added **5bb** (328.8 mg, 0.64 mmol), benzyl azide (**6**, 128 mg, 0.96 mmol, 1.5 equivalents), $Cu(OAc)_2$ (23 mg, 0.13 mmol, 20 mol %), ascorbic acid (25 mg, 0.14 mmol, 22 mol %) and DMF (2 mL). The vial was heated in the microwave oven at 120 ºC for 30 minutes. The solvent was evaporated under reduced pressure and the crude reaction mixture purified in a short chromatographic glass column with SiO_2 flash using Hex:AcOEt (1:1), (1/2) and AcOEt as eluents.

1-Benzyl-N-(1-benzyl-3,3-dimethoxy-2-oxoindolin-5-yl)-N-(1-(benzylamino)-1-oxopropan-2-yl)-1H-1,2,3-triazole-4-carboxamide **7** was obtained as pale brown foam type solid (251.8 mg, 61% yield). ^{1}H NMR ($CDCl_3$, 400 MHz) δ: 1.17-1.19 (d, *J*= 8 Hz, 3H, CH_3), 3.42 (s, 3H, OCH_3), 3.49 (s br, 3H, OCH_3), 4.41-4.42 (d, *J*= 4 Hz, 2H, CH_2), 4.71-5.00 (m NH), 6.96-6.99 (m,1H, Ar), 7.09-7.35 (m, 17H, CH + Ar).^{13}C NMR ($CDCl_3$, 100 MHz) δ: 15.0, 43.5, 43.7, 50.8, 50.9, 54.2, 96.6, 109.8, 125.5, 126.2, 127.4, 127.4, 127.7, 128.0, 128.4, 128.7, 129.1, 129.2, 132.9, 133.5, 134.9, 138.3, 142.6, 162.0, 162.6, 170.9, 171.0.

1.5. Deprotection of methoxy group

In a round-bottom flask with a magnetic stirrer was added the corresponding α-acetoamide carboxamide oxindole hybrids **5** and **7**, CH_2Cl_2 (5 mL) and trifluoroacetic acid (1 mL). The mixture was stirred overnight at room temperature. The reaction was quenched with saturated $NaHCO_3$ aqueous solution, carefully, to neutralize the acid. The resulting crude mixture was extracted with CH_2Cl_2. The combined organic layers were dried with $MgSO_4$, filtered and the solvent evaporated under reduced pressure. The crude reaction mixture was purified in a short chromatographic glass column with SiO_2 flash using Hex:AcOEt (5:1), (1:1), (1:2).

N-Benzyl-2-(N-(1-benzyl-2,3-dioxoindolin-5-yl)acetamido)-3-hydroxy-2-methylpropanamide **8a**: **5aa** (70 mg, 0.13 mmol), CH_2Cl_2 (5 mL) and TFA (1 mL) were used to obtain the corresponding **8a** as pale purple foam type solid (32.7 mg, 51% yield).^{1}H NMR (DMSO-d_6, 400 MHz) δ: 1.38 (s, 3H, CH_3), 1.95 (s, 3H, CH_3), 4.16-4.34 (m, 4H, CH_2), 4.77-4.86 (m, 2H, CH_2), 6.04 (s br, 1H, NH), 6.68-6.73 (m, 2H, Ar), 6.76 (s, 1H, Ar), 7.13 (m, 4H, Ar), 7.30-7.41 (m, 5H, Ar), 8.62-8.65 (m, 1H, Ar). ^{13}C APT NMR (DMSO-d_6, 100 MHz) δ: 20.7, 21.9, 42.4, 42.9, 59.3, 64.9, 110.4, 111.5, 117.7, 123.5, 126.6, 127.2, 127.5, 127.6, 128.1, 128.7, 135.9, 139.4, 141.4, 142.3, 158.1, 170.2, 172.9, 183.9. HRMS (ESI) m/z: calculated for $C_{28}H_{27}O_5N_3{}^{23}Na$ [M]$^+$ 508.18429, found 508.1837.

N-Benzyl-2-(N-(1-benzyl-2,3-dioxoindolin-5-yl)formamido)propenamide **8b**: **5cb** (268.5 mg, 0.55 mmol), CH_2Cl_2 (5 mL) and TFA (1 mL) were used to obtain the corresponding **8b** as an orange foam type solid (177.8 mg, 73% yield). ^{1}H NMR ($CDCl_3$, 400 MHz) δ: 1.21-1.22 (d, *J*= 8 Hz, 3H, CH_3), 4.38-4.49 (m, 2H, CH_2), 4.89-4.97 (m, 2H, CH_2), 5.04-5.09 (q, *J*= 8 Hz, 1H, CH), 6.77-6.79 (m, 2H, Ar + NH), 7.24-7.38 (m, 11H, Ar), 7.43 (s, 1H, Ar), 8.19 (s, 1H, CH). ^{13}C NMR ($CDCl_3$, 100 MHz) δ: 14.5, 43.8, 44.4, 52.4, 111.9, 118.0, 124.6, 127.7, 127.7, 127.8, 128.6, 128.9, 129.3, 133.8, 134.10, 137.7, 150.3, 158.1, 163.1, 170.4, 182.4. HRMS (ESI) m/z: calculated for $C_{26}H_{24}O_4N_3$ [M]$^+$ 442.17613, found 442.1755.

N-(1-Benzyl-2,3-dioxoindolin-5-yl)-N-(1-(benzylamino)-1-oxopropan-2-yl)-2-bromobenzamide **8c**: **5db** (62.2 mg, 0.097 mmol), CH_2Cl_2 (5 mL) and TFA (1 mL) were used to obtain the corresponding **8c** as an orange foam type solid (47.9 mg, 83% yield).^{1}H NMR ($CDCl_3$, 400 MHz) δ: 1.19-1.21 (d, *J*= 8 Hz, 3H, CH_3), 4.33-4.38 (dd, *J*= 16 Hz, 1H, CH_2), 4.58-4.63 (dd, *J*= 16 Hz, 1H, CH_2), 4.78-4.82 (m, 2H, CH_2), 5.37-5.39 (m, 1H, CH), 6.52-6.54 (d, *J*= 8 Hz, 1H, Ar), 7.01-7.05 (m, 1H, Ar), 7.09-7.11 (m, 2H, Ar + NH), 7.23-7.34 (m, 14H, Ar). ^{13}C APT NMR ($CDCl_3$, 100 MHz) δ: 14.9, 43.9, 44.3, 54.4, 111.1, 117.4, 119.1, 126.6, 127.2, 127.5, 127.7, 128.4, 128.4, 128.9, 129.2, 130.5, 132.8, 134.1, 137.5, 138.1, 139.5, 150.1, 158.1, 169.8, 182.2. HRMS (ESI) m/z: calculated for $C_{32}H_{26}O_4N_3{}^{79}Br^{23}Na$ [M]$^+$ 618.09989, found 618.0992.

N-(1-Benzyl-2,3-dioxoindolin-5-yl)-N-(1-(benzylamino)-1-oxobutan-2-yl)-5-nitrofuran-2-carboxamide **8d**: **5ec** (62.4 mg, 0.10 mmol), CH_2Cl_2 (5 mL) and TFA (1 mL) were used to obtain the corresponding **8d** as an orange foam type solid (41.6 mg, 72% yield).^{1}H NMR ($CDCl_3$, 400 MHz) δ: 0.94-0.97 (m, 3H, CH_3), 1.53-1.59 (m, 1H, CH_2), 1.73-1.74 (m, 1H, CH_2), 4.39-4.50 (m, 2H, CH_2), 4.91-4.95 (m,

3H, CH + CH_2), 6.75-6.79 (m, 2H, Ar), 6.86 (s br, 1H, NH), 7.08-7.09 (d, *J*= 4 Hz, 1H, CH), 7.24-7.39 (m, 11H, Ar + CH), 7.43 (s br, 1H, Ar). ^{13}C APT NMR ($CDCl_3$, 100 MHz) δ: 10.8, 22.5, 43.8, 44.4, 61.8, 111.1, 119.6, 126.6, 127.4, 127.7, 127.9, 128.4, 128.8, 129.3, 134.0, 138.0, 139.7, 147.0, 151.2, 151.5, 157.9, 158.2, 169.2, 182.4. HRMS (ESI) m/z: calculated for $C_{31}H_{27}O_7N_4$ $[M]^+$ 567.18743, found 567.1866.

N-(1-Benzyl-2,3-dioxoindolin-5-yl)-N-(1-(benzylamino)-1-oxopropan-2-yl)-2-iodobenzamide **8e**: **5fb** (86.1 mg, 0.125 mmol), CH_2Cl_2 (5 mL) and TFA (1 mL) were used to obtain the corresponding **8e** as an orange foam type solid (59 mg, 74% yield).^{1}H NMR ($CDCl_3$, 400 MHz) δ: 1.20-1.22 (d, *J*= 8 Hz, 3H, CH_3), 4.36-4.41 (dd, *J*= 14 Hz, 1H, CH_2), 4.58-4.63 (dd, *J*= 14 Hz, 1H, CH_2), 4.76-4.83 (m, 2H, CH_2), 5.35-5.41 (q, *J*= 8 Hz, 1H, CH), 6.53-6.56 (m, 1H, Ar), 6.84-6.88 (t, *J*= 8 Hz, 1H, Ar), 7.12-7.14 (m br, 2H, Ar + NH), 7.23-7.35 (m, 13H, Ar), 7.56-7.58 (d, *J*= 8 Hz, 1H, Ar). ^{13}C APT NMR ($CDCl_3$, 100 MHz) δ: 14.9, 43.9, 44.3, 54.1, 92.9, 111.1, 117.4, 126.9, 127.6, 127.8, 127.8, 128.2, 128.5, 129.0, 129.2, 130.4, 134.1, 138.2, 139.3, 139.7, 141.4, 150.1, 158.2, 171.2, 182.2. HRMS (ESI) m/z: calculated for $C_{32}H_{26}O_4N_3{}^{127}I^{23}Na$ $[M]^+$ 666.08602, found 666.0850.

N-Benzyl-2-(N-(1-benzyl-2,3-dioxoindolin-5-yl)acetamido)-2-(2-chlorophenyl)acetamide **8f**: **5ad** (109.7 mg, 0.18 mmol), CH_2Cl_2 (5 mL) and TFA (1 mL) were used to obtain the corresponding **8f** as a pale yellow foam type solid (47.1 mg, 46% yield).^{1}H NMR ($CDCl_3$, 400 MHz) δ: 1.86 (s, 3H, CH_3), 4.48-4.49 (m, 2H, CH_2), 4.85 (m br, 2H, CH_2), 6.16 (s br, 1H, CH), 6.47 (s, 1H, Ar), 6.59 (s br, 1H, Ar), 6.92-6.95 (m, 1H, Ar), 6.99-7.01 (m, 1H, Ar), 7.10-7.14 (t, *J*= 8 Hz, 1H, Ar), 7.21-7.36 (m, 12H, Ar), 7.98 (s br, 1H, NH). ^{13}C APT NMR ($CDCl_3$, 100 MHz) δ: 23.2, 44.0, 44.3, 61.2, 111.2, 117.6, 127.1, 127.6, 127.7, 127.8, 128.4, 128.8, 129.2, 130.0, 130.4, 131.5, 132.0, 134.2, 135.3, 136.2, 137.7, 150.0, 158.3, 169.5, 170.9, 182.4. HRMS (ESI) m/z: calculated for $C_{32}H_{26}O_4N_3{}^{35}Cl^{23}Na$ $[M]^+$ 574.15041, found 574.1495.

N-(1-Benzyl-2,3-dioxoindolin-5-yl)-N-(1-(benzylamino)-1-oxopropan-2-yl)picolinamide **8g**: **5gb** (165.9 mg, 0.29 mmol), CH_2Cl_2 (5 mL) and TFA (1 mL) were used to obtain the corresponding **8g** as an orange foam type solid (84.6 mg, 56% yield).^{1}H NMR ($CDCl_3$, 400 MHz) δ: 1.23-1.24 (d, *J*= 4 Hz, 3H, CH_3), 4.49-4.53 (m, 2H, CH_2), 4.86 (s, 2H, CH_2), 5.24 (s br, 1H, CH), 6.60-6.62 (d, *J*= 8 Hz, 1H, Ar), 7.18-7.22 (m, 2H, Ar + NH), 7.27-7.38 (m, 12H, Ar), 7.55 (s br, 1H, Ar), 7.72 (s br, 1H, Ar), 8.24-8.25 (d, *J*= 4 Hz, 1H, Ar). ^{13}C NMR ($CDCl_3$, 100 MHz) δ: 15.2, 43.8, 44.3, 50.9, 111.3, 117.5, 124.6, 125.0, 126.9, 127.4, 127.6, 127.9, 128.0, 128.4, 128.9, 129.0, 129.2, 134.2, 137.6, 138.3, 139.7, 147.8, 149.9, 152.9, 158.3, 168.9, 170.4, 182.5. HRMS (ESI) m/z: calculated for $C_{31}H_{27}O_4N_4$ $[M]^+$ 519.20268, found 519.2023.

N-(1-Benzyl-2,3-dioxoindolin-5-yl)-N-(1-(benzylamino)-1-oxopropan-2-yl)acrylamide **8h**: **5jb** (71.0 mg, 0.14 mmol), CH_2Cl_2 (5 mL) and TFA (1 mL) were used to obtain the corresponding **8h** as an orange foam type solid (43.0 mg, 66% yield).^{1}H NMR ($CDCl_3$, 400 MHz) δ: 1.12-1.13 (d, *J*= 4 Hz, 3H, CH_3), 4.34-4.48 (m, 3H, CH_2 + CH), 4.93 (s, 2H, CH_2), 5.23-5.28 (q, *J*= 12 Hz, 1H, CH), 5.56-5.59 (d, *J*= 12 Hz, 1H, CH_2), 5.79-5.89 (m, 1H, CH_2), 6.31-6.31 (m, 1H, Ar), 6.75-6.77 (d, *J*= 8 Hz, 1H, Ar), 6.84 (s br, 1H, NH), 6.97 (m br, 1H, Ar), 7.17-7.19 (d, *J*= 8

Hz, 1H, Ar), 7.23-7.41 (m, 10H, Ar). ^{13}C APT NMR ($CDCl_3$, 100 MHz) δ: 15.0, 43.7, 44.5, 53.7, 111.9, 126.8, 126.9, 127.6, 127.8, 127.9, 128.0, 128.0, 128.6, 128.9, 129.3, 129.9, 131.6, 133.9, 134.1, 134.2, 138.2, 138.2, 139.8, 150.6, 150.7, 158.1, 166.7, 170.9, 171.1, 182.4. HRMS (ESI) m/z: calculated for $C_{28}H_{24}O_4N_3$ $[M]^-$ 466.17723, found 466.1775.

N-(1-Benzyl-2,3-dioxoindolin-5-yl)-N-(1-(benzylamino)-1-oxopropan-2-yl)isonicotinamide **8i**: **5kb** (75.5 mg, 0.13 mmol), CH_2Cl_2 (5 mL) and TFA (1 mL) were used to obtain the corresponding **8i** as an orange foam type solid (24.2 mg, 36% yield). ^1H NMR ($CDCl_3$, 400 MHz) δ: 1.22-1.24 (d, *J*= 8 Hz, 3H, CH_3), 4.36-4.41 (dd, *J*= 14 Hz, 1H, CH_2), 4.52-4.57 (dd, *J*= 14 Hz, 1H, CH_2), 4.85 (s br, 2H, CH_2), 5.24-5.29 (m br, 1H, CH), 6.57-6.59 (d, *J*= 8 Hz, 1H, Ar), 6.83 (s br, 1H, NH), 7.03 (s br, 2H, Ar), 7.18 (s br, 1H, Ar), 7.25-7.37 (m, 11H, Ar), 8.46 (s br, 2H, Ar). ^{13}C NMR ($CDCl_3$, 100 MHz) δ: 15.5, 43.8, 44.5, 55.0, 111.7, 117.8, 122.2, 126.4, 127.6, 127.8, 128.0, 128.6, 129.0, 129.3, 134.0, 134.2, 138.1, 139.9, 144.0, 149.2, 150.3, 158.0, 169.0, 170.5, 182.3. HRMS (ESI) m/z: calculated for $C_{31}H_{27}O_4N_4$ $[M]^+$ 519.20268, found 519.2024.

N-Benzyl-2-(N-(1-benzyl-2,3-dioxoindolin-5-yl)-2-oxo-2-phenylacetamido)propenamide **8j**: **5lb** (156.0 mg, 0.26 mmol), CH_2Cl_2 (5 mL) and TFA (1 mL) were used to obtain the corresponding **8j** as a dark orange foam type solid (71.0 mg, 50% yield). ^1H NMR ($CDCl_3$, 400 MHz) δ: 1.20-1.22 (d, *J*= 8 Hz, 3H, CH_3), 4.38-4.43 (dd, *J*= 14 Hz, 1H, CH_2), 4.56-4.61 (dd, *J*= 14 Hz, 1H, CH_2), 4.75-4.85 (q, *J*= 14 Hz, 2H, CH_2), 5.12-5.18 (q, *J*= 8 Hz, 1H, CH), 6.58-6.60 (d, *J*= 8 Hz, 1H, Ar), 6.87-6.90 (m, 1H, Ar), 7.23-7.42 (m, 13H, Ar), 7.47 (s br, 1H, NH), 7.53-7.56 (m, 1H, Ar), 7.72-7.74 (d, *J*= 8 Hz, 2H, Ar). ^{13}C NMR ($CDCl_3$, 100 MHz) δ: 15.3, 43.8, 44.3, 54.6, 111.4, 117.6, 127.5, 127.6, 127.7, 127.8, 128.4, 128.9, 129.1, 129.2, 129.5, 131.8, 132.9, 134.0, 135.0, 138.0, 140.7, 150.7, 158.1, 167.9, 170.6, 182.0, 190.2. HRMS (ESI) m/z: calculated for $C_{33}H_{28}O_5N_3$ $[M]^+$ 546.20235, found 546.2017.

N-(1-Benzyl-2,3-dioxoindolin-5-yl)-N-(1-(benzylamino)-1-oxopropan-2-yl)tetradecanamide **8k**: **5mb** (63.7 mg, 0.095 mmol), CH_2Cl_2 (5 mL) and TFA (1 mL) were used to obtain the corresponding **8k** as an orange oil (38.2 mg, 64% yield). ^1H NMR ($CDCl_3$, 400 MHz) δ: 0.85-0.89 (t, *J*= 8 Hz, 3H, CH_3), 1.04-1.28 (m, 22H, CH_2), 1.42-1.51 (m, 3H, CH_3), 1.84-1.95 (m, 2H, CH_2), 4.32-4.37 (dd, *J*= 14 Hz, 1H, CH_2), 4.45-4.50 (m, 1H, CH_2), 4.92 (s br, 2H, CH_2), 5.15-5.21 (q, *J*= 8 Hz, 1H, CH), 6.75-6.77 (d, *J*= 8 Hz, 1H, Ar), 6.98 (s br, 1H, NH), 7.17-7.19 (d, *J*= 8 Hz, 1H, Ar), 7.25-7.40 (m, 11H, Ar). ^{13}C NMR ($CDCl_3$, 100 MHz) δ: 14.2, 15.1, 22.8, 25.3, 29.3, 29.4, 29.5, 29.7, 29.7, 29.8, 32.0, 35.1, 43.3, 43.6, 44.5, 70.8, 126.8, 127.6, 127.7, 127.8, 127.9, 128.5, 128.8, 128.9, 129.1, 129.3, 134.2, 134.6, 137.9, 138.3, 139.7, 150.4, 158.2, 169.6, 170.4, 171.3, 174.3. HRMS (ESI) m/z: calculated for $C_{39}H_{49}O_4N_3{}^{23}Na$ $[M]^+$ 646.36153, found 646.3605.

N-(1-Benzyl-2,3-dioxoindolin-5-yl)-N-(1-(benzylamino)-1-oxopropan-2-yl)-1H-pyrrole-3-carboxamide **8l**: **5nb** (91.8 mg, 0.17 mmol), CH_2Cl_2 (5 mL) and TFA (1 mL) were used to obtain the corresponding **8l** as an orange foam type solid (43.1 mg, 51% yield). ^1H NMR ($CDCl_3$, 400 MHz) δ: 1.18-1.20 (d, *J*= 8 Hz, 3H, CH_3), 4.43-4.46 (m, 2H, CH_2), 4.91-4.98 (m, 3H, CH_2 + CH), 5.32-5.37 (q, *J*= 8 Hz, 1H, CH), 5.94-5.95 (m, 1H, CH), 6.78-6.81 (m, 1H, Ar), 6.85 (s br, 1H, CH), 6.97 (s

br, 1H, NH), 7.25-7.43 (m, 12H, Ar), 9.53 (s br, 1H, NH). ^{13}C APT NMR ($CDCl_3$, 100 MHz) δ: 15.1, 43.7, 44.6, 54.3, 110.6, 112.3, 114.7, 122.2, 124.3, 127.6, 127.7, 127.9, 128.6, 128.9, 129.4, 134.2, 134.7, 138.2, 140.7, 150.9, 158.3, 162.1, 171.0, 182.4. HRMS (ESI) m/z: calculated for $C_{30}H_{26}O_4N_4{}^{23}Na$ $[M]^+$ 529.18463, found 529.1840.

N-(1-Benzyl-2,3-dioxoindolin-5-yl)-N-(1-(benzylamino)-1-oxopropan-2-yl)furan-2-carboxamide **8m**: **5ob** (130.0 mg, 0.23 mmol), CH_2Cl_2 (5 mL) and TFA (1 mL) were used to obtain the corresponding **8m** as a dark orange oil (98.1 mg, 84% yield).^{1}H NMR ($CDCl_3$, 400 MHz) δ: 1.19-1.22 (m, 3H, CH_3), 4.42-4.44 (m, 2H, CH_2), 4.89-4.99 (m, 2H, CH_2), 5.28-5.34 (q, *J*= 8 Hz, 1H, CH), 6.11-6.12 (d, *J*= 4 Hz, 1H, CH), 6.25-6.26 (m, 1H, CH), 6.73-6.75 (d, *J*= 8 Hz, 1H, Ar), 7.00-7.03 (m, 1H, NH), 7.22-7.41 (m, 13H, Ar). ^{13}C NMR ($CDCl_3$, 100 MHz) δ: 15.1, 43.7, 44.5, 54.5, 111.6, 111.7, 117.9, 118.3, 127.2, 127.6, 127.7, 127.9, 128.6, 128.8, 129.3, 134.2, 134.5, 138.2, 140.1, 145.4, 146.4, 150.6, 158.3, 159.9, 170.7, 182.4. HRMS (ESI) m/z: calculated for $C_{30}H_{25}O_5N_3{}^{23}Na$ $[M]^+$ 530.16864, found 530.1679.

1-Benzyl-N-(1-benzyl-2,3-dioxoindolin-5-yl)-N-(1-(benzylamino)-1-oxopropan-2-yl)-1H-1,2,3-triazole-4-carboxamide **8n**: **7** (214.7 mg, 0.33 mmol), CH_2Cl_2 (5 mL) and TFA (1 mL) were used to obtain the corresponding **8n** as an orange foam type solid (112.5 mg, 56% yield).^{1}H NMR ($CDCl_3$, 400 MHz) δ: 1.20-1.22 (d, *J*= 8 Hz, 3H, CH_3), 4.42-4.43 (d, *J*= 4 Hz, 2H, CH_2), 4.89 (s br, 2H, CH_2), 5.31-5.39 (m, 3H, CH_2 + CH), 6.69-6.71 (d, *J*= 8 Hz, 1H, Ar), 6.98 (s br, 1H, NH), 7.18-7.39 (m, 17H, Ar + CH), 7.55 (s br, 1H, Ar). ^{13}C NMR ($CDCl_3$, 100 MHz) δ: 14.2, 43.7, 44.4, 54.4, 54.9, 111.3, 117.6, 127.3, 127.5, 127.7, 127.9, 128.5, 128.8, 129.3, 129.3, 129.4, 133.5, 134.3, 138.2, 140.2, 143.2, 150.4, 158.4, 161.8, 170.6, 182.5. HRMS (ESI) m/z: calculated for $C_{35}H_{31}O_4N_6$ $[M]^+$ 599.24013, found 599.2390.

2. Biological assays

2.1. Cell lines and culture

The human solid tumor cell lines used in this study were the non-small cell lung cancer A549 and SW1573, the cervix cancer HeLa, the breast cancer cell lines HBL-100 and T-47D, and the colon cancer WiDr. Cell lines were obtained from Prof. Godefridus J. Peters (VUmc, Amsterdam, NL). The maintenance of cell cultures was in 60 mm Petri dishes in a humidified air incubator (37 ºC, 5% CO_2, 95% humidity). The cell culture medium used was RPMI 1640 supplemented with 5% heat inactivated FCS, 2 mM L-glutamine, 100 U/mL penicillin and 0.1 mg/mL streptomycin. Cell cultures were passaged biweekly using 0.05% trypsin and maintained at low passage. Single cell suspensions were counted using Moxi Z automated cell counter.

2.2. Antiproliferative tests

On day 0, cells were inoculated in a volume of 100 µL per well at densities of 2500 (A549, HBL-100, HeLa and SW1573) or 5000 (T-47D and WiDr) cells per well, based on their doubling times. Stock solutions of isatins **8a**–**n** were prepared in DMSO at 40 mM. Each compound was tested in triplicate at different dilutions in the range 1–100 µM. Cisplatin served as positive control. Control cells received an equivalent concentration of DMSO (0.25% v/v, negative control). The drug treatment was started on day 1 after plating and incubation times was 48 h. Then, the SRB colorimetric method of the NCI was performed [5]. The optical density (OD) of each well was measured at 530 nm, using BioTek's PowerWave XS Absorbance Microplate Reader. Values were corrected for background OD from wells only containing medium. Antiproliferative activity of the compounds expressed as GI_{50} was calculated according to NCI formulas [6].

Table S1. Antiproliferative activity (GI_{50}, µM) against human solid tumor cell lines.

		Cell line (origin)					
Compound		A549 (lung)	HBL-100 (breast)	HeLa (cervix)	SW1573 (lung)	T-47D (breast)	WiDr (colon)
**8a**		30±7.6	23±7.9	16±6.2	25±6.6	31±1.6	23±1.7
**8b**		21±1.3	21±2.7	15±5.1	18±4.0	21±1.2	20±1.7
**8c**		73±9.1	2.1±0.54	3.5±1.7	2.0±0.22	10±3.9	10±2.0
**8d**		2.2±0.35	2.1±0.08	2.2±0.18	3.4±1.2	3.5±0.22	2.5±0.33
**8e**		>100	2.3±0.41	2.8±0.89	2.8±0.13	12±3.6	15±6.1
**8f**		57±11	2.1±0.06	2.2±0.64	2.7±0.54	14±2.2	7.3±2.9
**8g**		20±0.77	19±2.0	17±6.6	18±4.1	20±2.1	16±3.2
**8h**		2.8±0.52	2.0±0.33	1.7±0.49	1.8±0.19	2.8±0.16	2.5±0.54
**8i**		27±13	18±5.0	18±8.4	20±2.8	26±8.9	26±7.3
**8j**		9.7±0.94	5.5±1.2	4.1±1.8	5.0±1.8	19±1.4	12±4.1
**8k**		3.6±0.76	1.9±0.19	1.4±0.18	1.7±0.8	3.8±0.31	3.3±0.57
**8l**		20±3.3	2.0±0.25	2.4±0.77	3.9±1.3	6.5±2.0	11±4.1
**8m**		20±8.8	2.2±0.11	2.6±0.58	3.9±0.31	3.8±0.81	5.2±1.7
**8n**		28±6.1	2.2±0.36	3.0±1.1	3.2±1.2	9.0±3.2	8.8±3.2
CDDP		4.9±0.18	1.9±0.16	1.8±0.52	2.7±0.38	17±3.3	23±4.3

Values represent mean ± standard deviation of three independent experiments.

3. References

[1] Marques, C. S.; Burke, A. J. *ChemCatChem* **2016**, 8, 3518-3526. Doi: 10.1002/cctc.201600901.
[2] Marques, C. S.; López, Ó.; Leitzbach, L.; Fernández-Bolanõs, J. G.; Stark, H.; Burke, A. J. *Synthesis* **2022**, 54, 4304. Doi: 10.1055/s-0041-1737343.
[3] Marques, C. S.; McArdle, P.; Erxleben, A.; Burke, A. J. *Eur. J. Org. Chem*. **2020**, 3622. Doi: 10.1002/ejoc.202000334.
[4] Marques, C. S.; González-Bakker, A.; Padrón, J. M.; Burke, A. J. *New J. Chem.* **2023**, 47, 743-750. Doi: 10.1039/d2nj03627d.
[5] Orellana, E. A.; Kasinski, A. L. *Bio-protocol.* **2016**, 6, e1984. Doi: 10.21769/BioProtoc.1984.
[6] Monks, A.; Scudiero, D.; Skehan, P.; Shoemaker, R.; Paull, K.; Vistica, D.; Hose, C.; Langley, J.; Cronise, P.; Vaigro-wolff, A.; *et al. J. Natl. Cancer Inst.* **1991**, 83, 757-766. Doi: 10.1093/jnci/83.11.757.

4. ^{1}H and ^{13}C NMR spectra

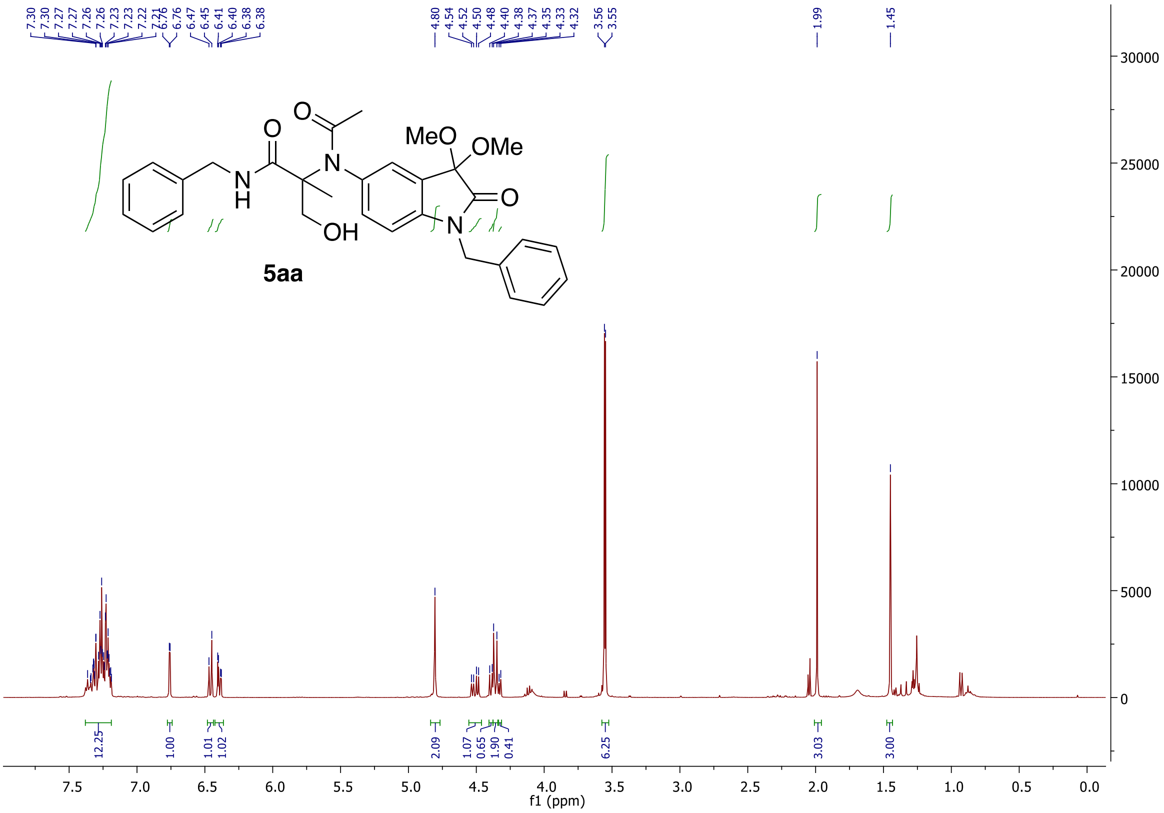

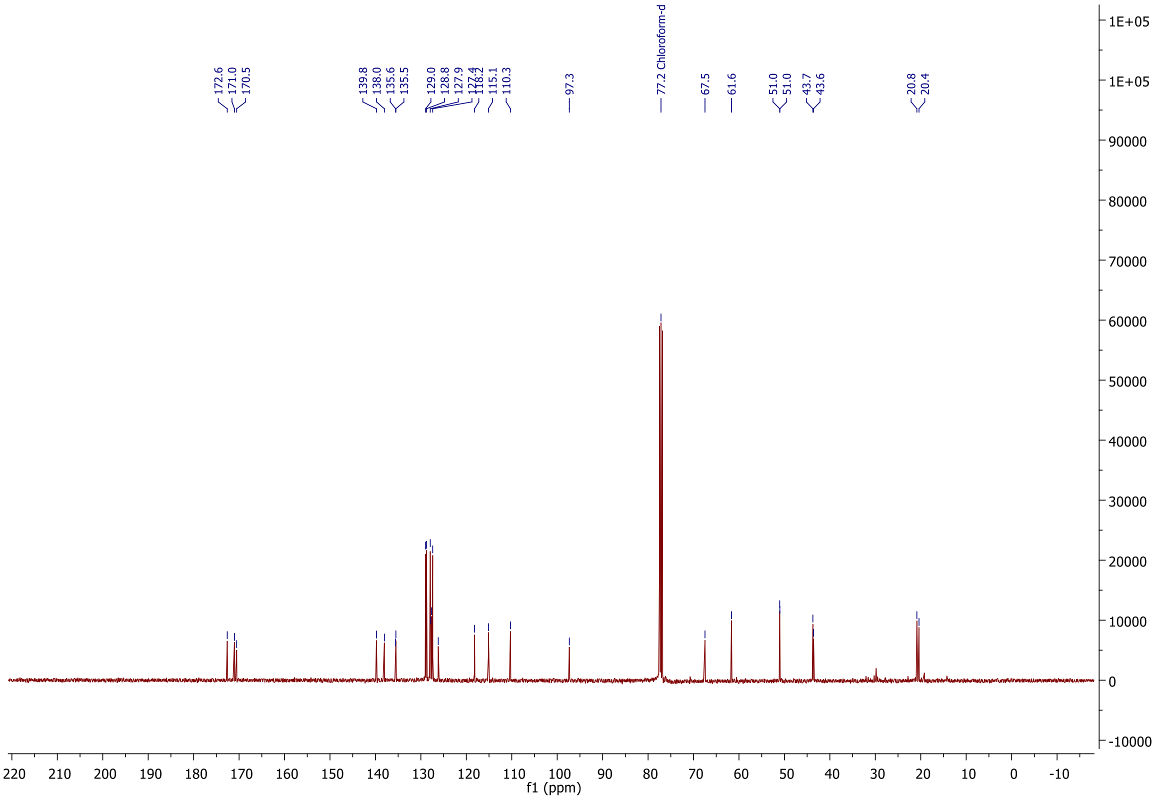

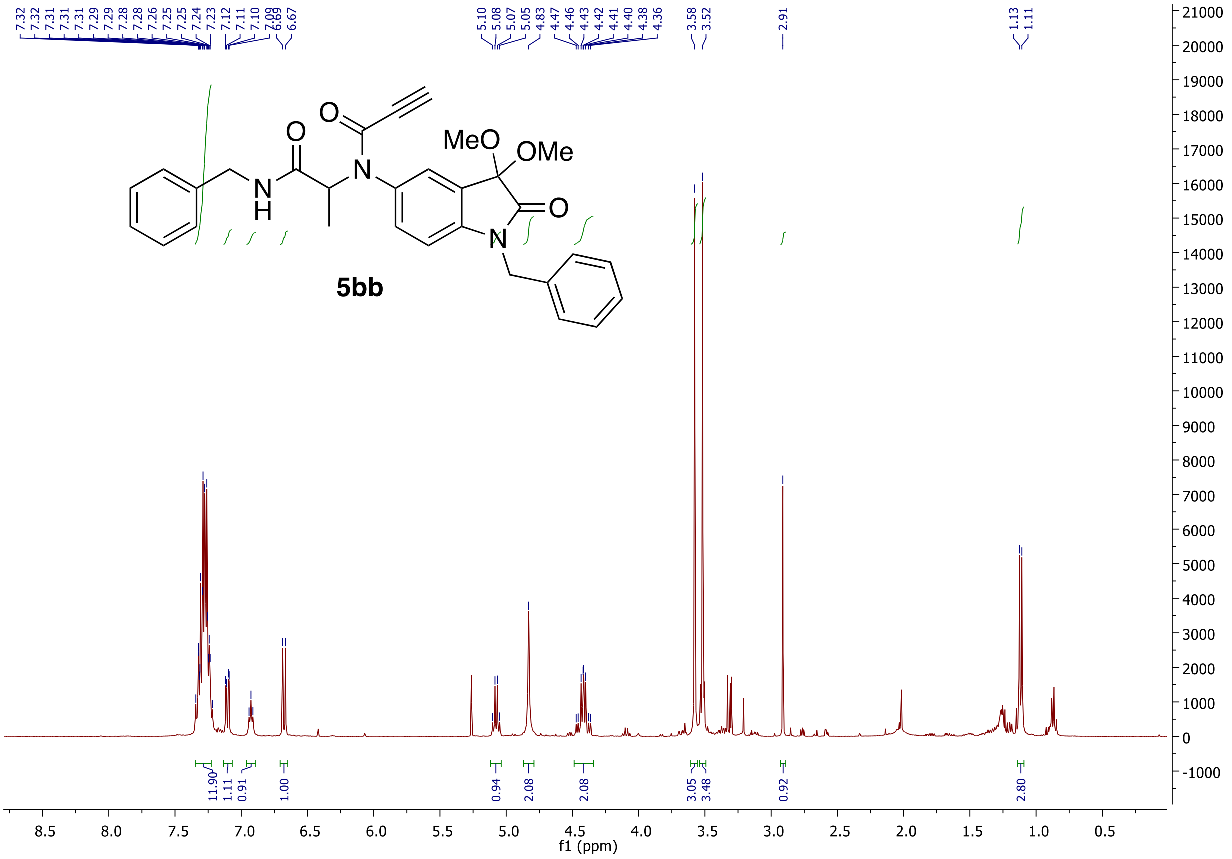
7.32
7.32
7.31
7.31
7.29
7.29
7.28
7.28
7.26
7.25
7.25
7.24
7.23
7.12
7.11
7.10
7.09
6.69
6.67
5.10
5.08
5.07
5.05
4.83
4.47
4.46
4.43
4.42
4.41
4.40
4.38
4.36
3.58
3.52
2.91
1.13
1.11
MeO
OMe
O
N
H
11.90
1.11
0.91
1.00
0.94
2.08
2.08
3.05
3.48
0.92
2.80
f1 (ppm)

5bb

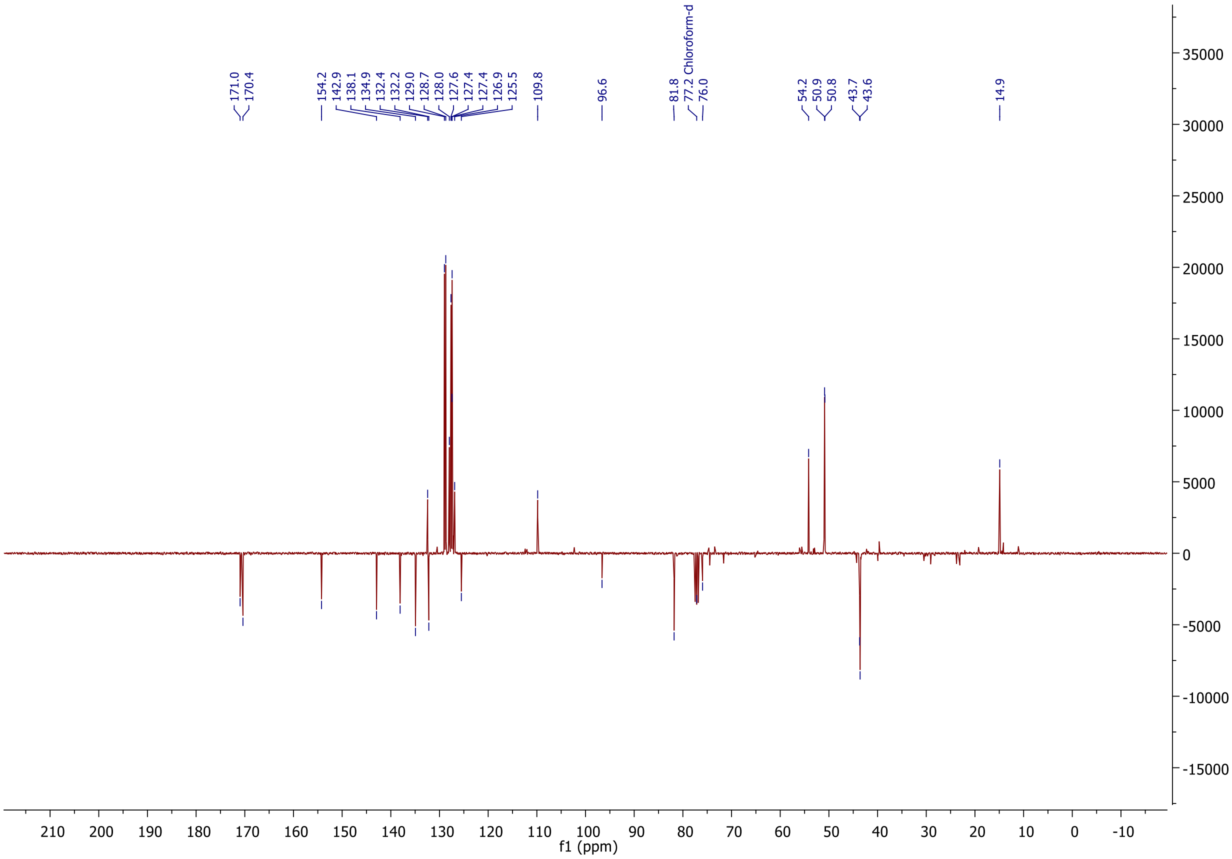
171.0
170.4
154.2
142.9
138.1
134.9
132.4
132.2
129.0
128.7
128.0
127.6
127.4
126.9
125.5
109.8
96.6
81.8
77.2 Chloroform-d
76.0
54.2
50.9
50.8
43.7
43.6
14.9
f1 (ppm)

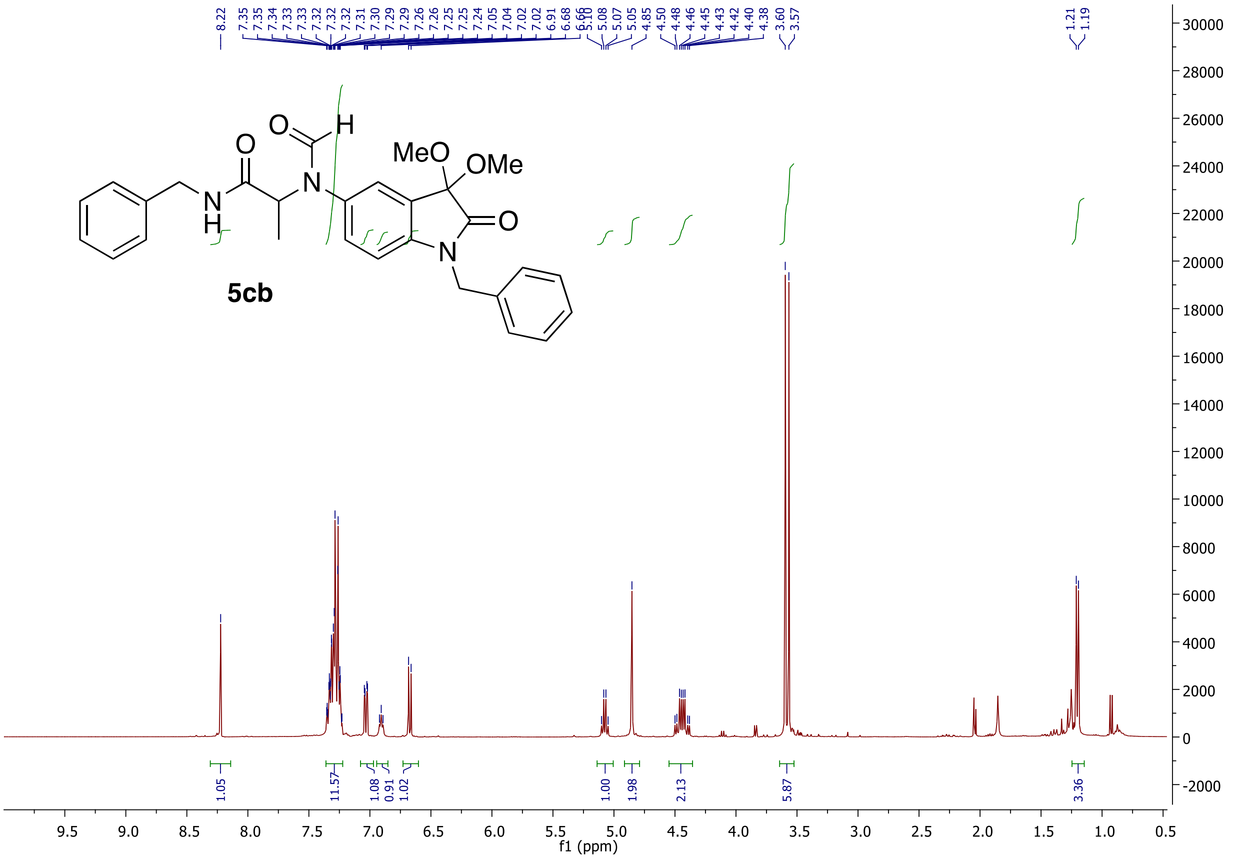
MeO
OMe
O
H
N
5cb
8.22
3.60
3.57
1.21
1.19
1.05
11.57
1.08
0.91
1.02
1.00
1.98
2.13
5.87
3.36
f1 (ppm)

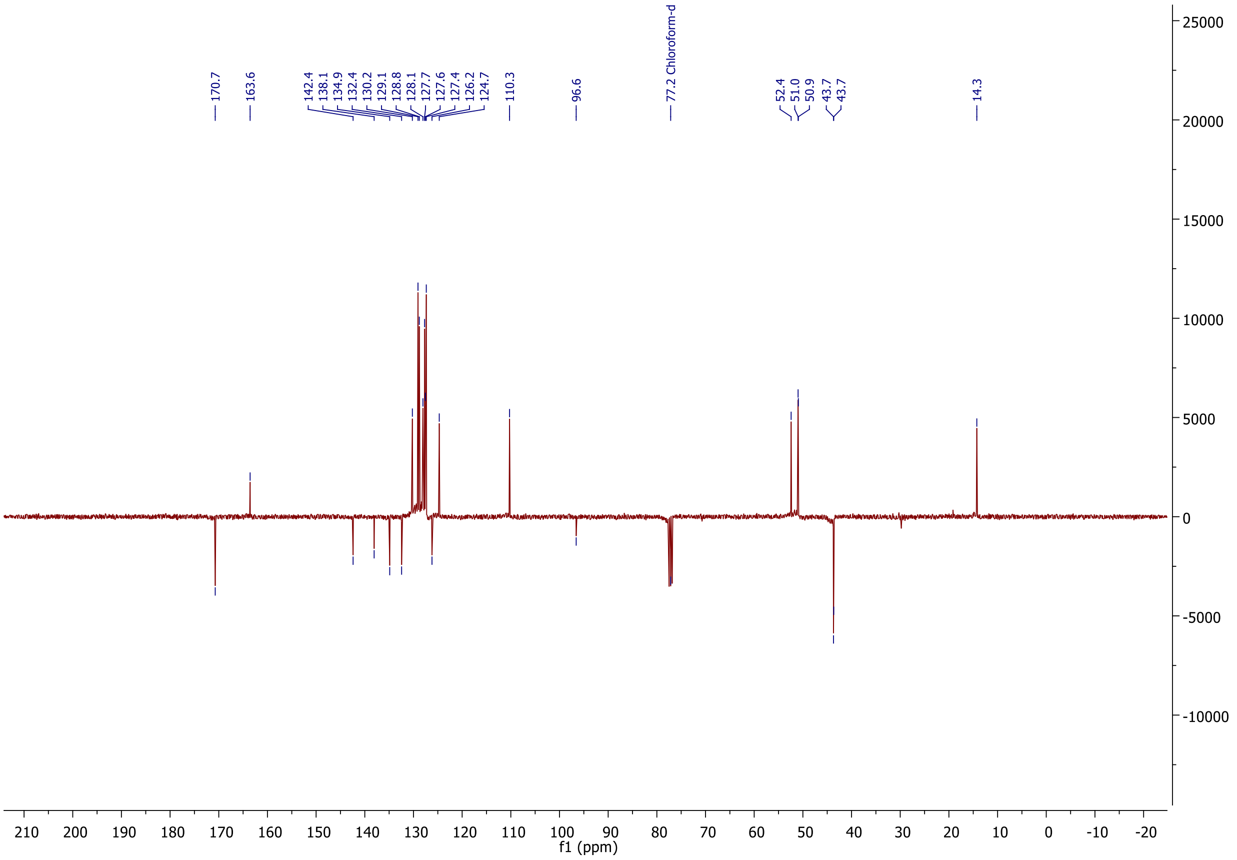
170.7
163.6
142.4
138.1
134.9
132.4
130.2
129.1
128.8
128.1
127.7
127.6
127.4
126.2
124.7
110.3
96.6
77.2 Chloroform-d
52.4
51.0
50.9
43.7
43.7
14.3
f1 (ppm)

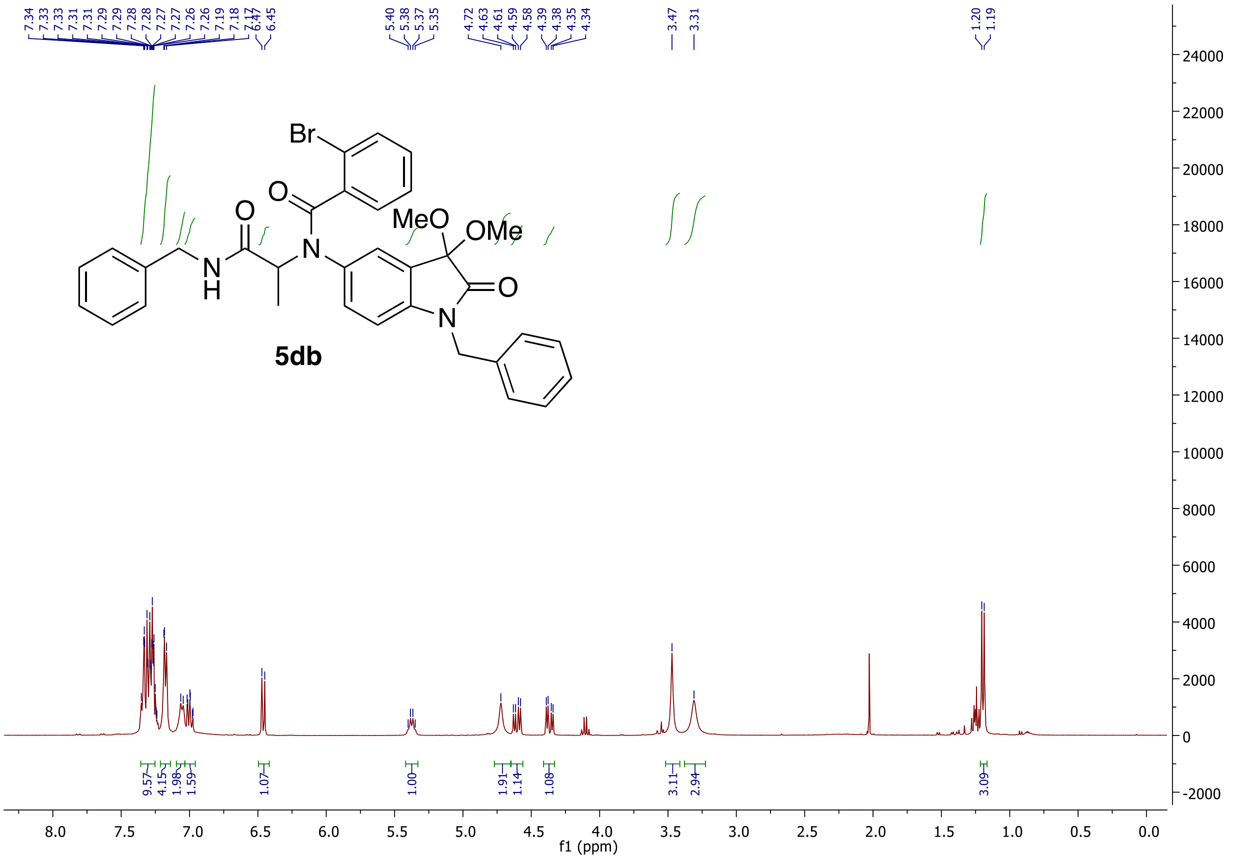
7.34 7.33 7.33 7.31 7.31 7.29 7.29 7.28 7.28 7.27 7.27 7.26 7.26 7.19 7.18 7.17 6.47 6.45
5.40 5.38 5.37 5.35
4.72 4.63 4.61 4.59 4.58 4.39 4.38 4.35 4.34
3.47
3.31
1.20 1.19
Br
O
O
MeO
OMe
N
H
N
O
N
5db
9.57 4.15 1.98 1.59 1.07 1.00 1.91 1.14 1.08 3.11 2.94 3.09
f1 (ppm)

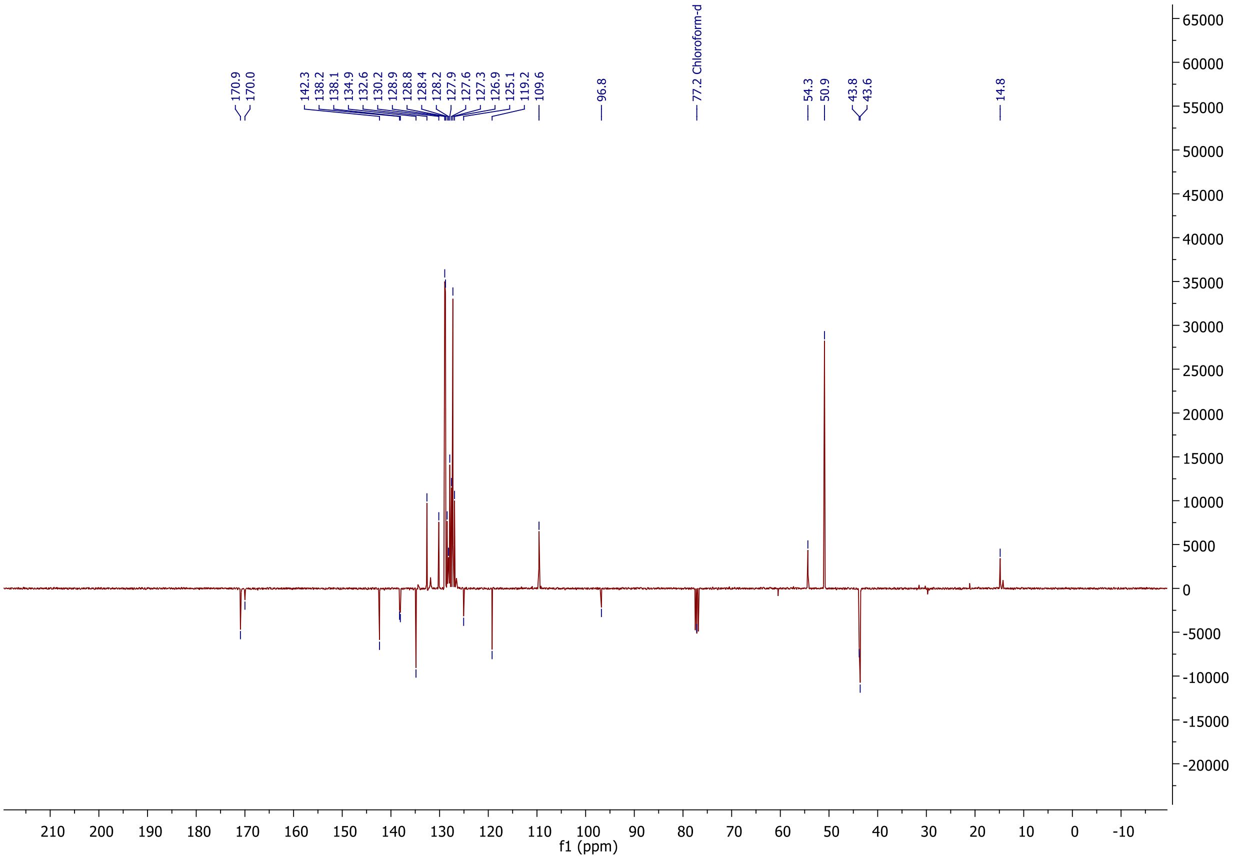
170.9 170.0
142.3 138.2 138.1 134.9 132.6 130.2 128.9 128.8 128.4 128.2 127.9 127.6 127.3 126.9 125.1 119.2 109.6
96.8
77.2 Chloroform-d
54.3 50.9
43.8 43.6
14.8
f1 (ppm)

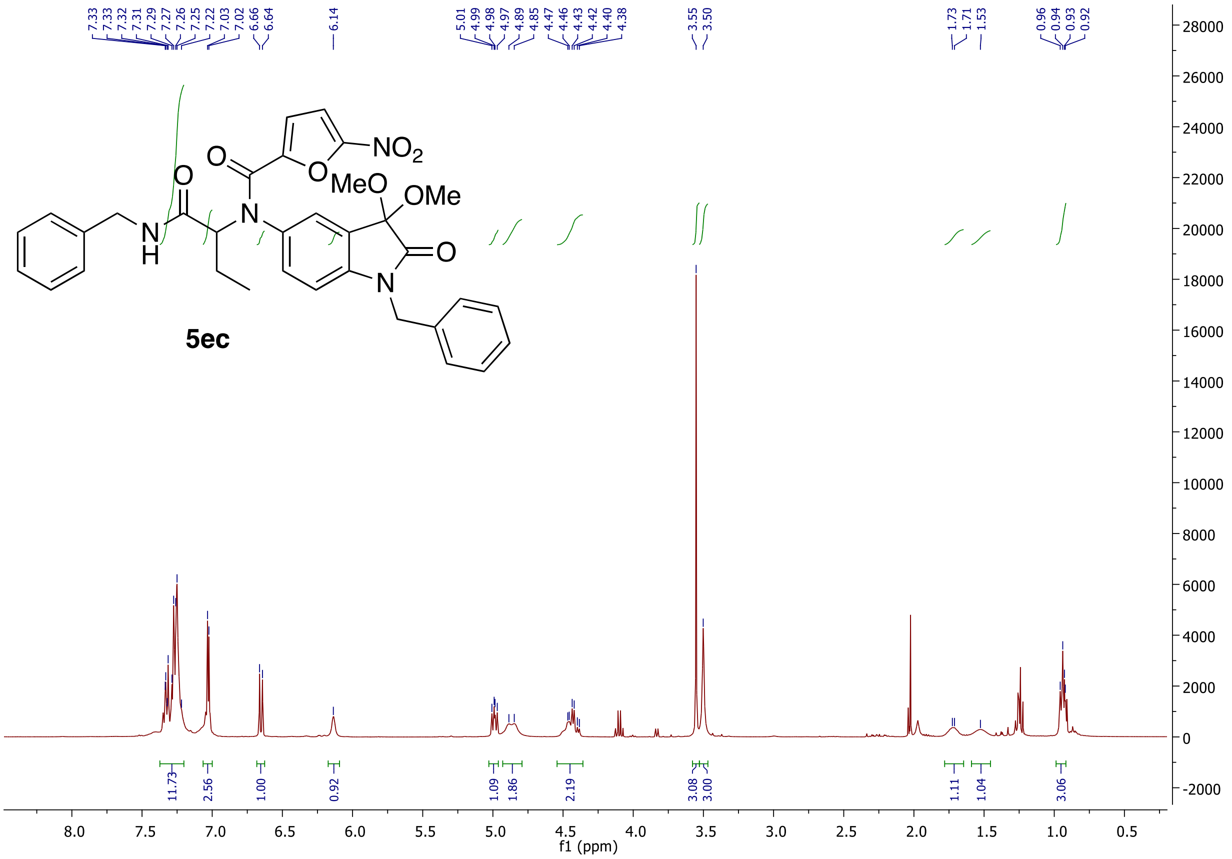
7.33 7.33 7.32 7.31 7.29 7.27 7.26 7.25 7.22 7.03 7.02 6.66 6.64
6.14
5.01 4.99 4.98 4.97 4.89 4.85 4.47 4.46 4.43 4.42 4.40 4.38
3.55 3.50
1.73 1.71 1.53
0.96 0.94 0.93 0.92
NO2
O
MeO
OMe
O
N
N
H
O
O
N
5ec
11.73 2.56 1.00 0.92 1.09 1.86 2.19 3.08 3.00 1.11 1.04 3.06
f1 (ppm)

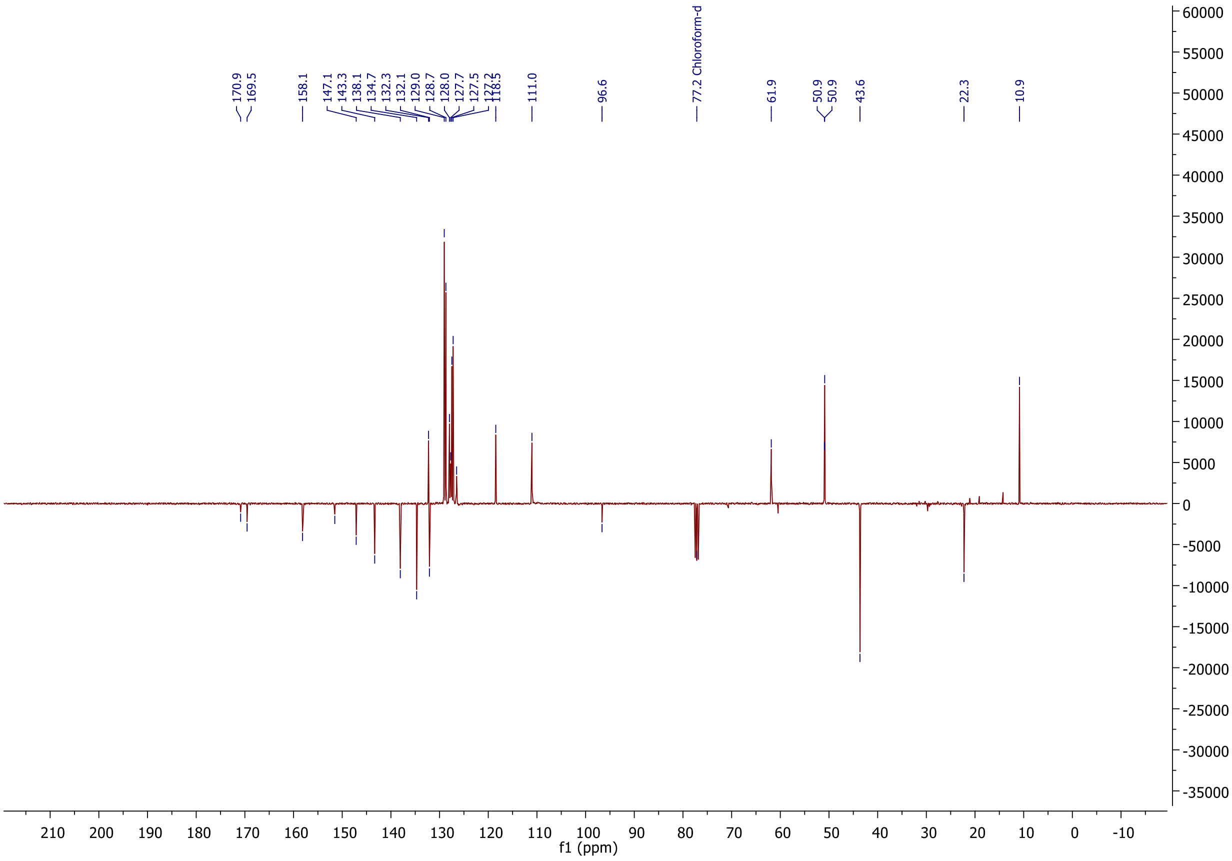
170.9 169.5 158.1 147.1 143.3 138.1 134.7 132.3 132.1 129.0 128.7 128.0 127.7 127.5 127.2 118.5 111.0 96.6
77.2 Chloroform-d
61.9 50.9 50.9 43.6 22.3 10.9
f1 (ppm)

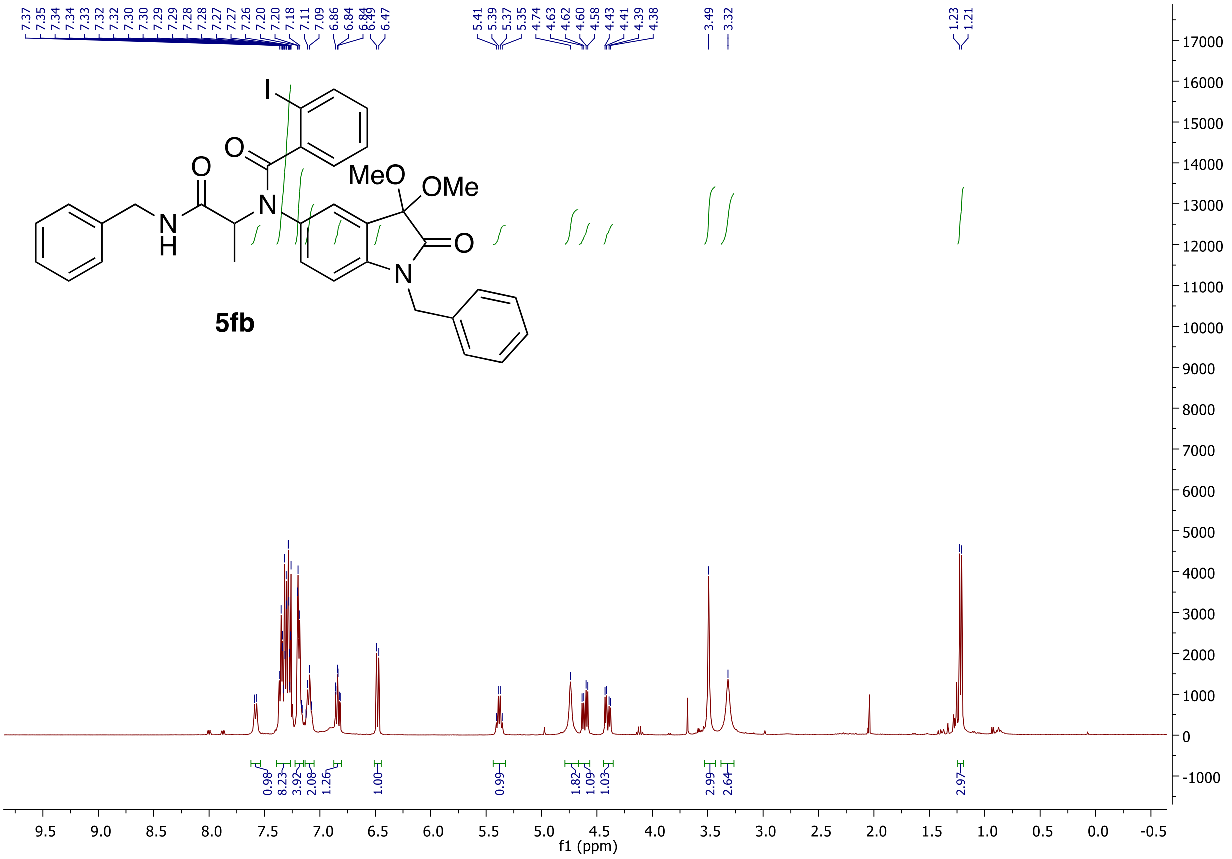
5fb
MeO
OMe
f1 (ppm)
77.2 Chloroform-d

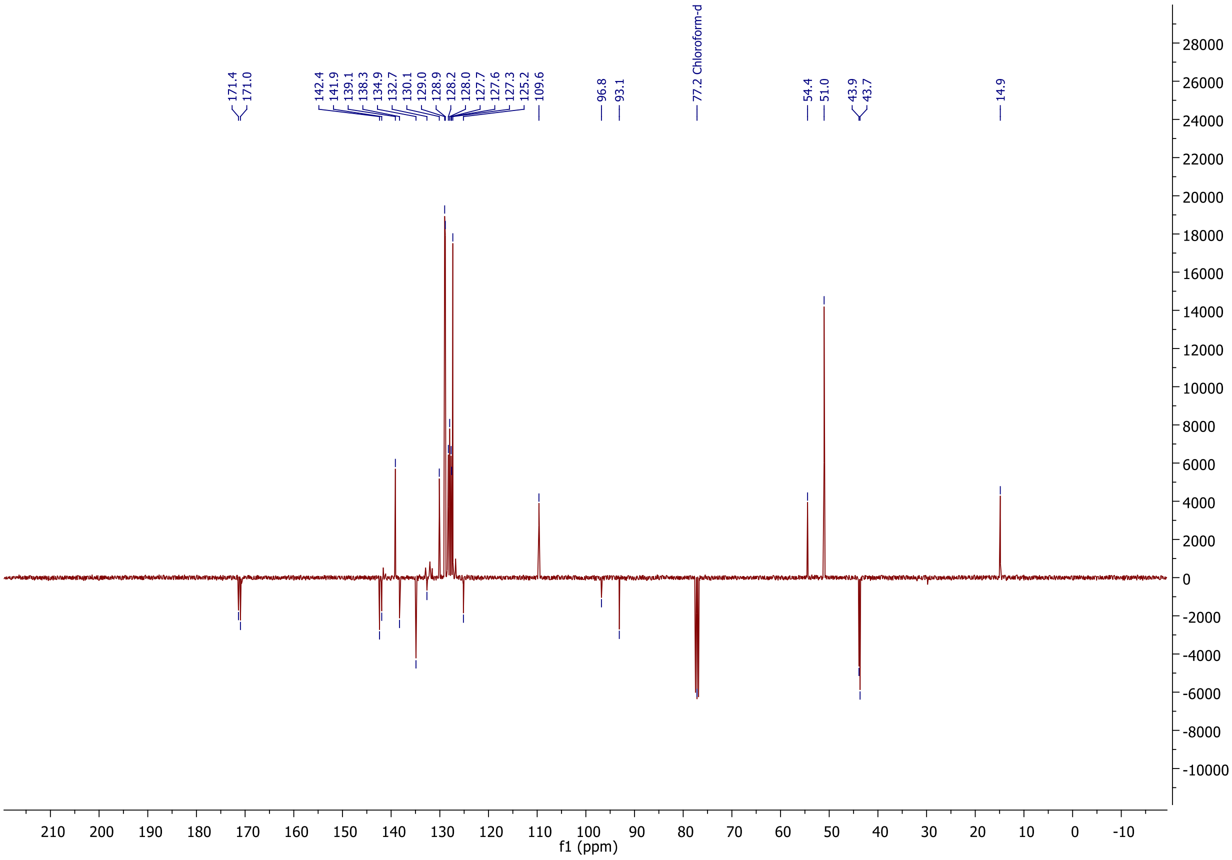
f1 (ppm)
77.2 Chloroform-d

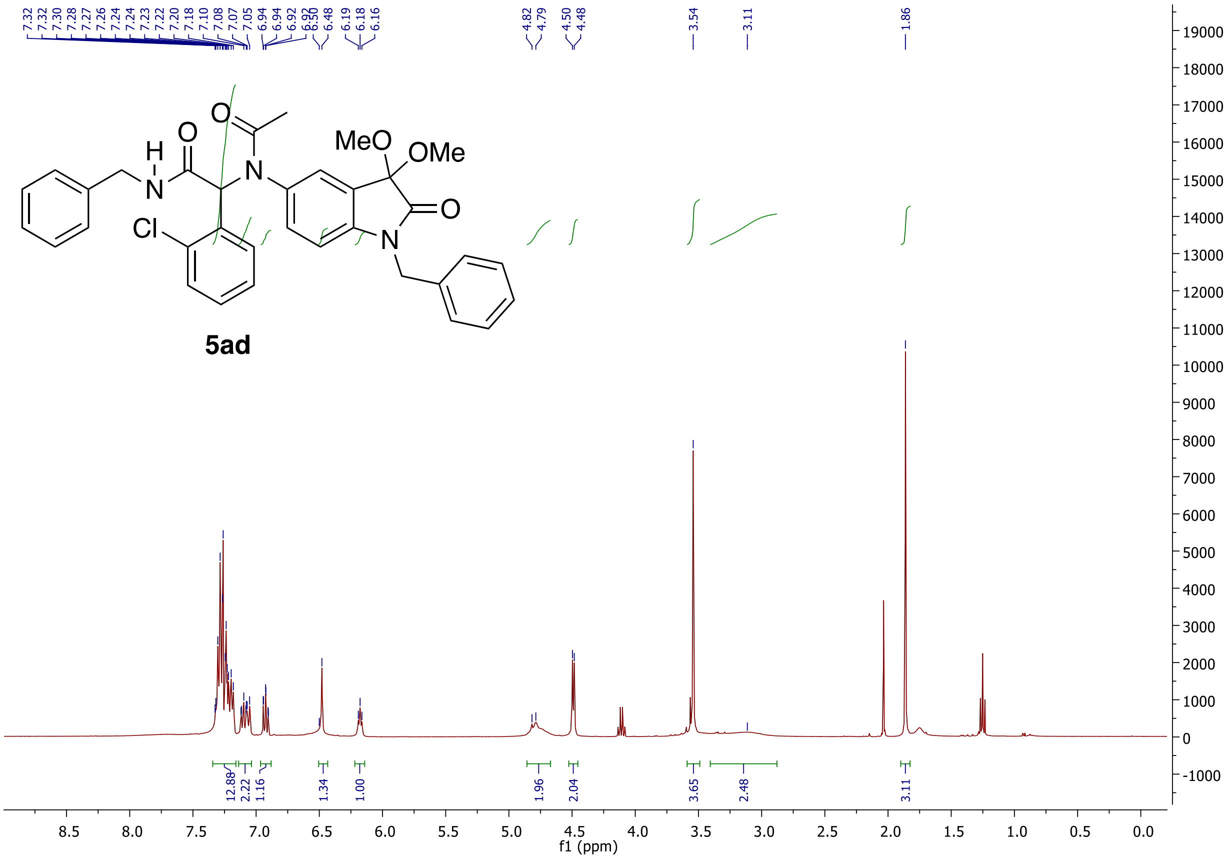
7.32 7.32 7.30 7.28 7.27 7.26 7.24 7.24 7.23 7.22 7.20 7.18 7.10 7.08 7.07 7.05 6.94 6.94 6.92 6.92 6.50 6.48 6.19 6.18 6.16
4.82 4.79 4.50 4.48
3.54
3.11
1.86
MeO
OMe
Cl
5ad
12.88 2.22 1.16 1.34 1.00 1.96 2.04 3.65 2.48 3.11
f1 (ppm)

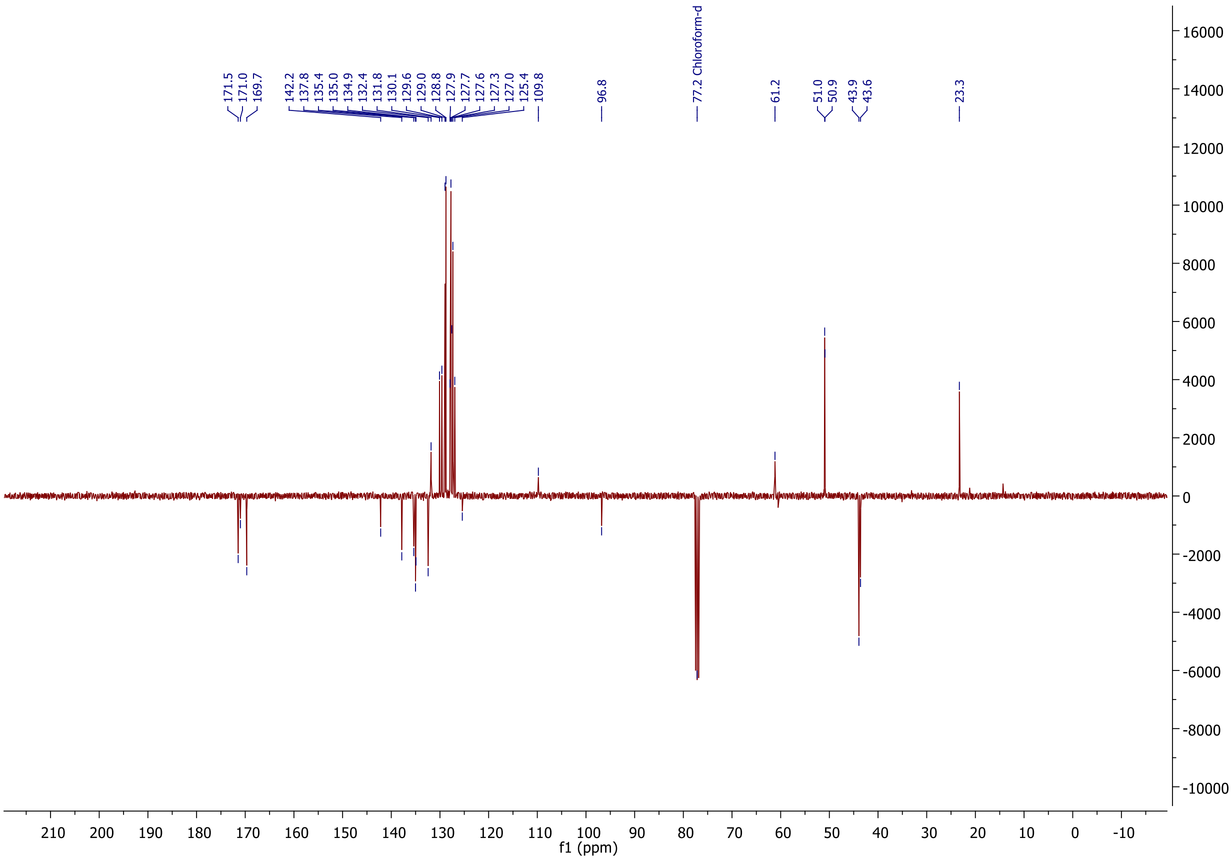
171.5 171.0 169.7
142.2 137.8 135.4 135.0 134.9 132.4 131.8 130.1 129.6 129.0 128.8 127.9 127.7 127.6 127.3 127.0 125.4 109.8
96.8
77.2 Chloroform-d
61.2
51.0 50.9
43.9 43.6
23.3
f1 (ppm)

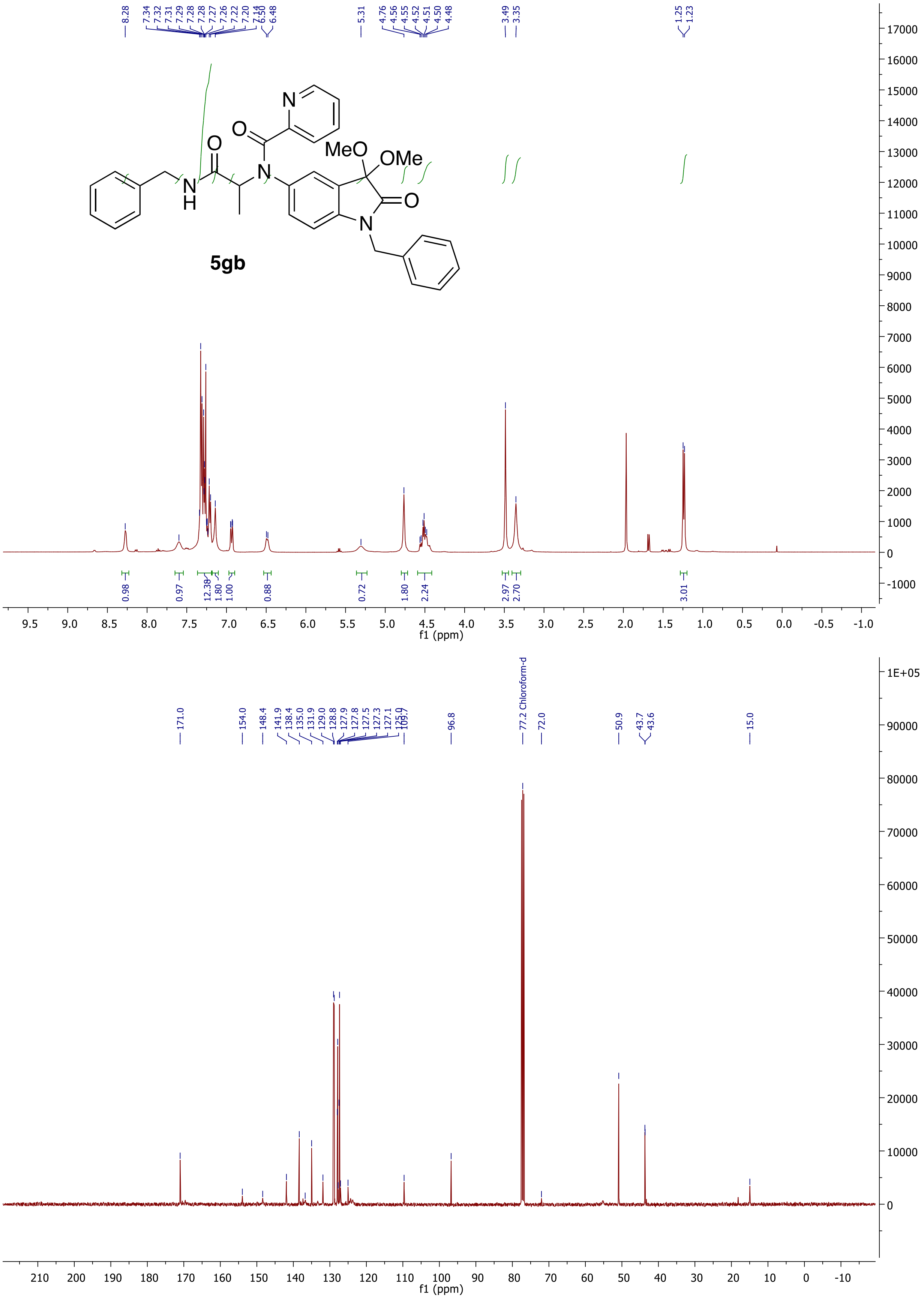
8.28
7.34
7.32
7.31
7.29
7.28
7.28
7.27
7.26
7.22
7.20
7.14
6.50
6.48
5.31
4.76
4.56
4.55
4.52
4.51
4.50
4.48
3.49
3.35
1.25
1.23
N
O
O
MeO
OMe
N
H
N
N
O
0.98
0.97
12.38
1.80
1.00
0.88
0.72
1.80
2.24
2.97
2.70
3.01
f1 (ppm)
171.0
154.0
148.4
141.9
138.4
135.0
131.9
129.0
128.8
127.9
127.8
127.5
127.3
127.1
125.0
109.7
96.8
77.2 Chloroform-d
72.0
50.9
43.7
43.6
15.0
f1 (ppm)

5gb

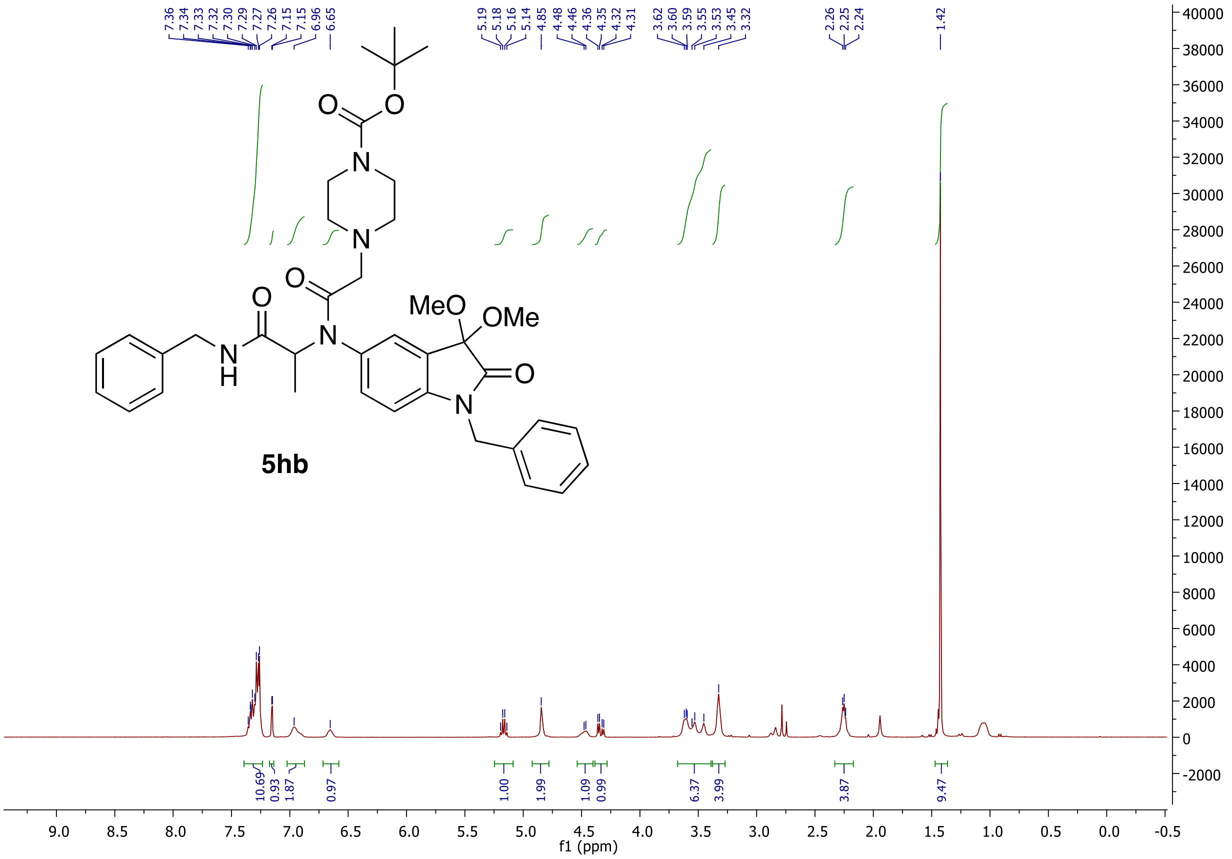
5hb
f1 (ppm)

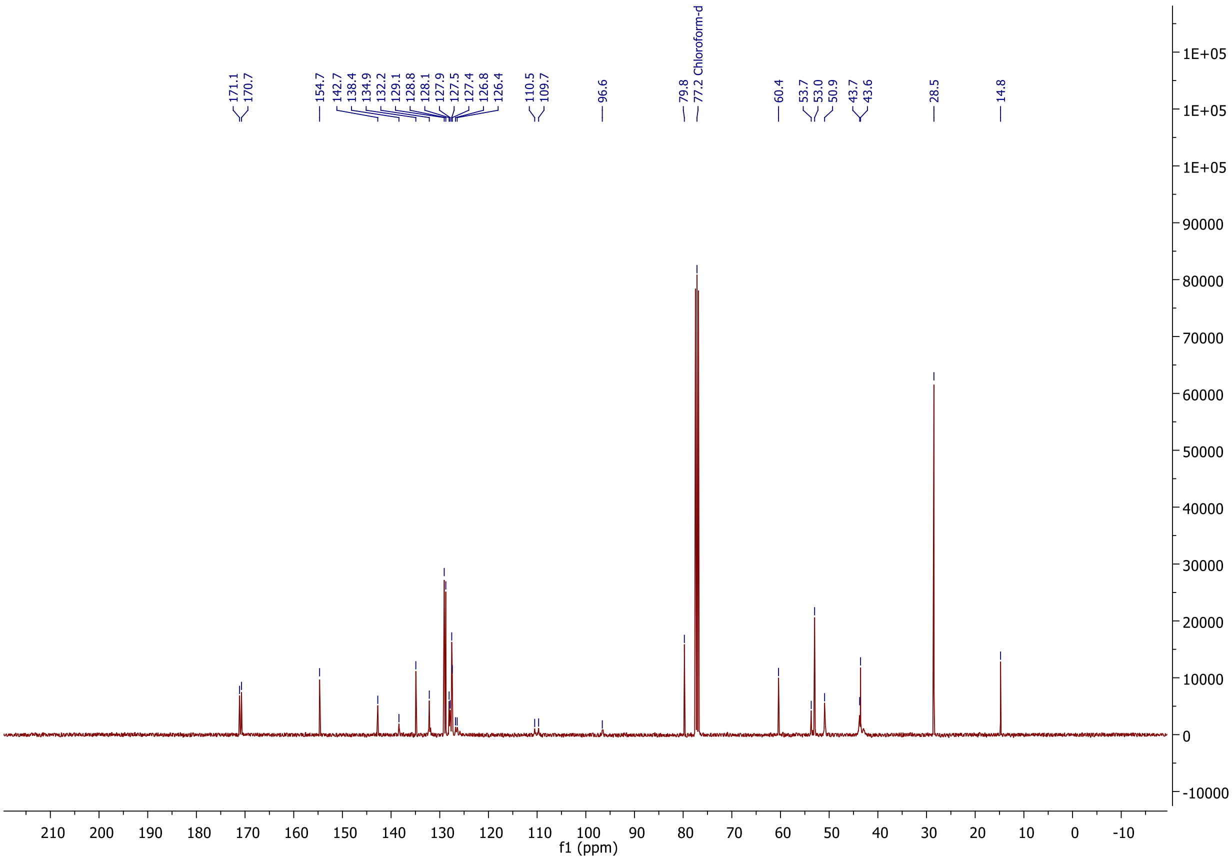
Chloroform-d
f1 (ppm)

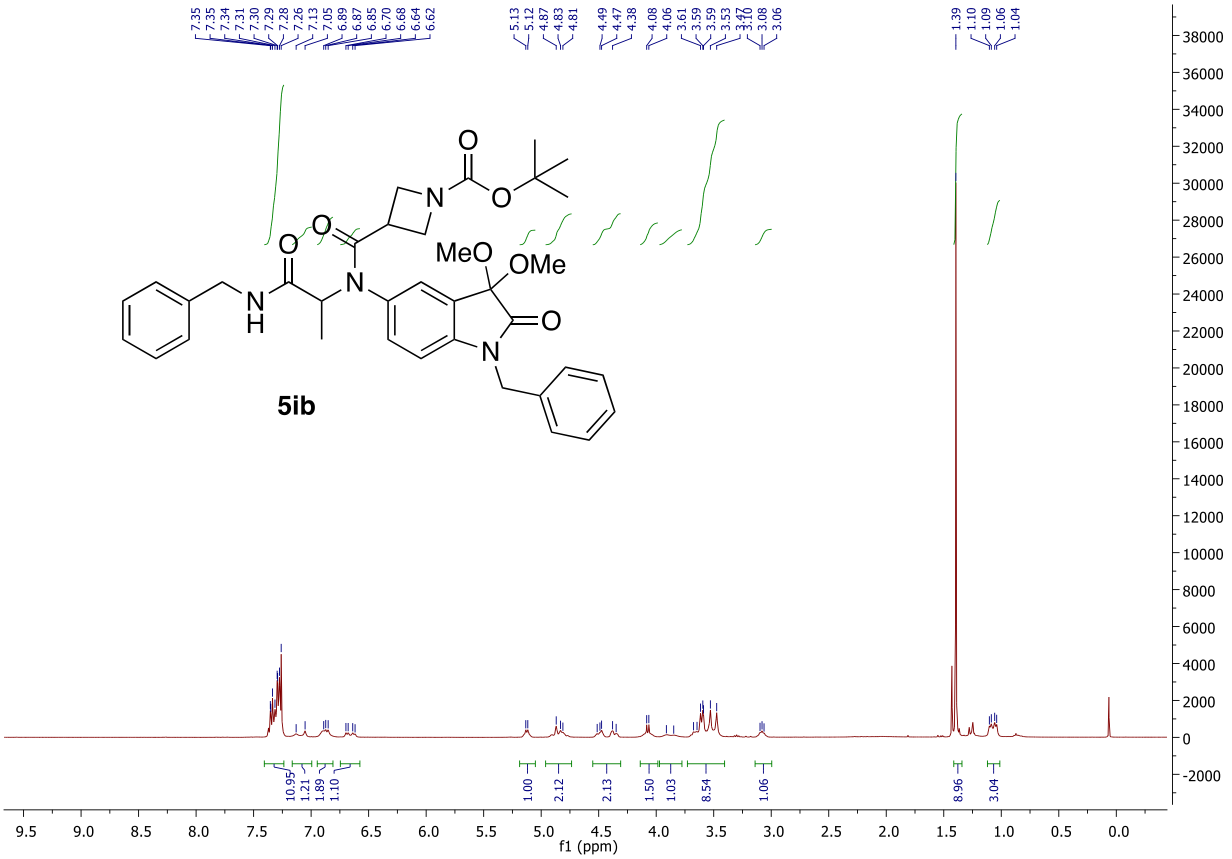
7.35 7.35 7.34 7.31 7.30 7.29 7.28 7.26 7.13 7.05 6.89 6.87 6.85 6.70 6.68 6.64 6.62
5.13 5.12 4.87 4.83 4.81
4.49 4.47 4.38 4.08 4.06 3.61 3.59 3.59 3.53 3.47 3.10 3.08 3.06
1.39 1.10 1.09 1.06 1.04
MeO
OMe
5ib
10.95 1.21 1.89 1.10 1.00 2.12 2.13 1.50 1.03 8.54 1.06 8.96 3.04
f1 (ppm)

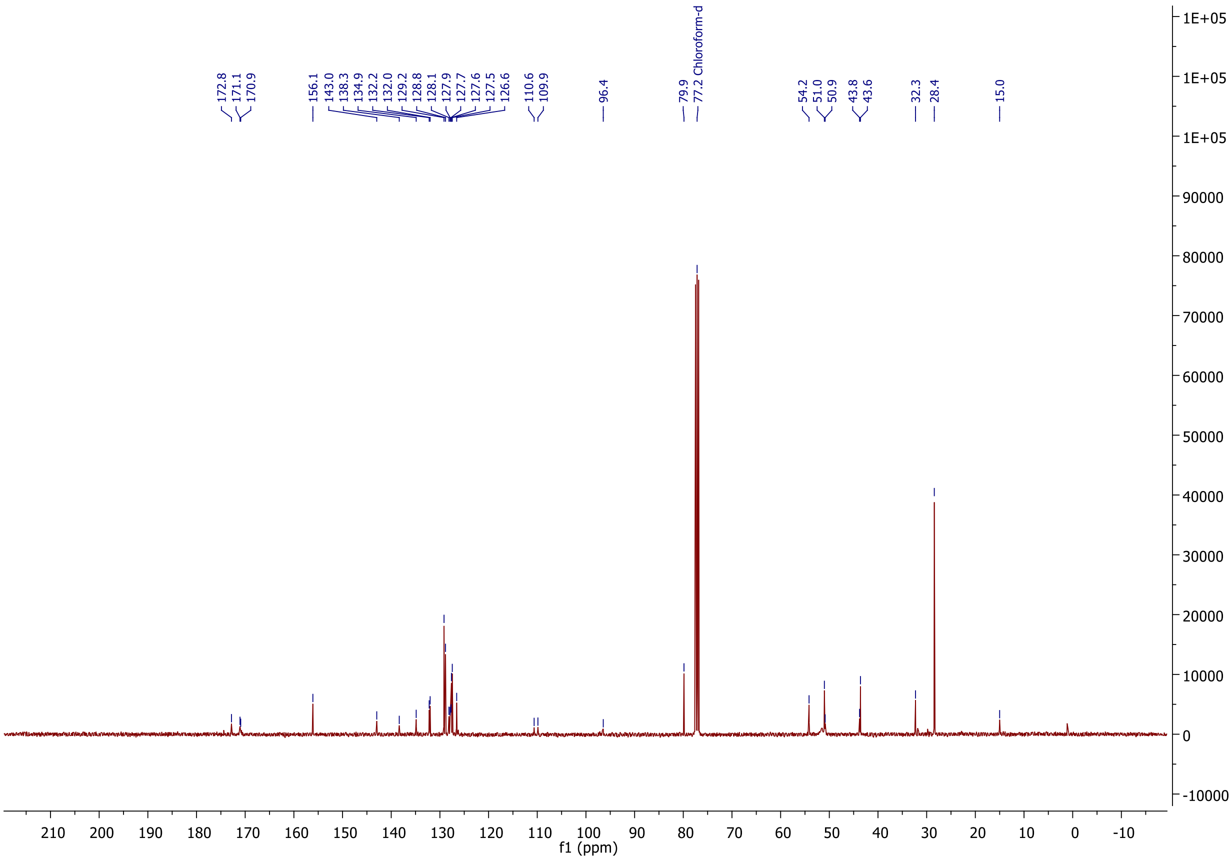
172.8 171.1 170.9
156.1 143.0 138.3 134.9 132.2 132.0 129.2 128.8 128.1 127.9 127.7 127.6 127.5 126.6
110.6 109.9
96.4
79.9
77.2 Chloroform-d
54.2 51.0 50.9 43.8 43.6 32.3 28.4 15.0
f1 (ppm)

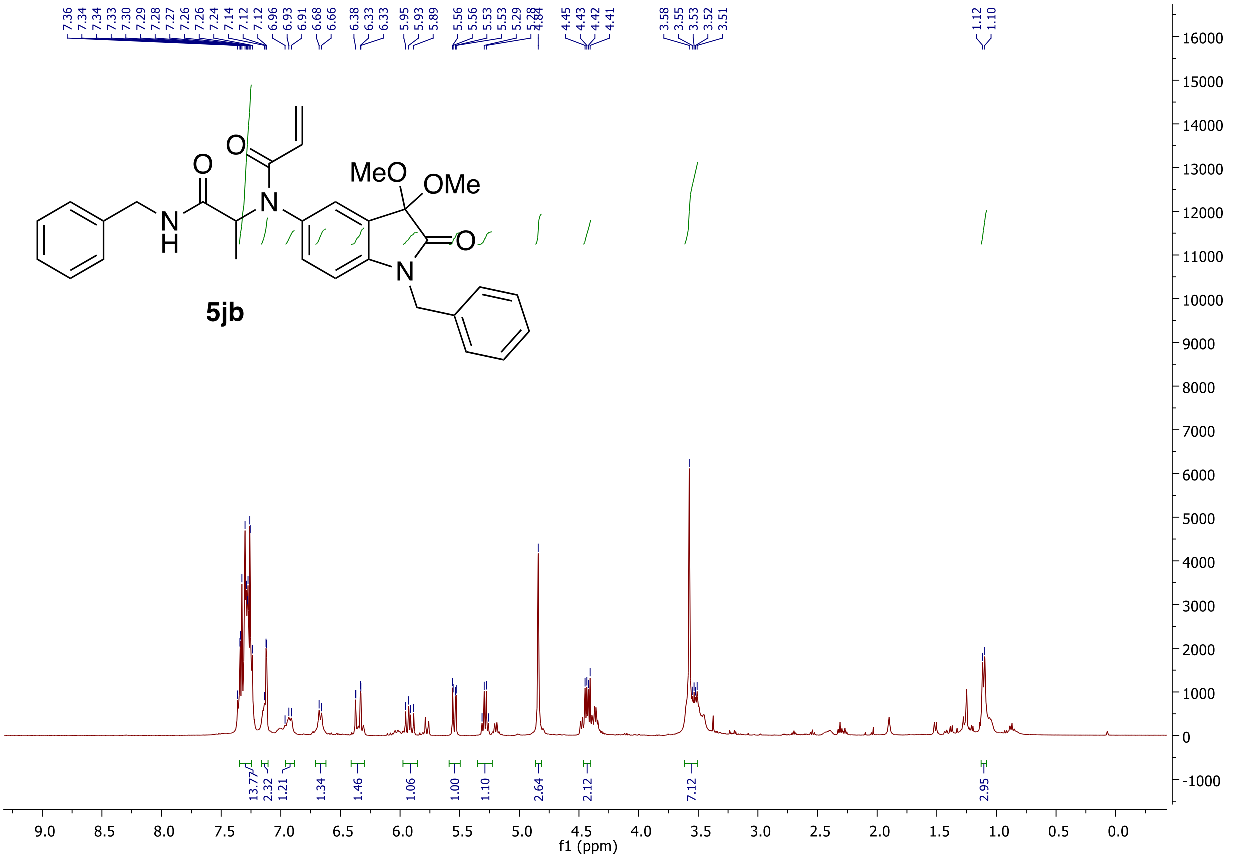
5jb
MeO
OMe
f1 (ppm)

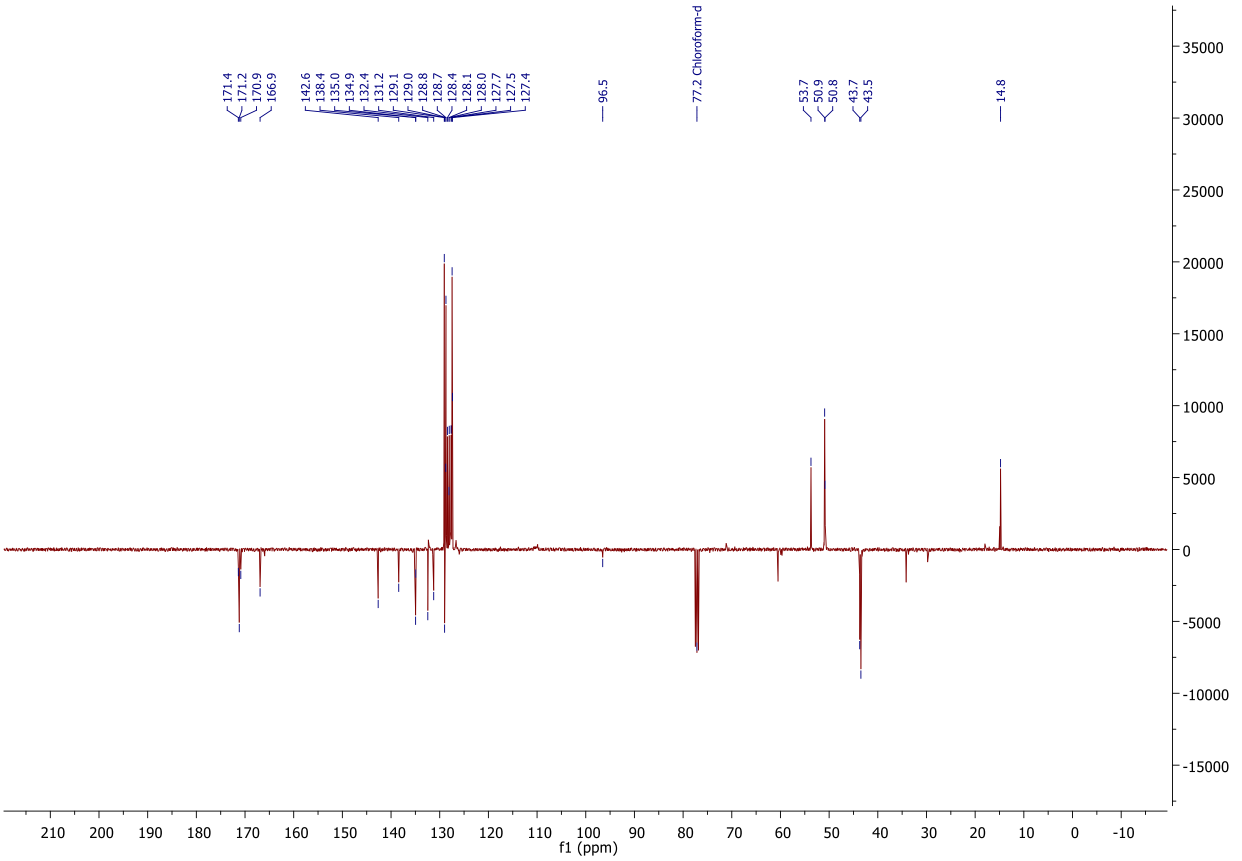
77.2 Chloroform-d
f1 (ppm)

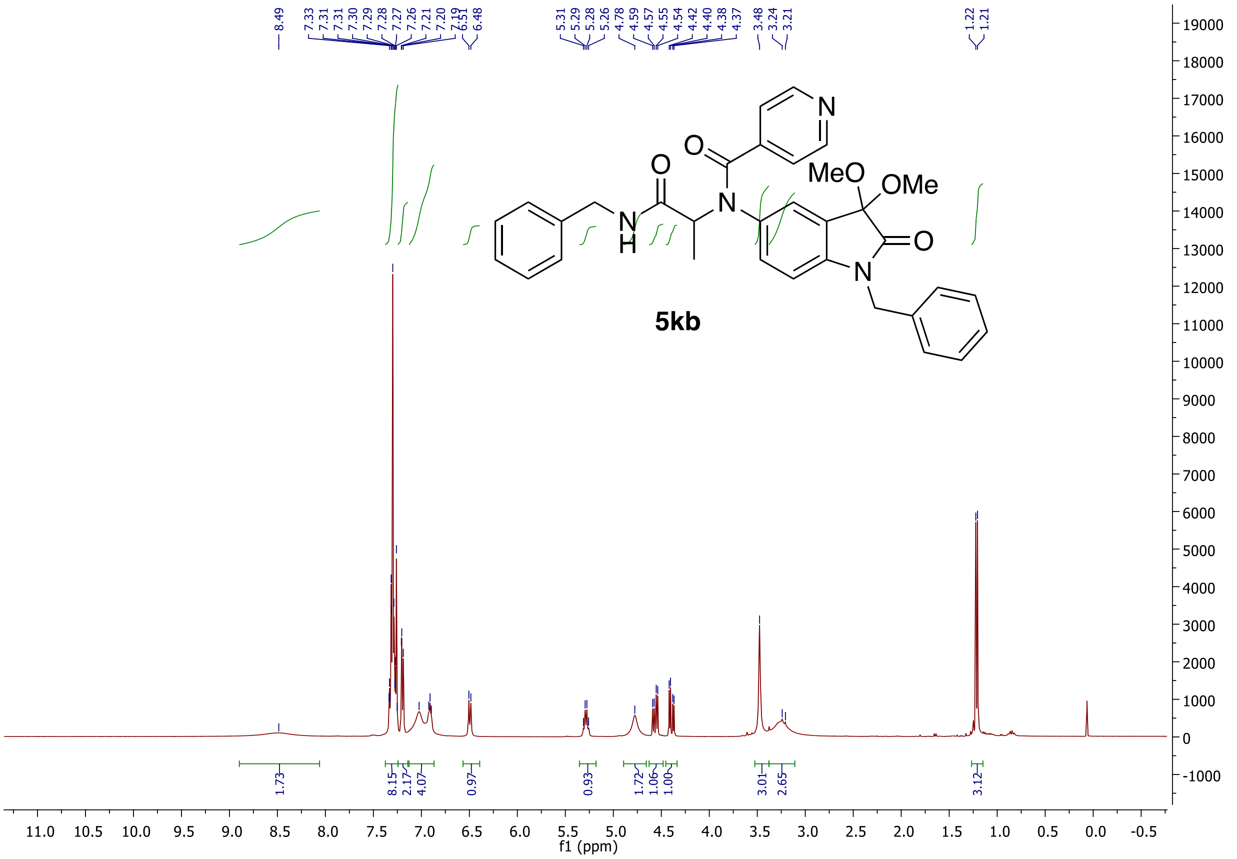
8.49
7.33 7.31 7.31 7.30 7.29 7.28 7.27 7.26 7.21 7.20 7.19 6.51 6.48
5.31 5.29 5.28 5.26 4.78 4.59 4.57 4.55 4.54 4.42 4.40 4.38 4.37 3.48 3.24 3.21
1.22 1.21
MeO
OMe
5kb
1.73 8.15 2.17 4.07 0.97 0.93 1.72 1.06 1.00 3.01 2.65 3.12
f1 (ppm)

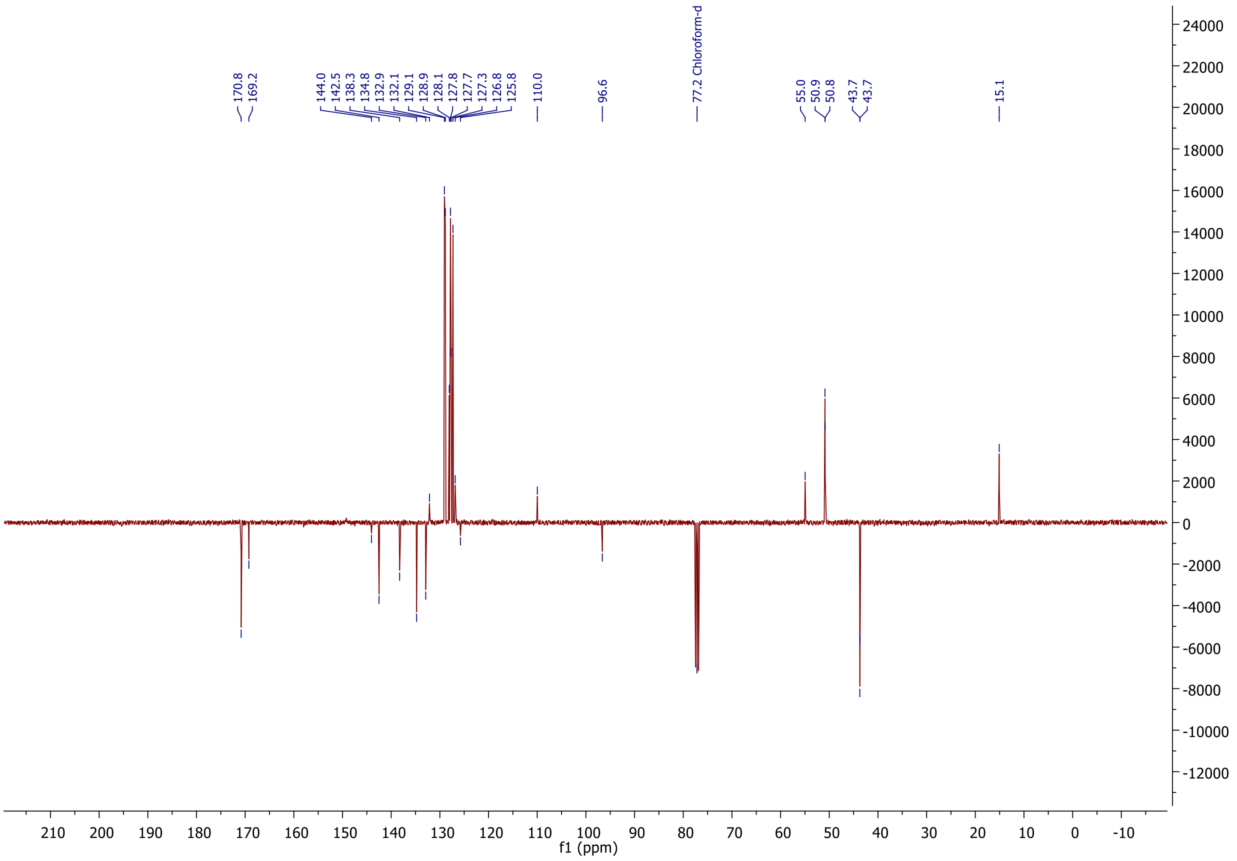
170.8 169.2
144.0 142.5 138.3 134.8 132.9 132.1 129.1 128.9 128.1 127.8 127.7 127.3 126.8 125.8
110.0
96.6
77.2 Chloroform-d
55.0 50.9 50.8 43.7 43.7
15.1
f1 (ppm)

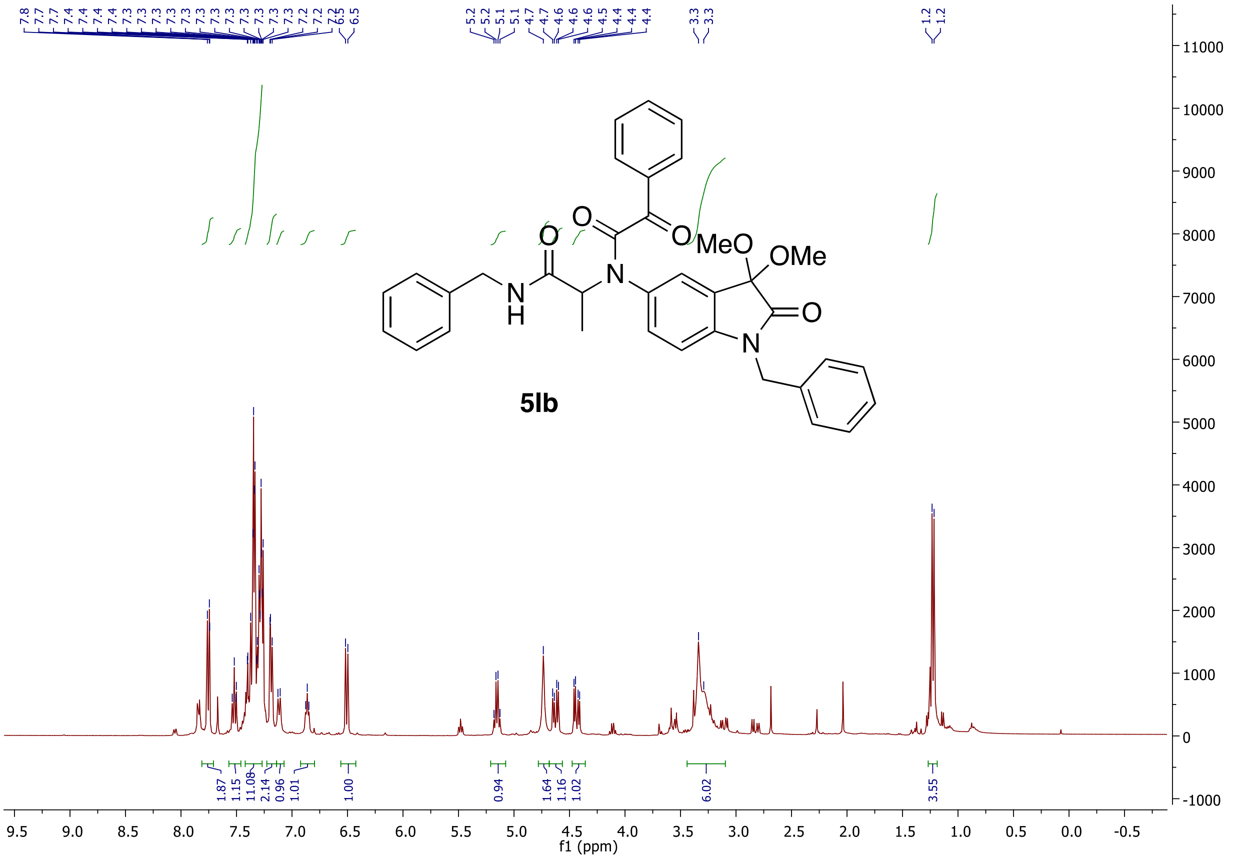
7.8 7.7 7.7 7.4 7.4 7.4 7.3 7.3 7.3 7.3 7.3 7.3 7.3 7.3 7.3 7.3 7.3 7.2 7.2 7.2 6.5 6.5
5.2 5.2 5.1 5.1 4.7 4.7 4.6 4.6 4.6 4.5 4.4 4.4 4.4
3.3 3.3
1.2 1.2
O
O
MeO
OMe
O
N
N
H
N
O
5lb
1.87 1.15 11.08 2.14 0.96 1.01 1.00 0.94 1.64 1.16 1.02 6.02 3.55
f1 (ppm)

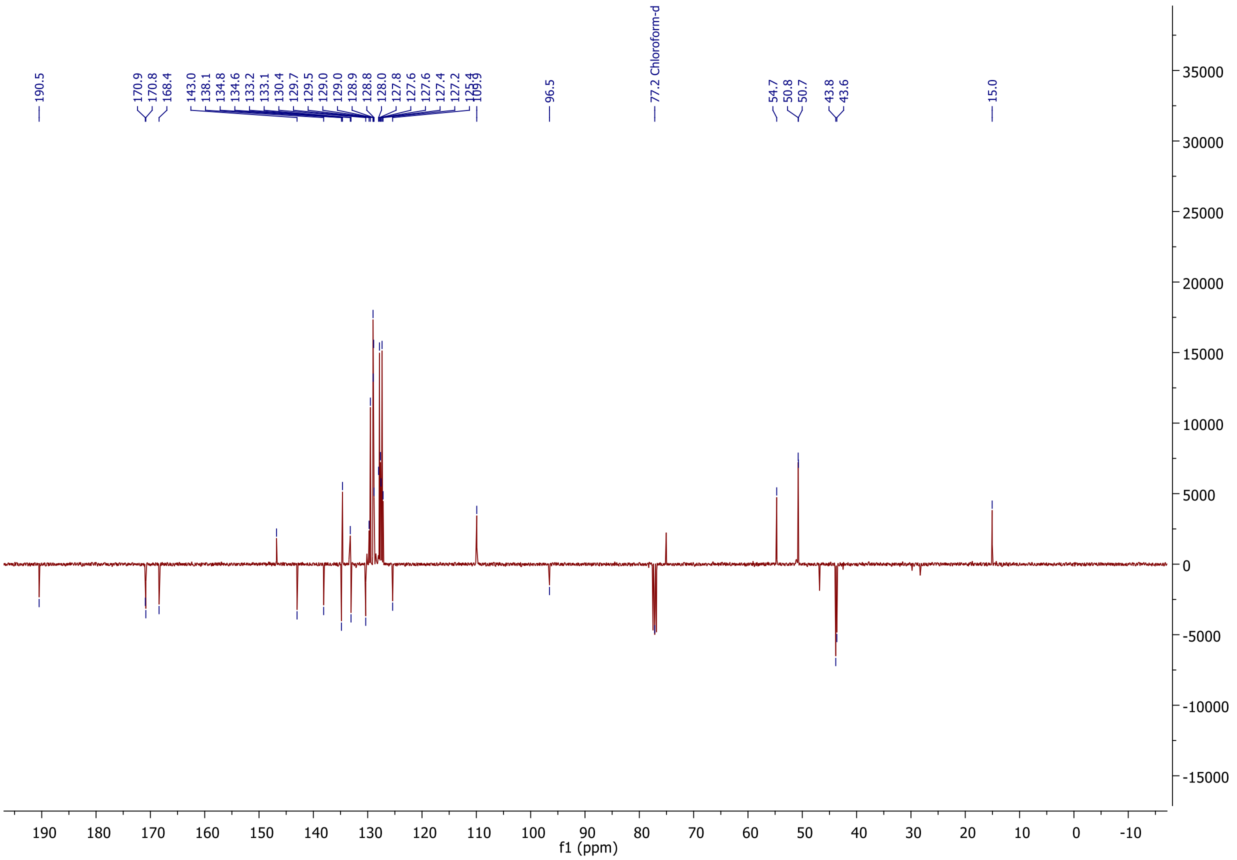
190.5
170.9 170.8 168.4
143.0 138.1 134.8 134.6 133.2 133.1 130.4 129.7 129.5 129.0 129.0 128.9 128.8 128.0 127.8 127.6 127.4 127.2 125.4 109.9
96.5
77.2 Chloroform-d
54.7 50.8 50.7 43.8 43.6
15.0
f1 (ppm)

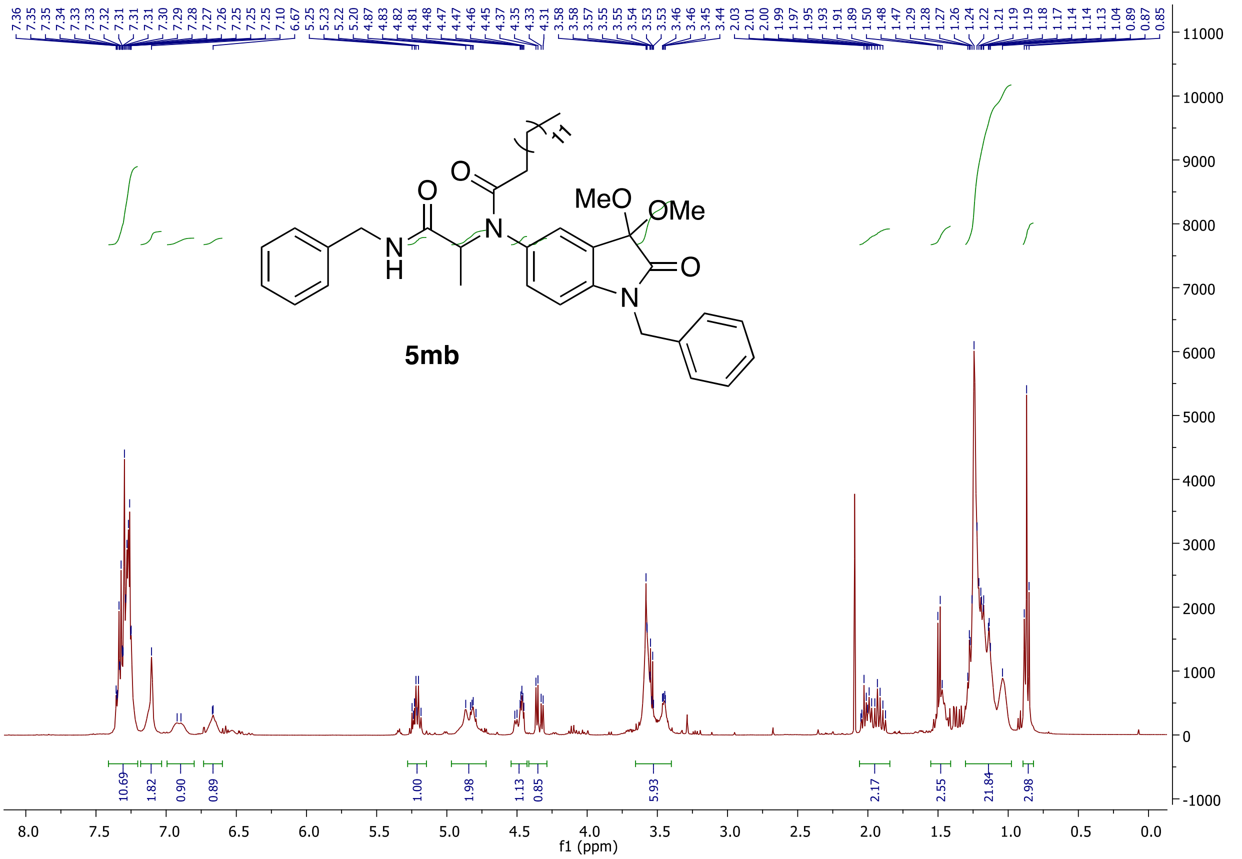
7.36 7.35 7.35 7.34 7.33 7.33 7.32 7.31 7.31 7.31 7.30 7.29 7.28 7.27 7.26 7.25 7.25 7.25 7.10 6.67 5.25 5.23 5.22 5.20 4.87 4.83 4.82 4.81 4.48 4.47 4.47 4.46 4.45 4.37 4.35 4.33 4.31 3.58 3.58 3.57 3.55 3.55 3.54 3.53 3.53 3.46 3.45 3.44 2.03 2.01 2.00 1.99 1.97 1.95 1.93 1.91 1.89 1.50 1.48 1.47 1.29 1.28 1.27 1.26 1.24 1.22 1.21 1.19 1.19 1.18 1.17 1.14 1.14 1.13 1.04 0.89 0.87 0.85
MeO
OMe
O
N
H
11
5mb
10.69 1.82 0.90 0.89 1.00 1.98 1.13 0.85 5.93 2.17 2.55 21.84 2.98
f1 (ppm)

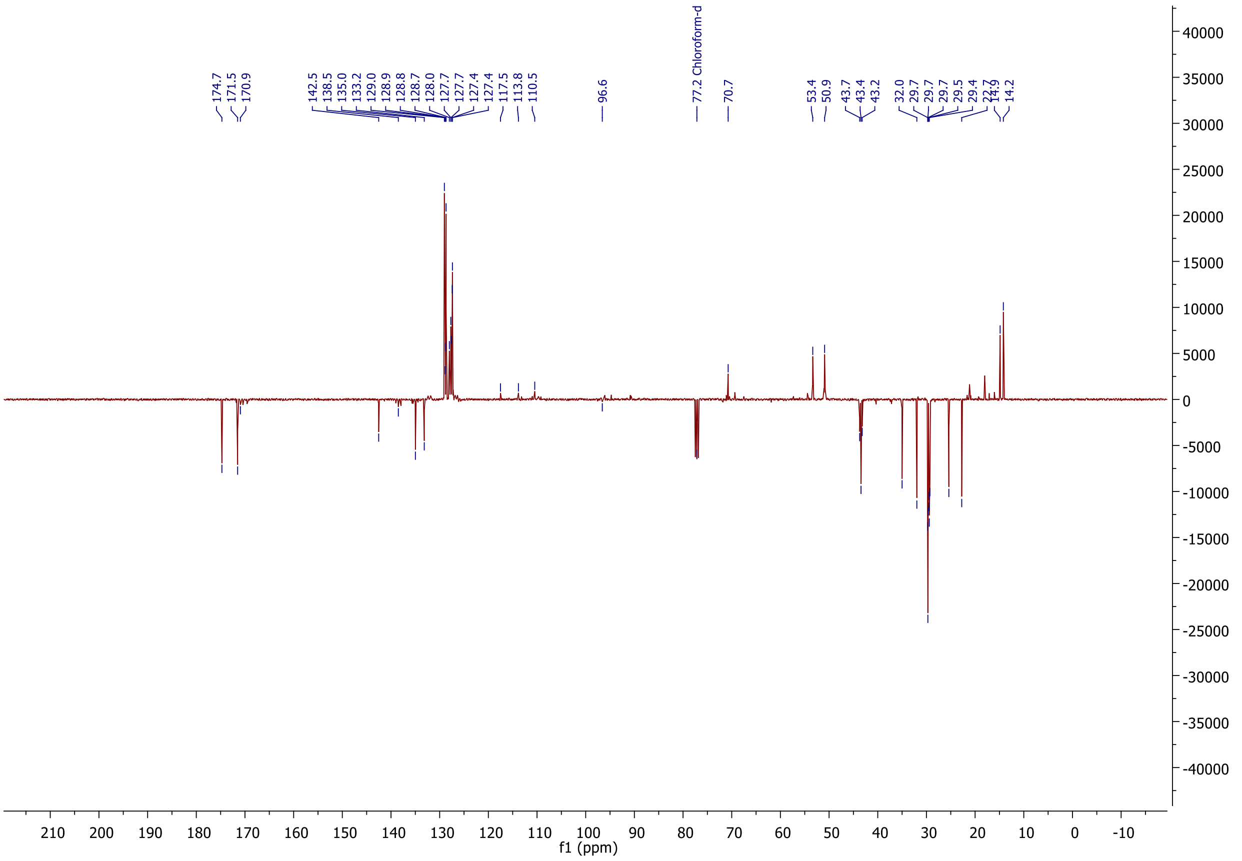
174.7 171.5 170.9 142.5 138.5 135.0 133.2 129.0 128.9 128.8 128.7 128.0 127.7 127.4 127.4 117.5 113.8 110.5 96.6 77.2 Chloroform-d 70.7 53.4 50.9 43.7 43.4 43.2 32.0 29.7 29.7 29.7 29.5 29.4 22.7 14.3 14.2
f1 (ppm)

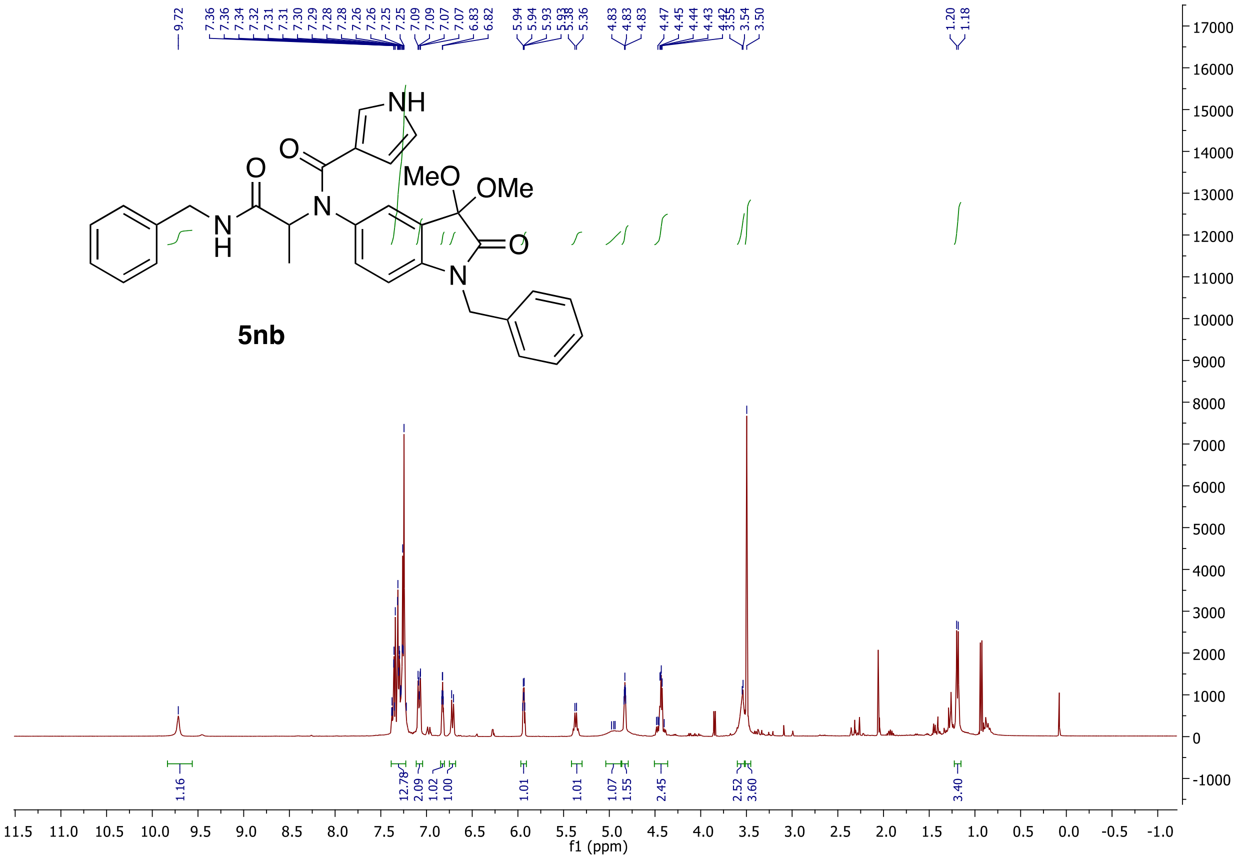
5nb
f1 (ppm)

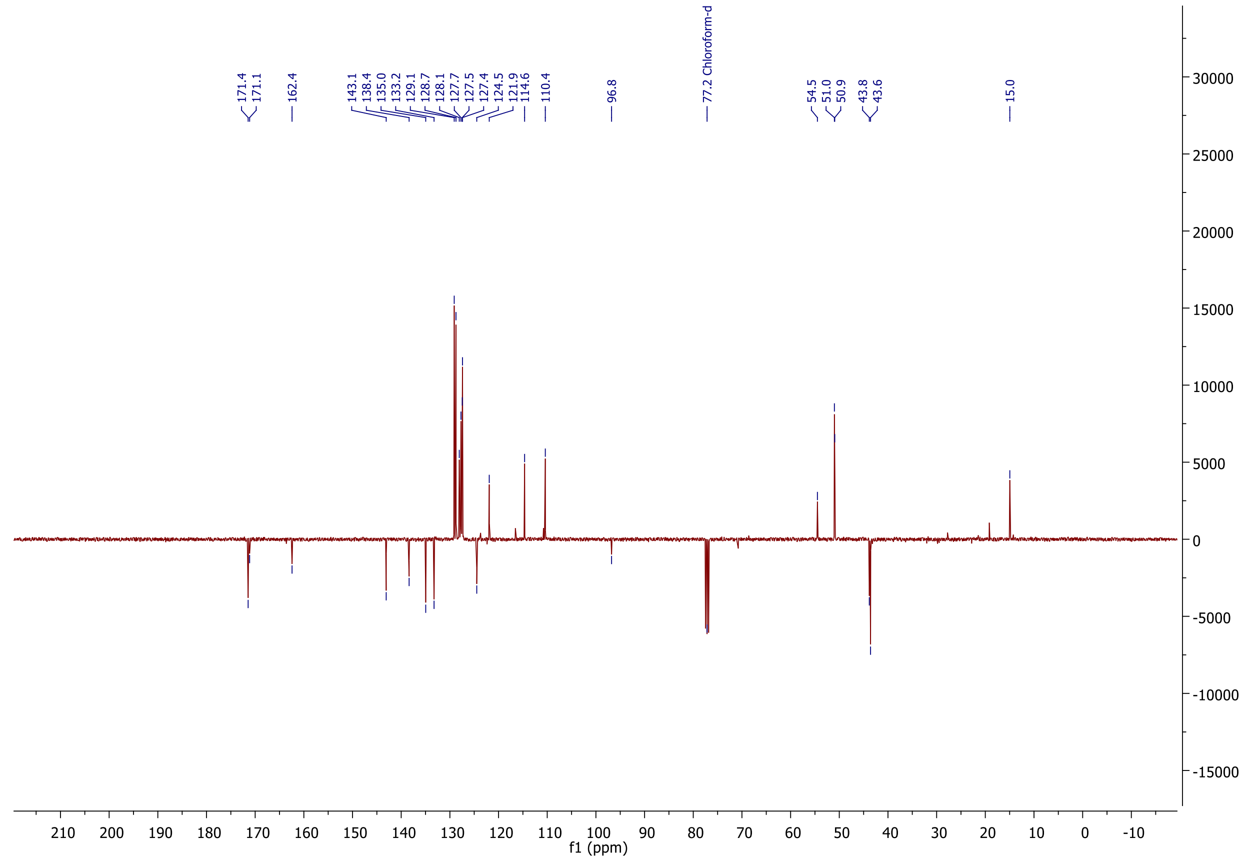
77.2 Chloroform-d
f1 (ppm)

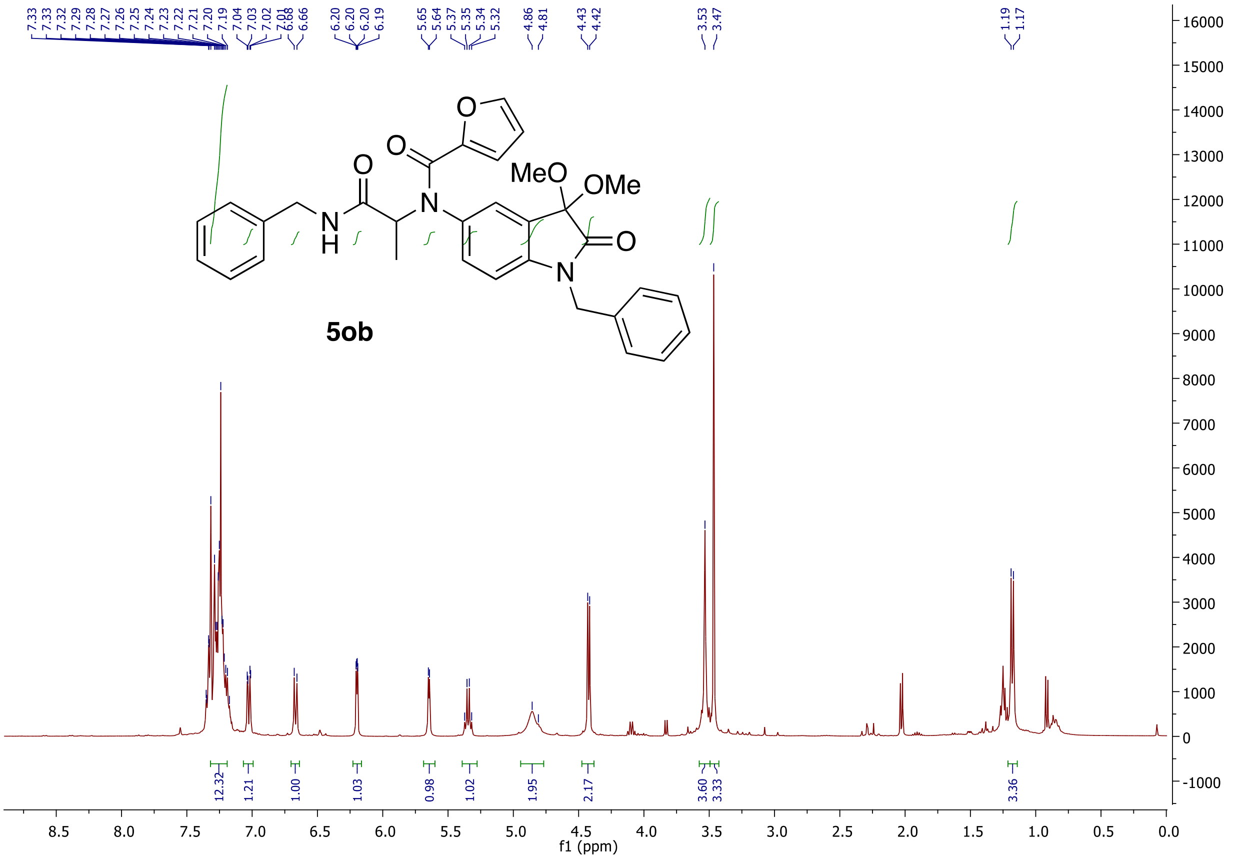
7.33
7.33
7.32
7.29
7.28
7.27
7.26
7.25
7.24
7.23
7.22
7.21
7.20
7.19
7.04
7.03
7.02
7.01
6.68
6.66
6.20
6.20
6.20
6.19
5.65
5.64
5.37
5.35
5.34
5.32
4.86
4.81
4.43
4.42
3.53
3.47
1.19
1.17
MeO
OMe
O
N
H
5ob
12.32
1.21
1.00
1.03
0.98
1.02
1.95
2.17
3.60
3.33
3.36
f1 (ppm)

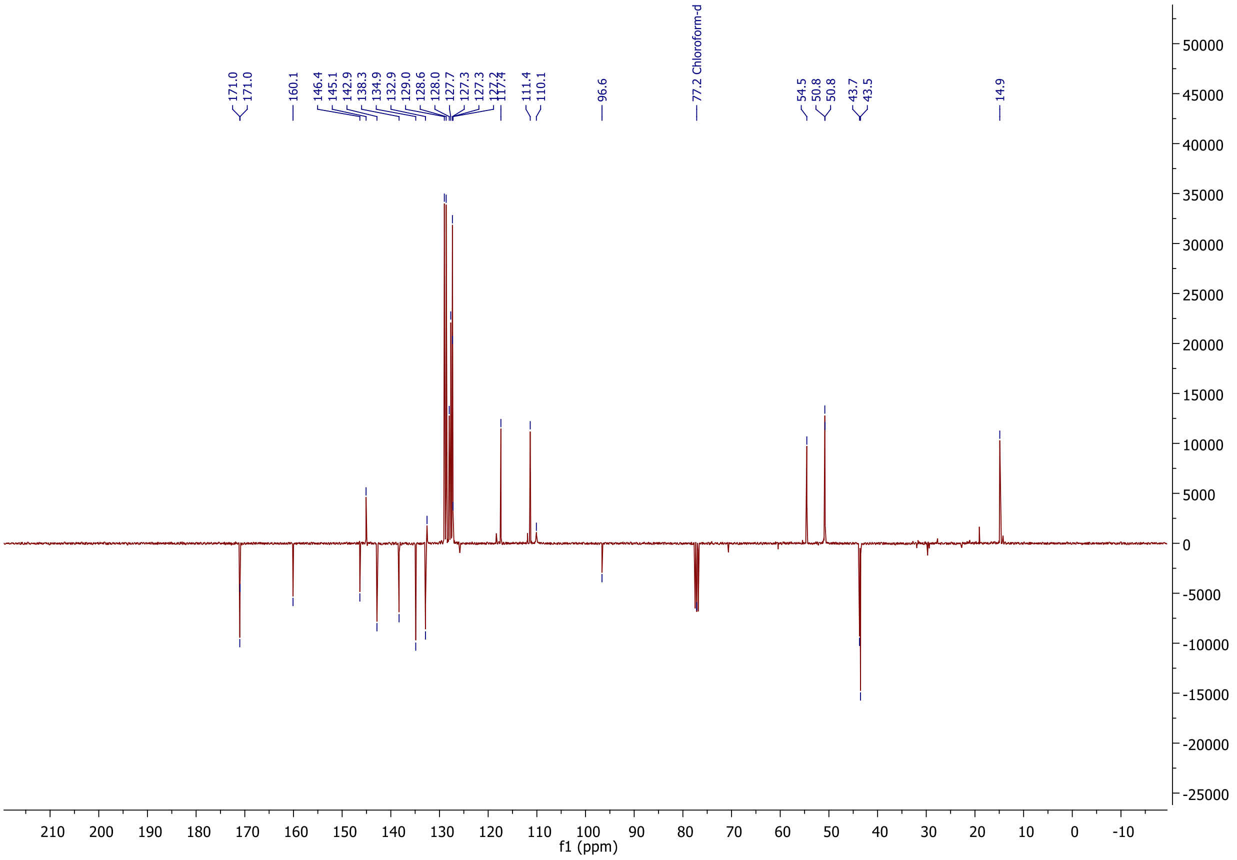
171.0
171.0
160.1
146.4
145.1
142.9
138.3
134.9
132.9
129.0
128.6
128.0
127.7
127.3
127.3
117.4
111.4
110.1
96.6
77.2 Chloroform-d
54.5
50.8
50.8
43.7
43.5
14.9
f1 (ppm)

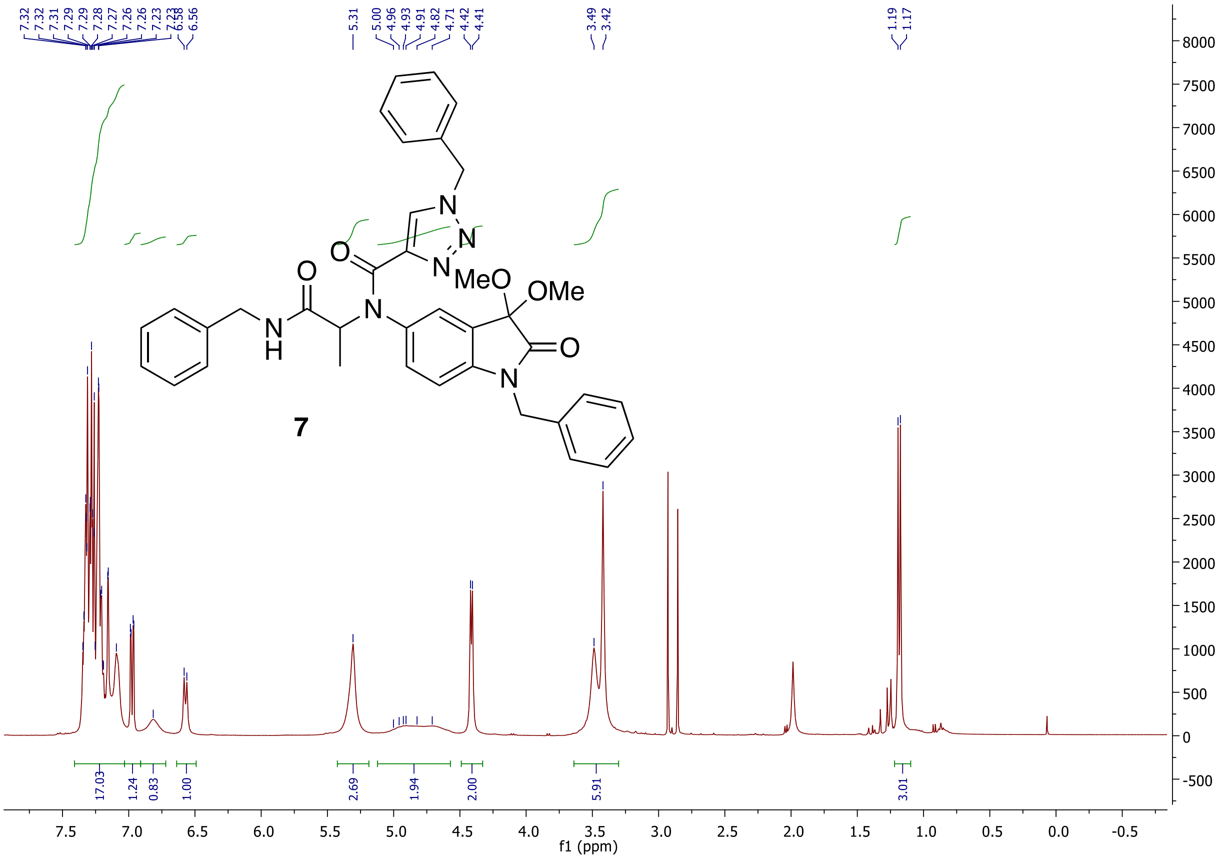
7.32 7.32 7.31 7.29 7.29 7.28 7.27 7.26 7.26 7.23 7.23 6.58 6.56
5.31 5.00 4.96 4.93 4.91 4.82 4.71 4.42 4.41
3.49 3.42
1.19 1.17
17.03 1.24 0.83 1.00 2.69 1.94 2.00 5.91 3.01
7
f1 (ppm)

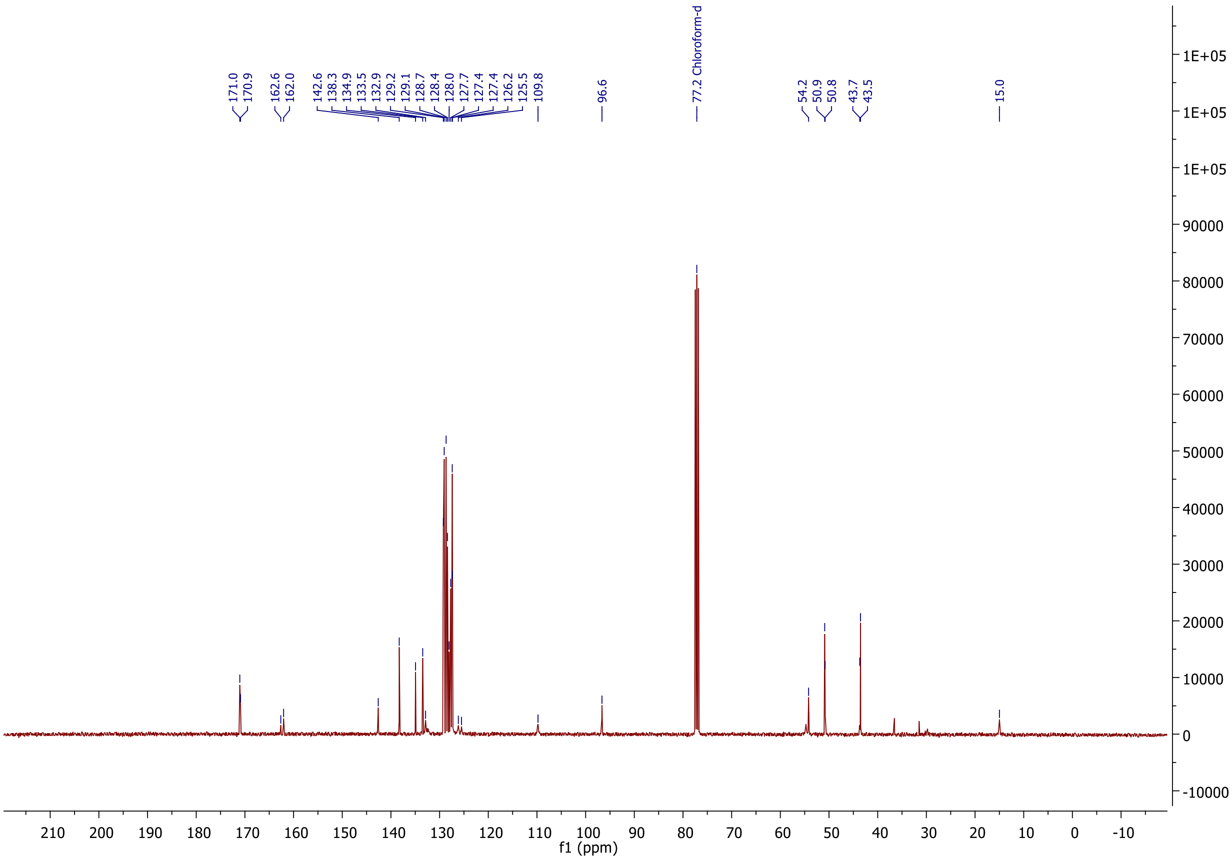
171.0 170.9 162.6 162.0 142.6 138.3 134.9 133.5 132.9 129.2 129.1 128.7 128.4 128.0 127.7 127.4 127.4 126.2 125.5 109.8 96.6
77.2 Chloroform-d
54.2 50.9 50.8 43.7 43.5 15.0
f1 (ppm)

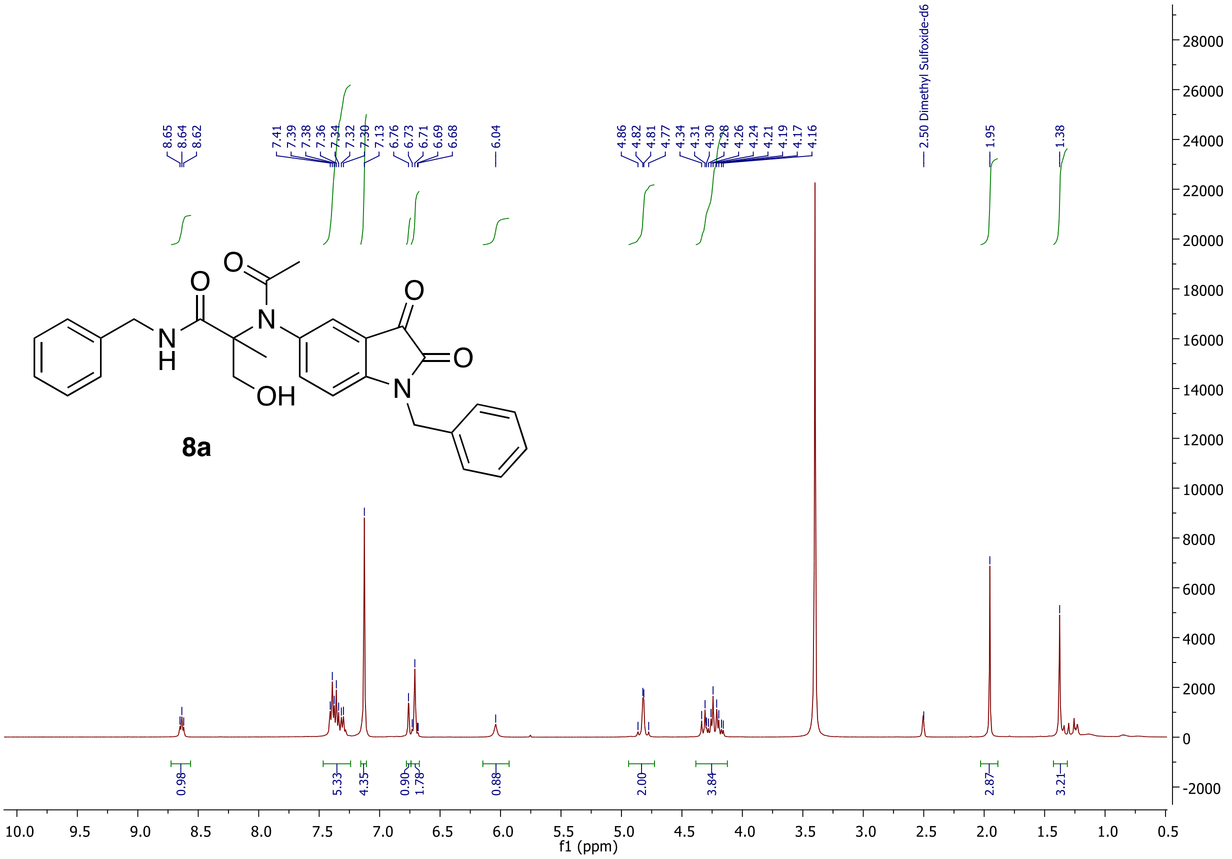
8a
8.65 8.64 8.62
7.41 7.39 7.38 7.36 7.34 7.32 7.30 7.13 6.76 6.73 6.71 6.69 6.68
6.04
4.86 4.82 4.81 4.77 4.34 4.31 4.30 4.28 4.26 4.24 4.21 4.19 4.17 4.16
2.50 Dimethyl Sulfoxide-d6
1.95
1.38
0.98 5.33 4.35 0.90 1.78 0.88 2.00 3.84 2.87 3.21
f1 (ppm)

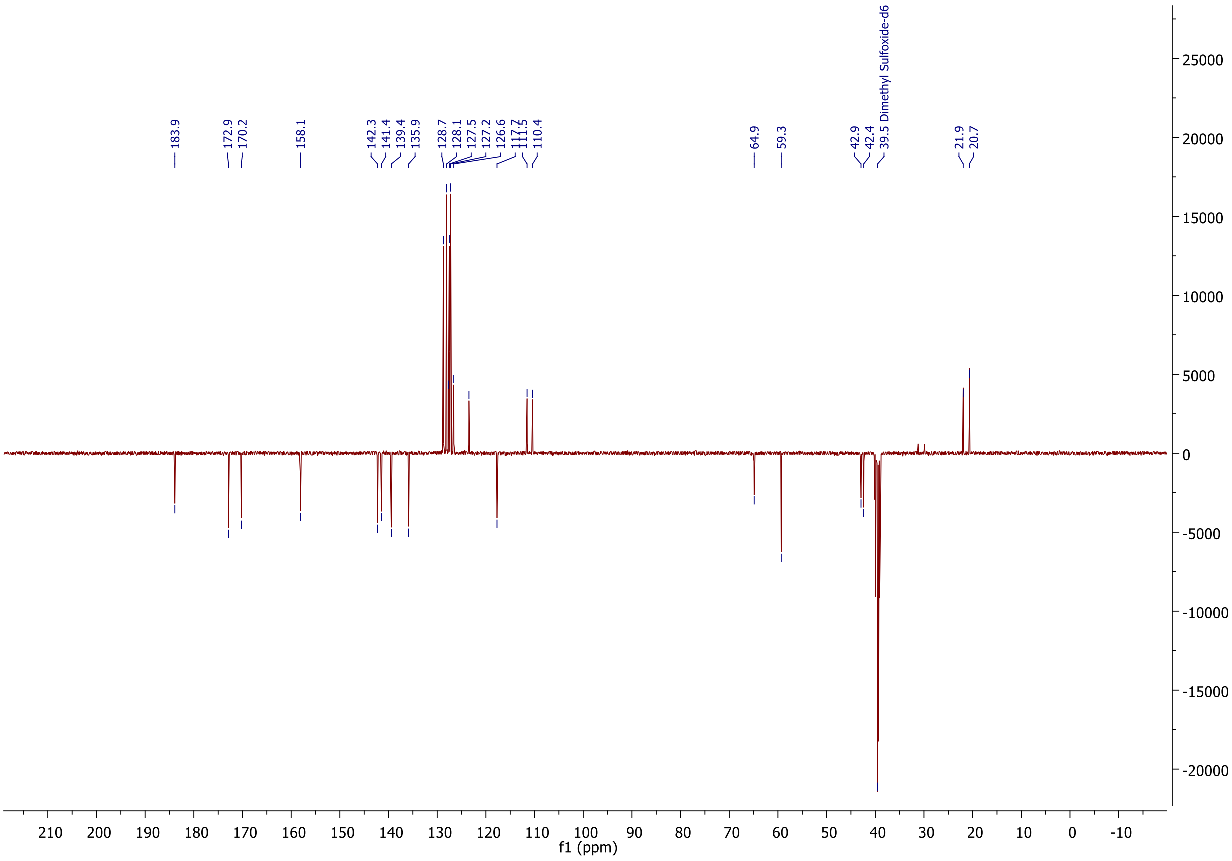
183.9
172.9 170.2
158.1
142.3 141.4 139.4 135.9
128.7 128.1 127.5 127.2 126.6 117.7 111.5 110.4
64.9 59.3
42.9 42.4 39.5 Dimethyl Sulfoxide-d6
21.9 20.7
f1 (ppm)

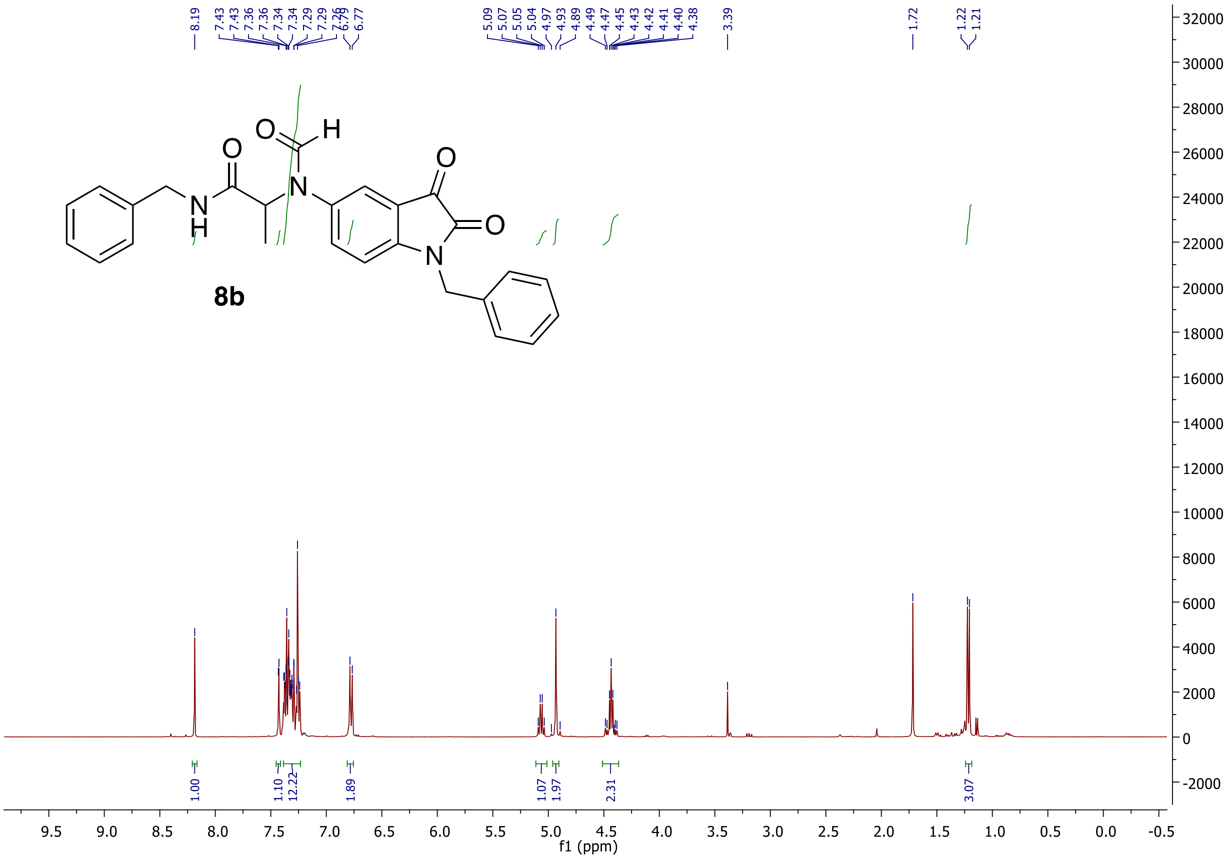
8.19
7.43 7.43 7.36 7.36 7.34 7.34 7.29 7.29 7.26 6.79 6.77
5.09 5.07 5.05 5.04 4.97 4.93 4.89 4.49 4.47 4.45 4.43 4.42 4.41 4.40 4.38
3.39
1.72
1.22 1.21
O
H
N
8b
1.00
1.10
12.22
1.89
1.07
1.97
2.31
3.07
f1 (ppm)

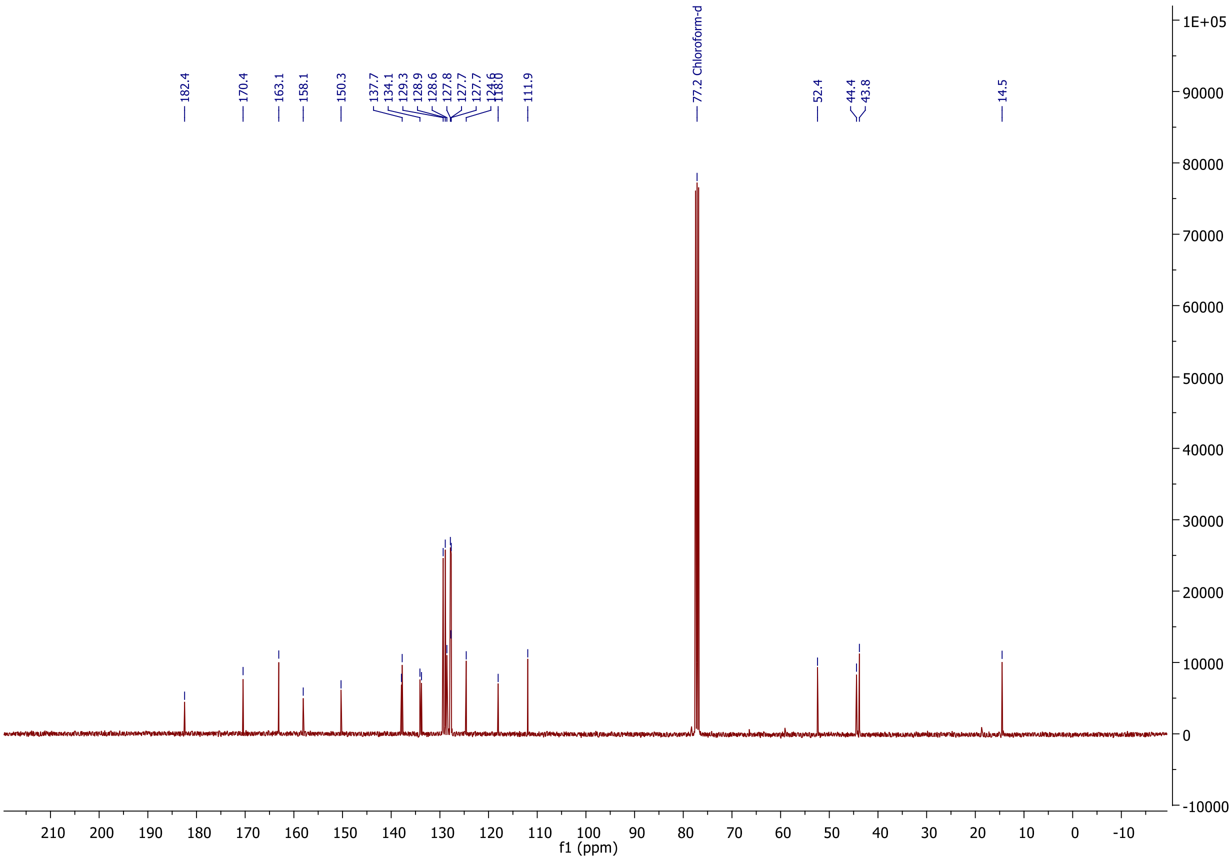
182.4
170.4
163.1
158.1
150.3
137.7 134.1 129.3 128.9 128.6 127.8 127.7 127.7
111.9
77.2 Chloroform-d
52.4
44.4 43.8
14.5
f1 (ppm)

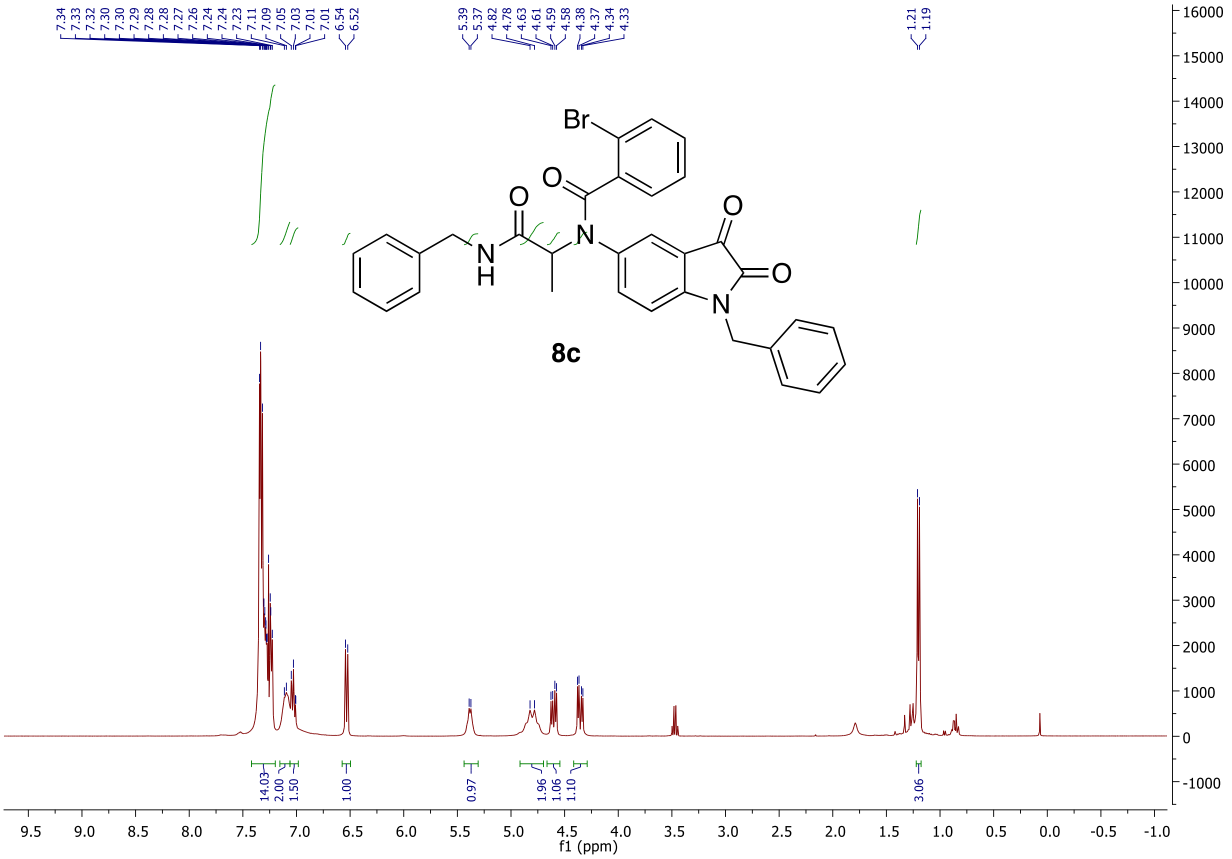
7.34 7.33 7.32 7.30 7.30 7.29 7.28 7.28 7.27 7.26 7.24 7.24 7.23 7.11 7.09 7.05 7.03 7.01 6.54 6.52
5.39 5.37 4.82 4.78 4.63 4.61 4.59 4.58 4.38 4.37 4.34 4.33
1.21 1.19
Br
O
O
O
O
N
N
N
H
8c
14.03 2.00 1.50 1.00 0.97 1.96 1.06 1.10 3.06
f1 (ppm)

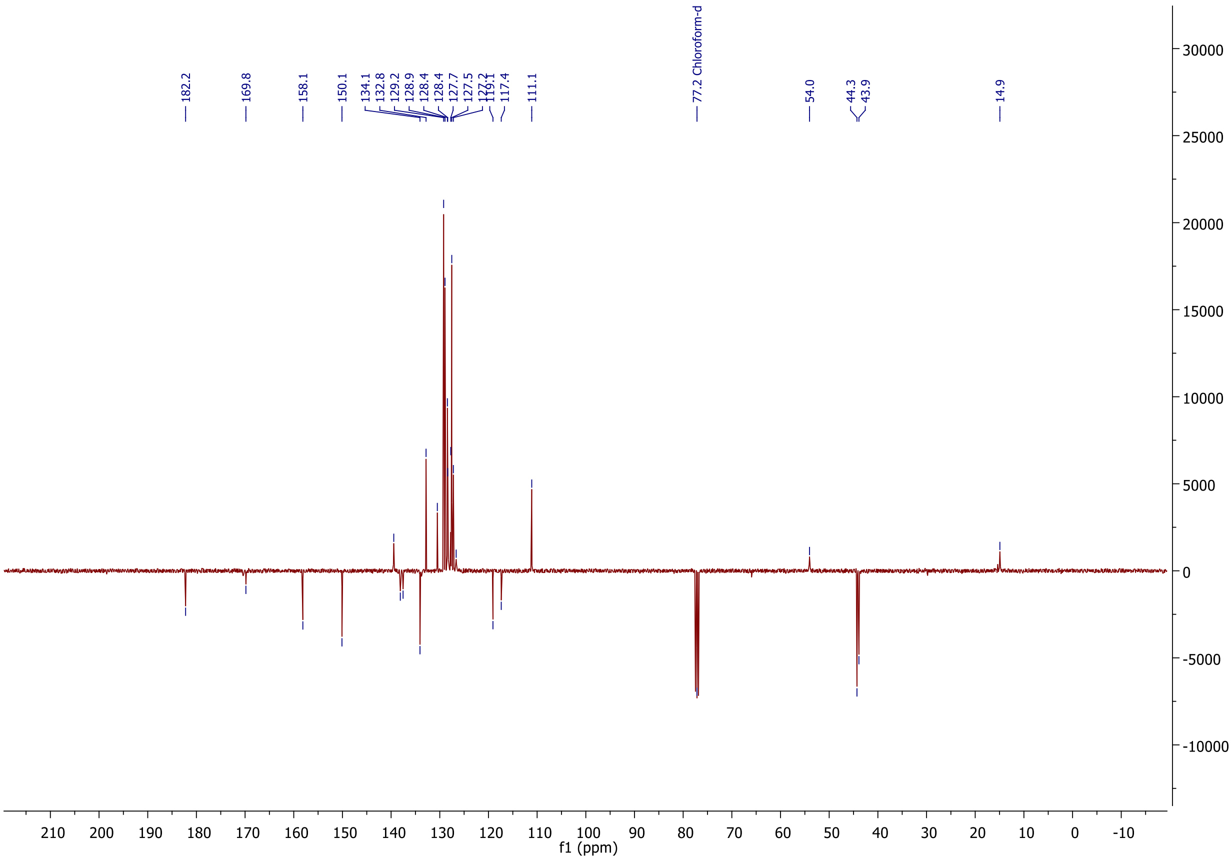
182.2
169.8
158.1
150.1
134.1 132.8 129.2 128.9 128.4 127.7 127.5 122.2 119.1 117.4
111.1
77.2 Chloroform-d
54.0
44.3 43.9
14.9
f1 (ppm)

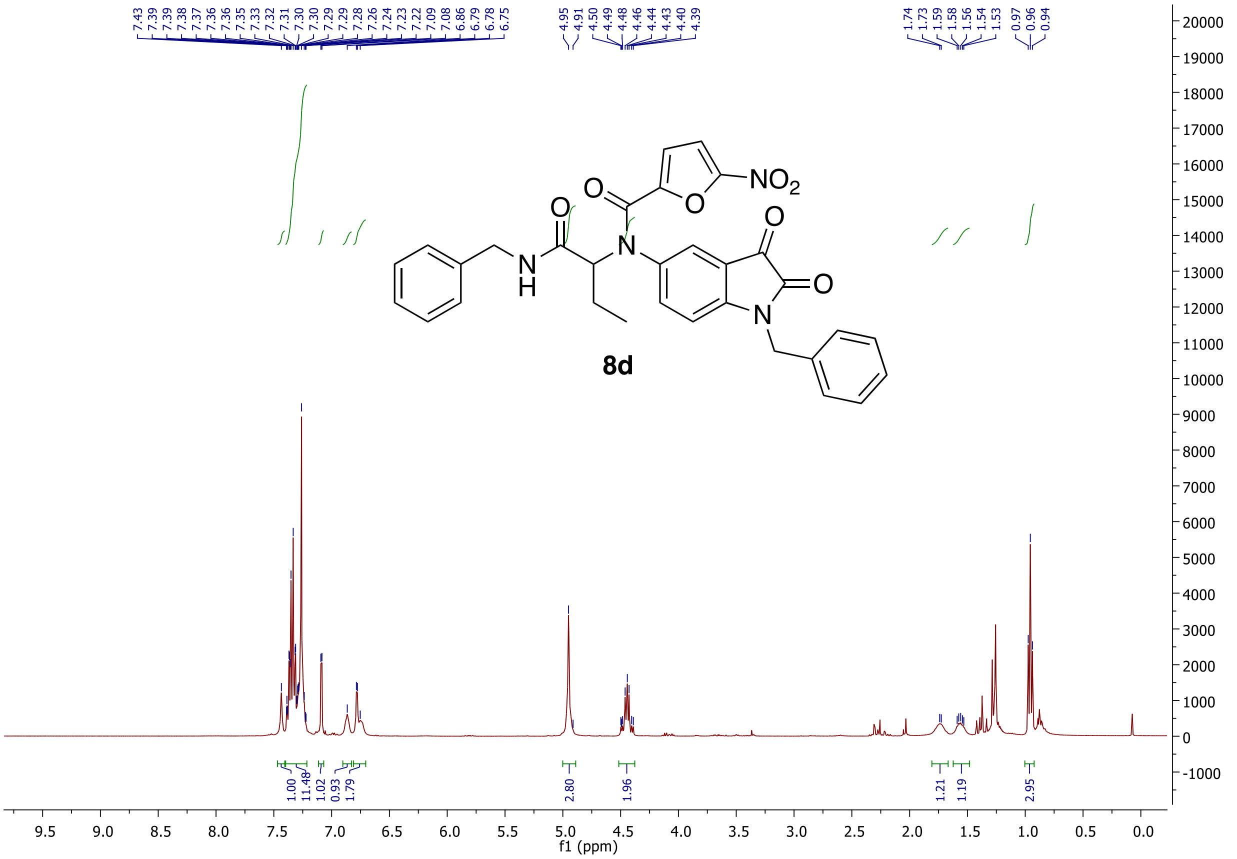
7.43 7.39 7.39 7.38 7.37 7.36 7.36 7.35 7.33 7.32 7.31 7.30 7.30 7.29 7.29 7.28 7.26 7.24 7.23 7.22 7.09 7.08 6.86 6.79 6.78 6.75
4.95 4.91 4.50 4.49 4.48 4.46 4.44 4.43 4.40 4.39
1.74 1.73 1.59 1.58 1.56 1.54 1.53 0.97 0.96 0.94
8d
1.00 11.48 1.02 0.93 1.79 2.80 1.96 1.21 1.19 2.95
f1 (ppm)

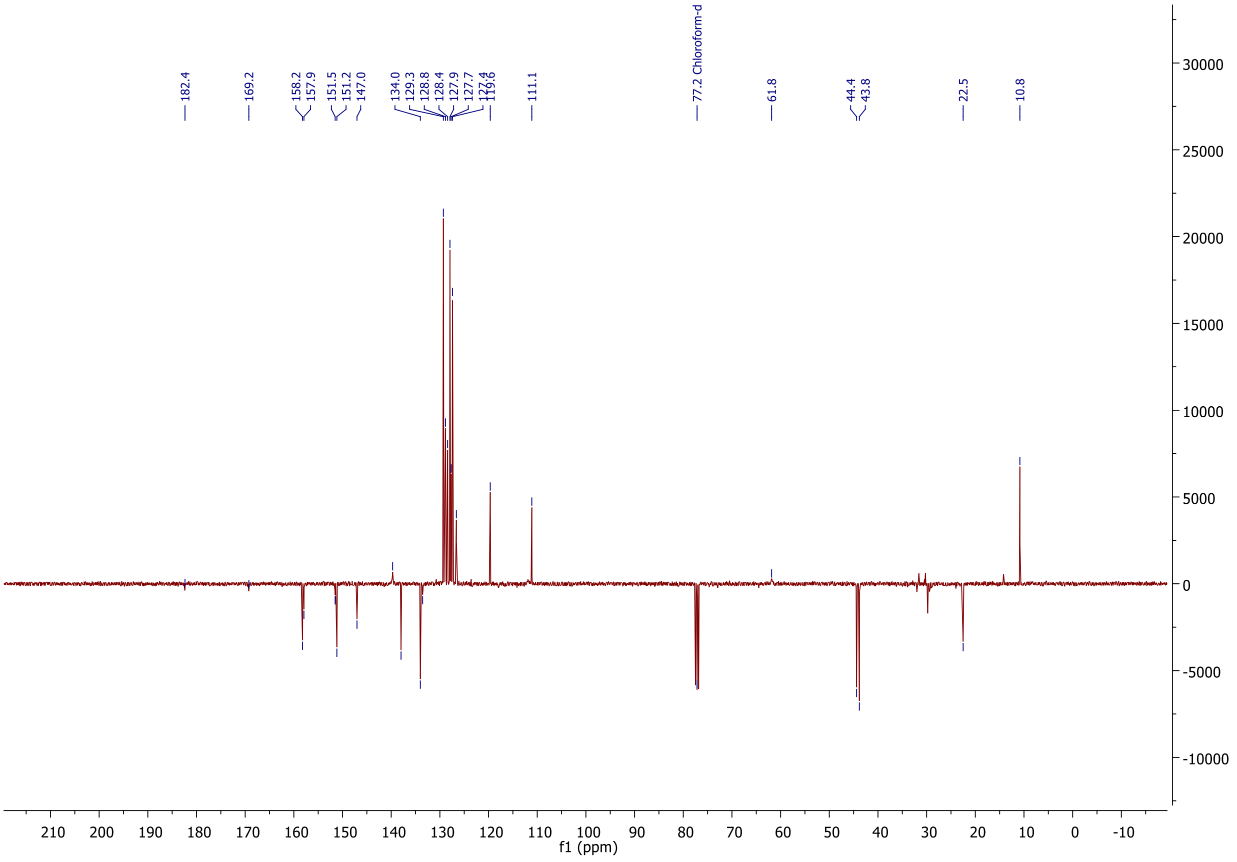
182.4 169.2 158.2 157.9 151.5 151.2 147.0 134.0 129.3 128.8 128.4 127.9 127.7 127.4 119.6 111.1
77.2 Chloroform-d
61.8 44.4 43.8 22.5 10.8
f1 (ppm)

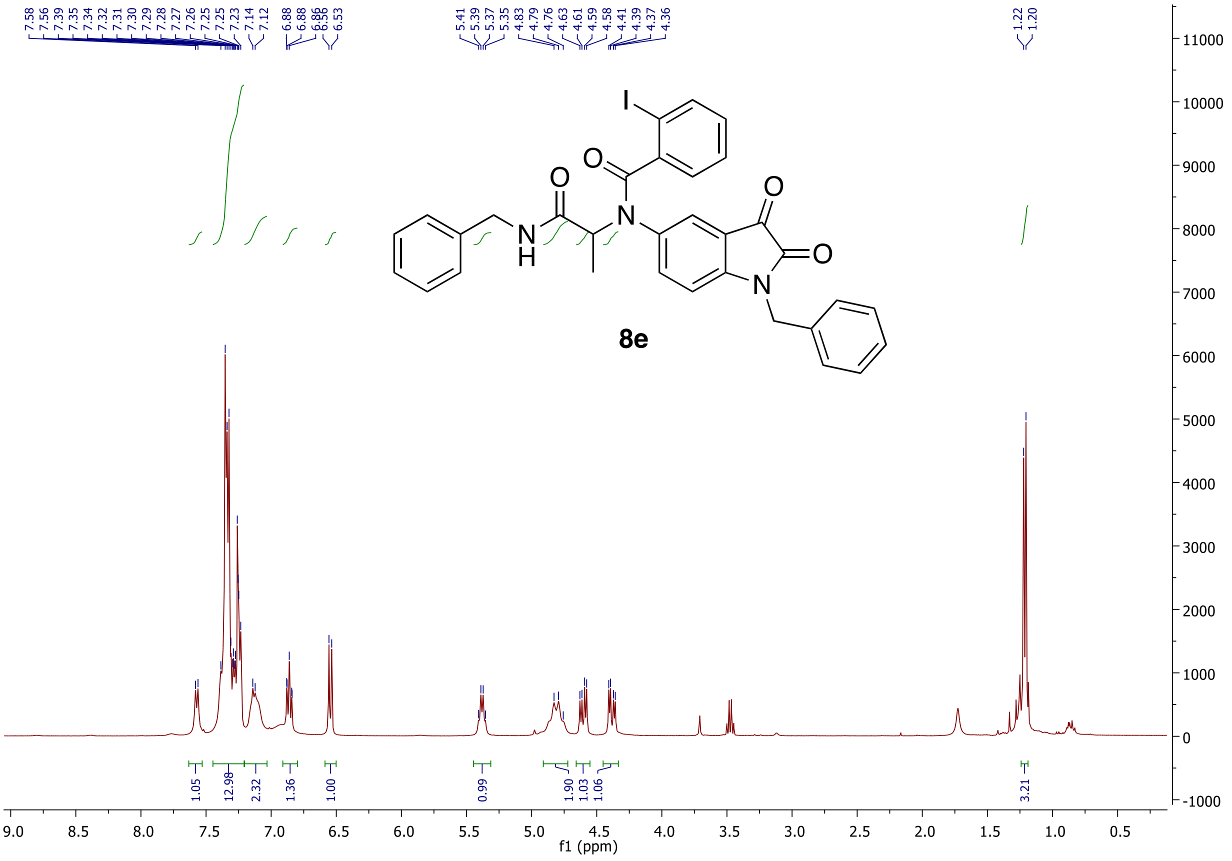
7.58 7.56 7.39 7.35 7.34 7.32 7.31 7.30 7.29 7.28 7.27 7.26 7.25 7.25 7.23 7.14 7.12 6.88 6.88 6.86 6.56 6.53
5.41 5.39 5.37 5.35 4.83 4.79 4.76 4.63 4.61 4.59 4.58 4.41 4.39 4.37 4.36
1.22 1.20
I
O
O
N
H
N
O
O
N
8e
1.05 12.98 2.32 1.36 1.00 0.99 1.90 1.03 1.06 3.21
f1 (ppm)

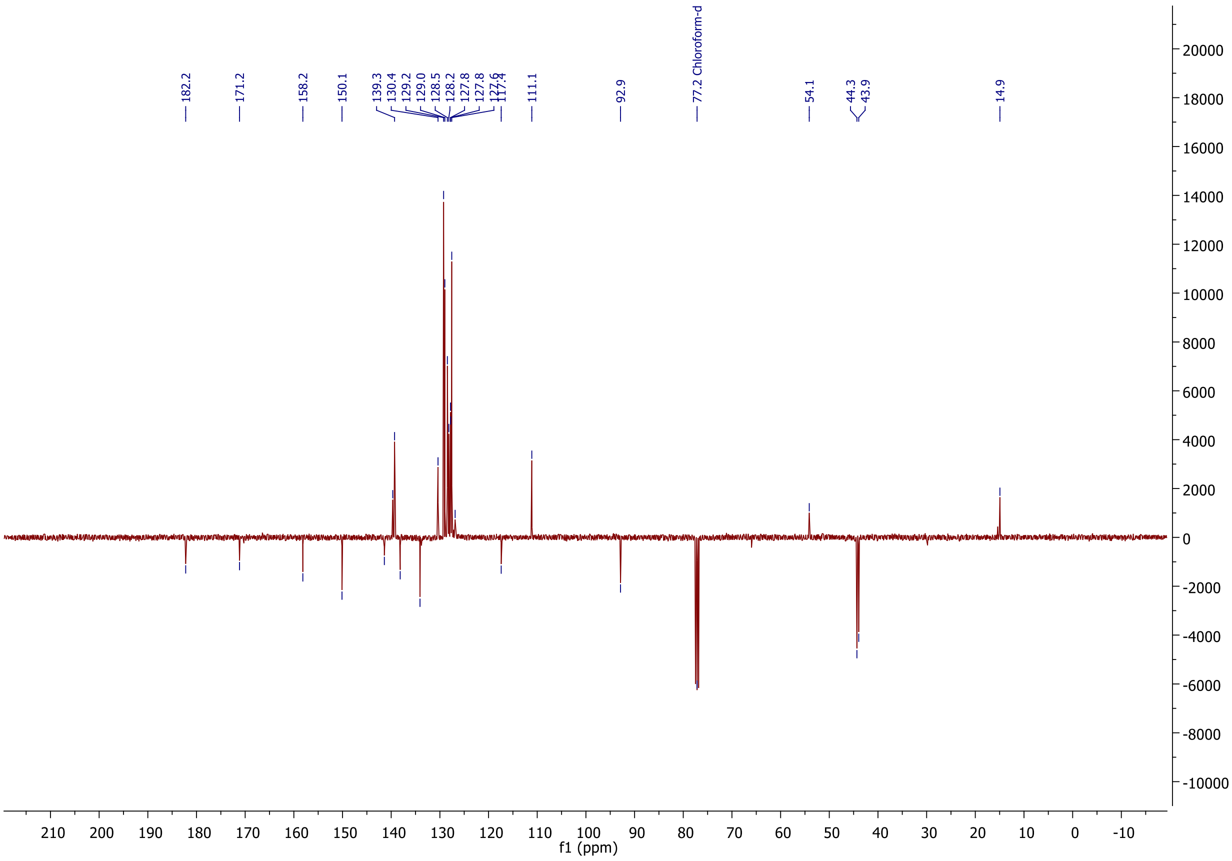
182.2 171.2 158.2 150.1 139.3 130.4 129.2 129.0 128.5 128.2 127.8 127.6 117.4 111.1 92.9
77.2 Chloroform-d
54.1 44.3 43.9 14.9
f1 (ppm)

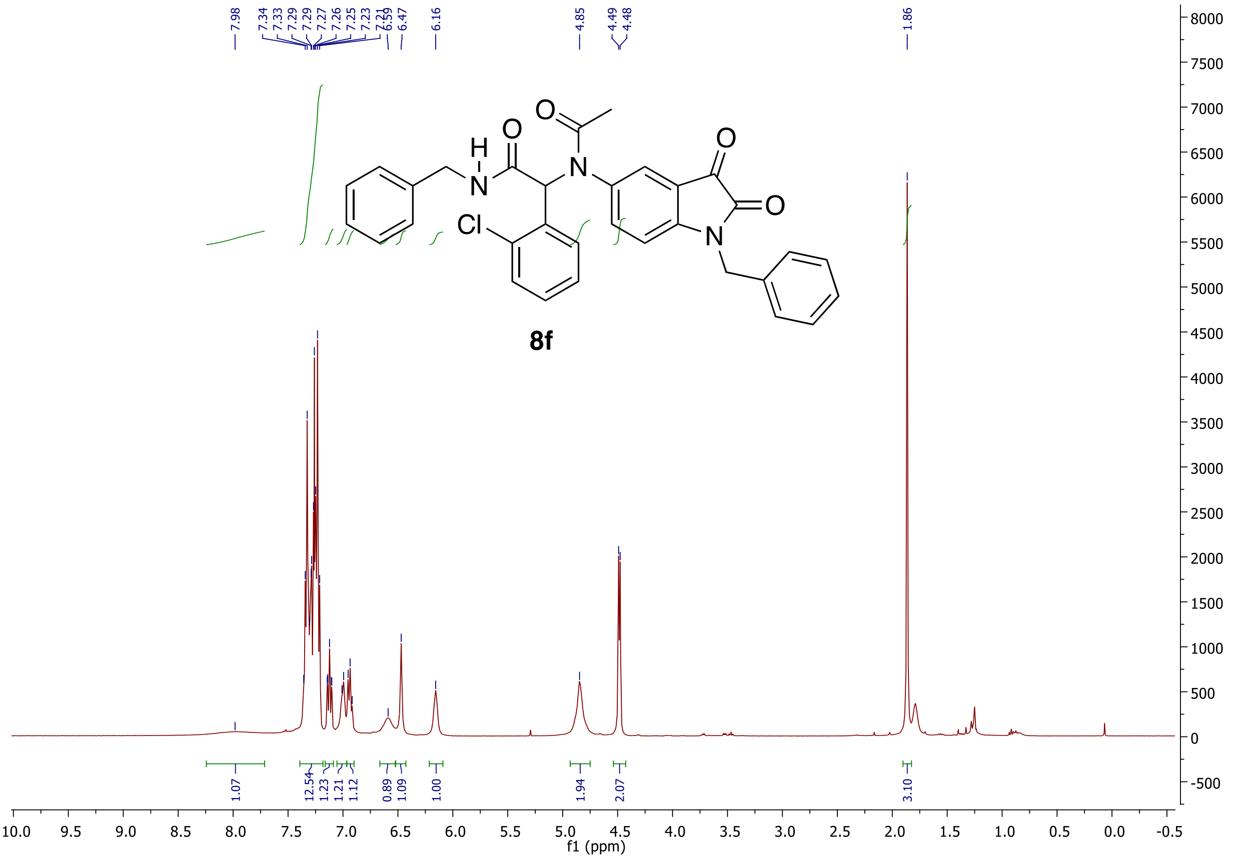

7.98
7.34
7.33
7.29
7.29
7.27
7.26
7.25
7.23
6.59
6.47
6.16
4.85
4.49
4.48
1.86
O
H
N
N
O
O
O
N
Cl
8f
1.07
12.54
1.23
1.21
1.12
0.89
1.09
1.00
1.94
2.07
3.10
f1 (ppm)

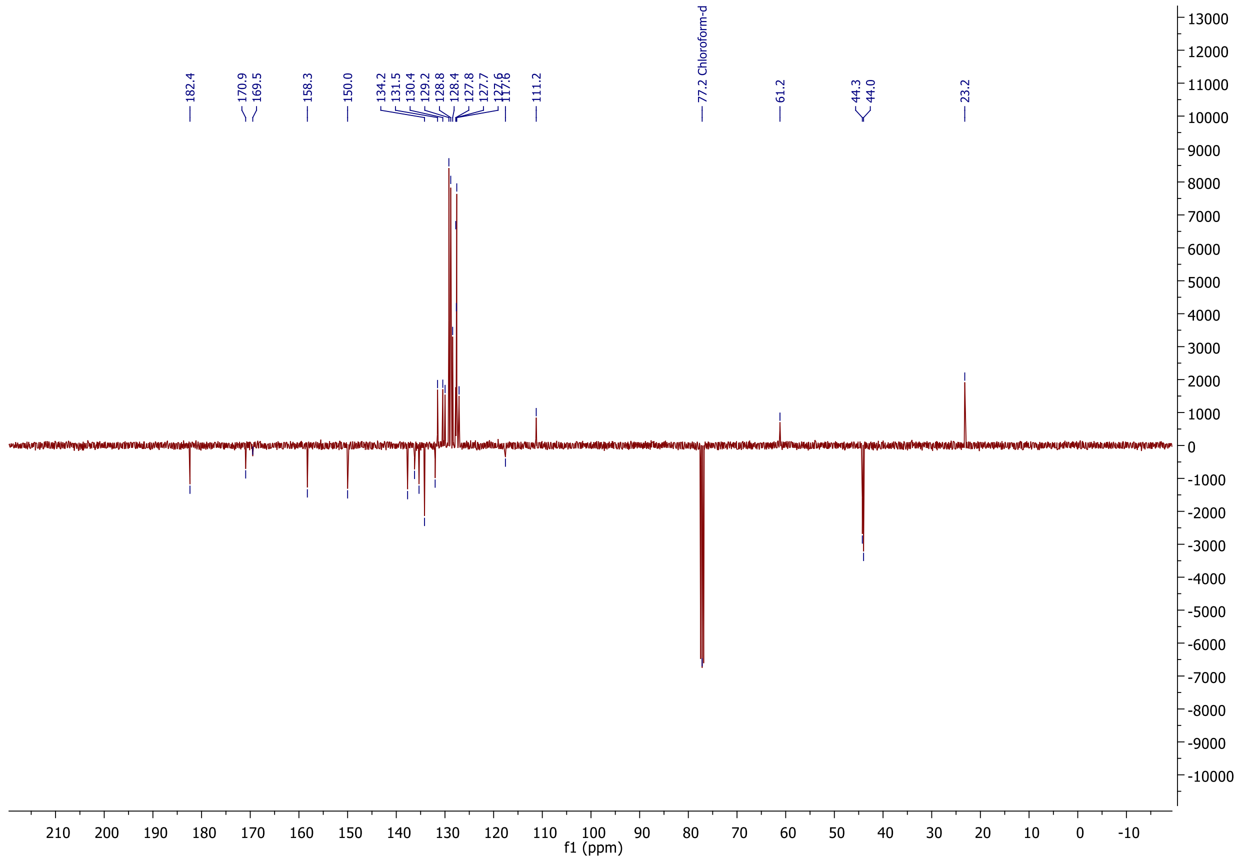

182.4
170.9
169.5
158.3
150.0
134.2
131.5
130.4
129.2
128.8
128.4
127.8
127.7
111.2
77.2 Chloroform-d
61.2
44.3
44.0
23.2
f1 (ppm)

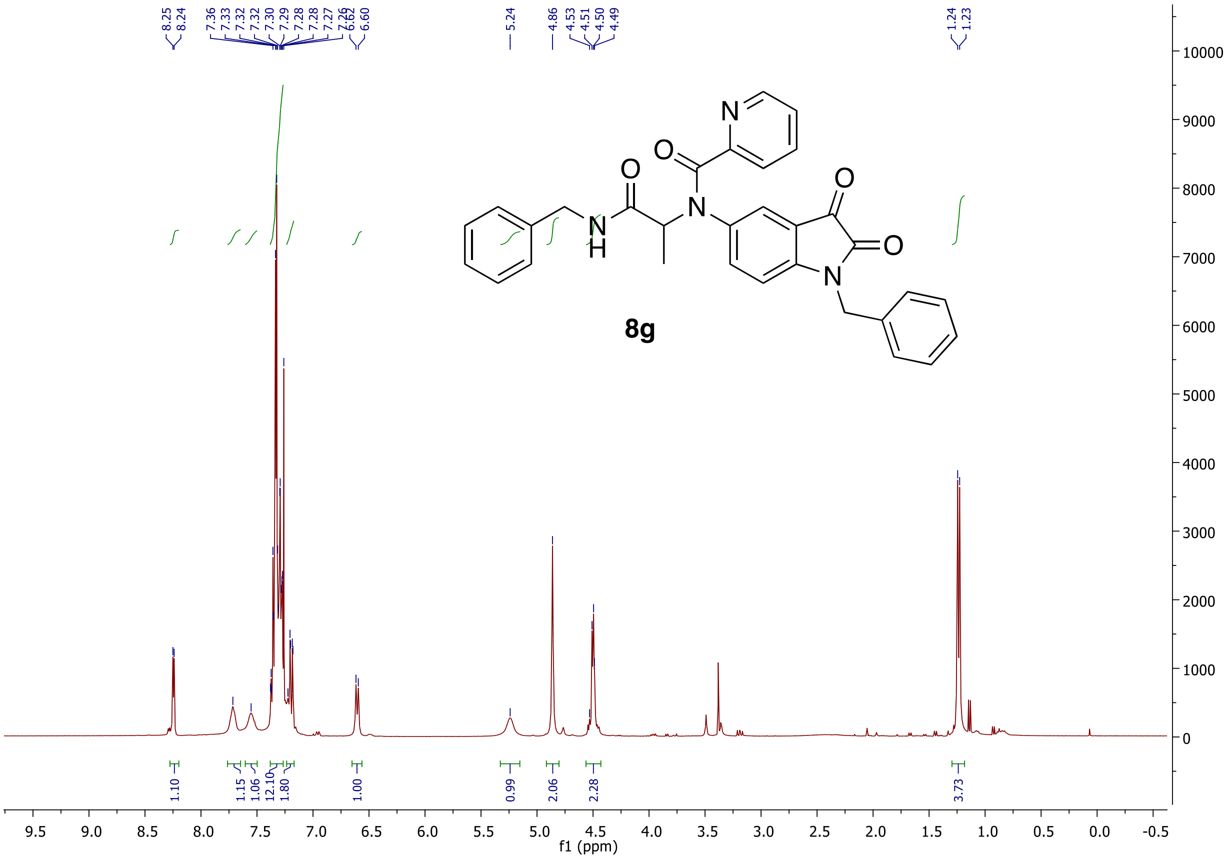
8.25
8.24
7.36
7.33
7.32
7.32
7.30
7.29
7.28
7.28
7.27
6.62
6.60
5.24
4.86
4.53
4.51
4.50
4.49
1.24
1.23
8g
1.10
1.15
1.06
12.10
1.80
1.00
0.99
2.06
2.28
3.73
f1 (ppm)

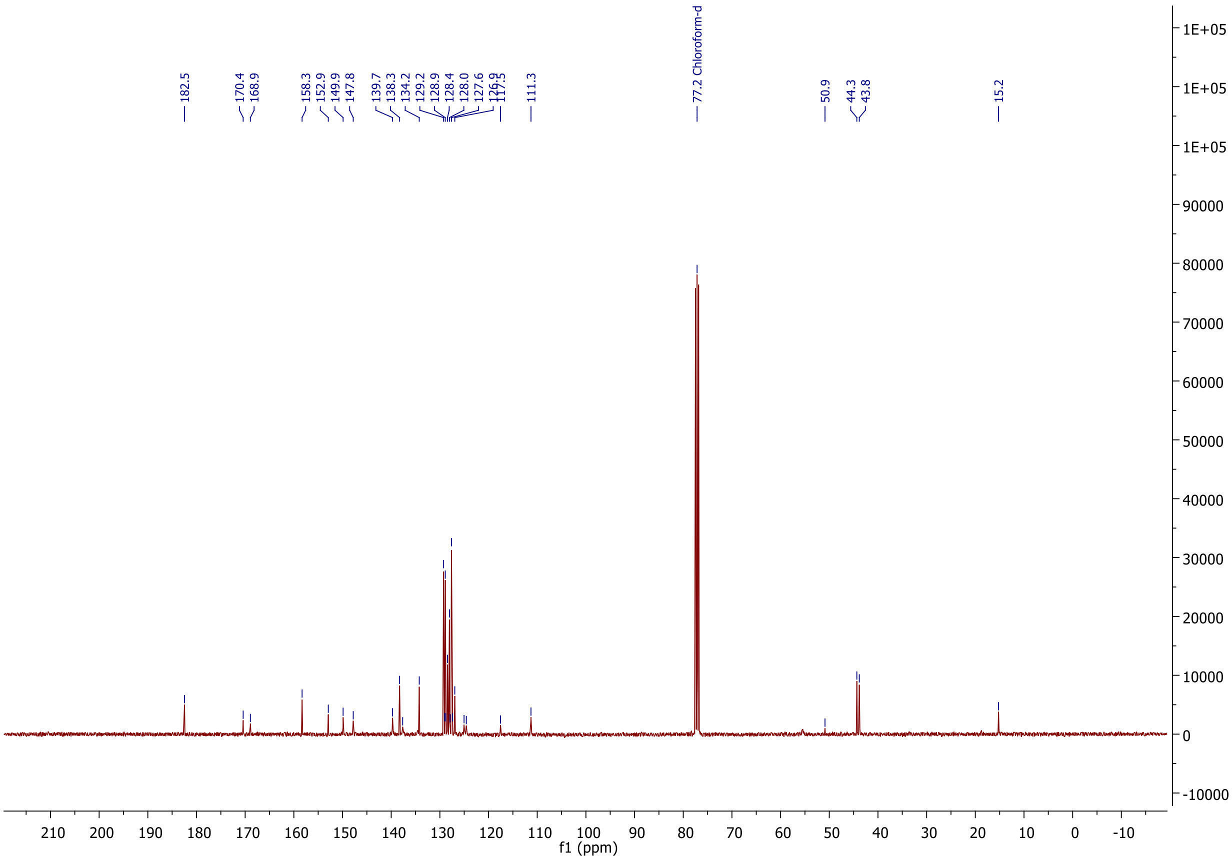
182.5
170.4
168.9
158.3
152.9
149.9
147.8
139.7
138.3
134.2
129.2
128.9
128.4
128.0
127.6
111.3
77.2 Chloroform-d
50.9
44.3
43.8
15.2
f1 (ppm)

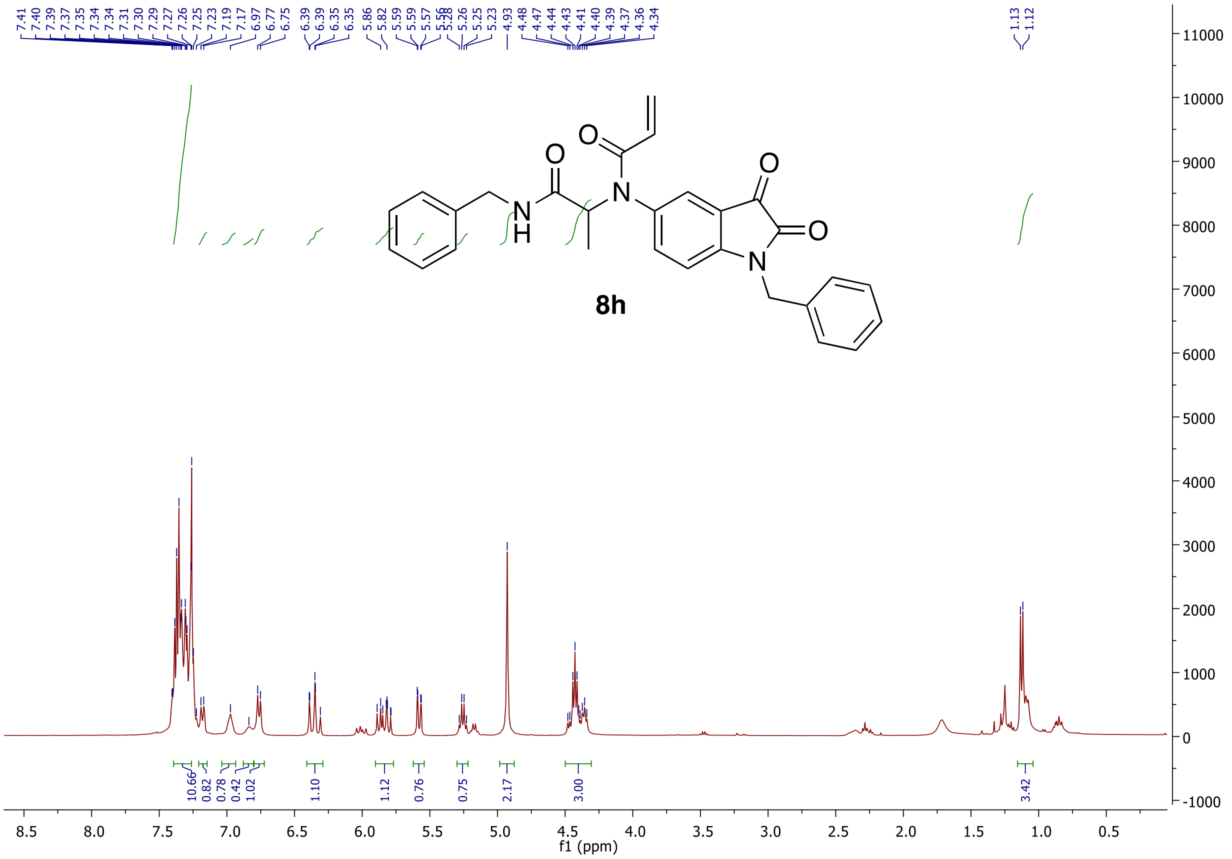
8h
f1 (ppm)

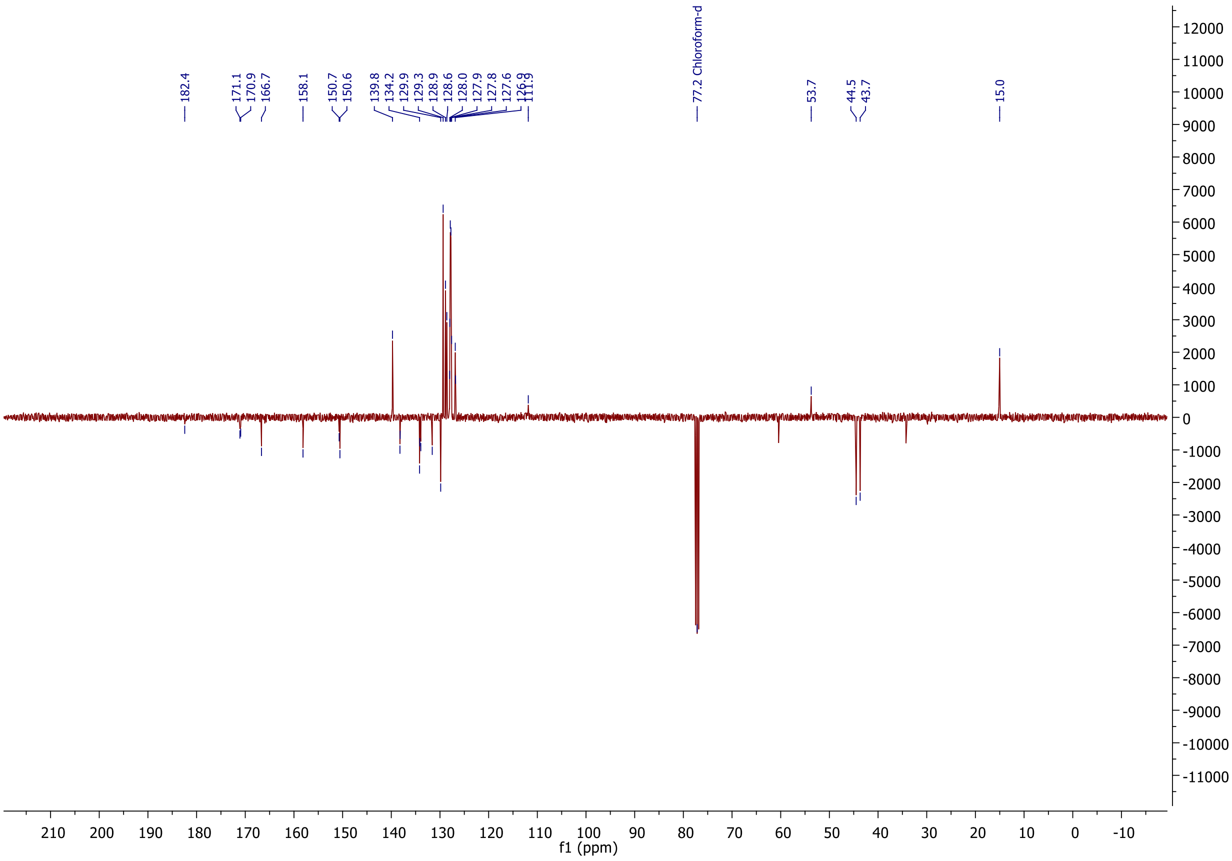
77.2 Chloroform-d
f1 (ppm)

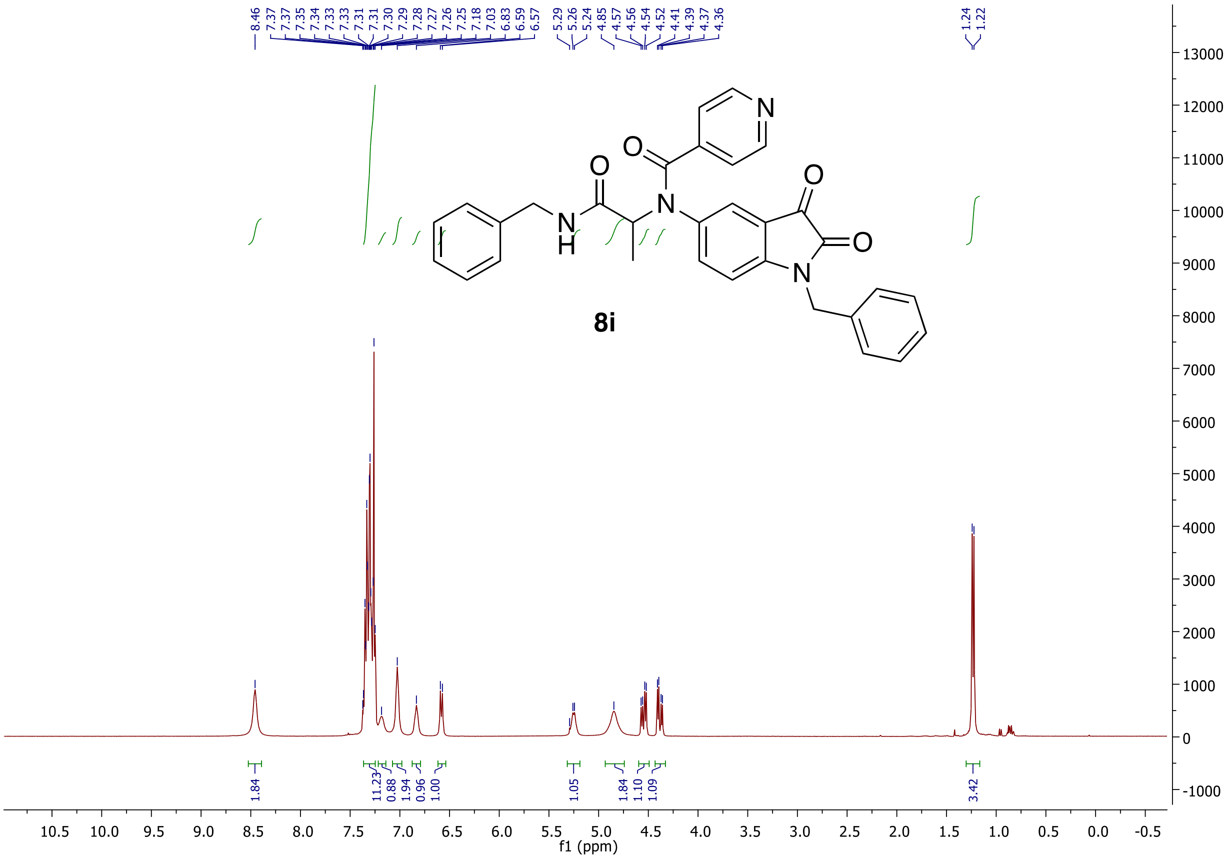
8.46
7.37
7.37
7.35
7.34
7.33
7.33
7.31
7.31
7.30
7.29
7.28
7.27
7.26
7.25
7.18
7.03
6.83
6.59
6.57
5.29
5.26
5.24
4.85
4.57
4.56
4.54
4.52
4.41
4.39
4.37
4.36
1.24
1.22
8i
1.84
11.23
0.88
1.94
0.96
1.00
1.05
1.84
1.10
1.09
3.42
f1 (ppm)

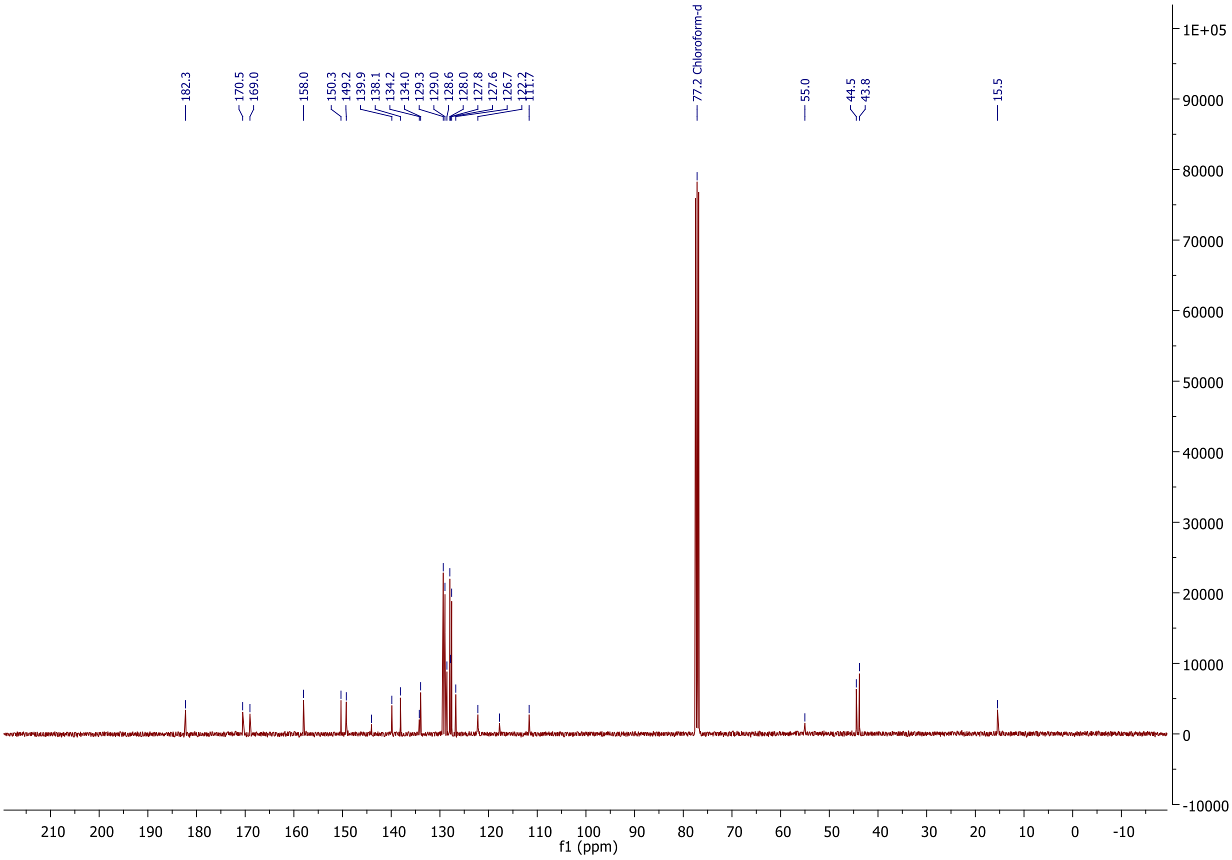
182.3
170.5
169.0
158.0
150.3
149.2
139.9
138.1
134.2
134.0
129.3
129.0
128.6
128.0
127.8
127.6
126.7
122.2
111.7
77.2 Chloroform-d
55.0
44.5
43.8
15.5
f1 (ppm)

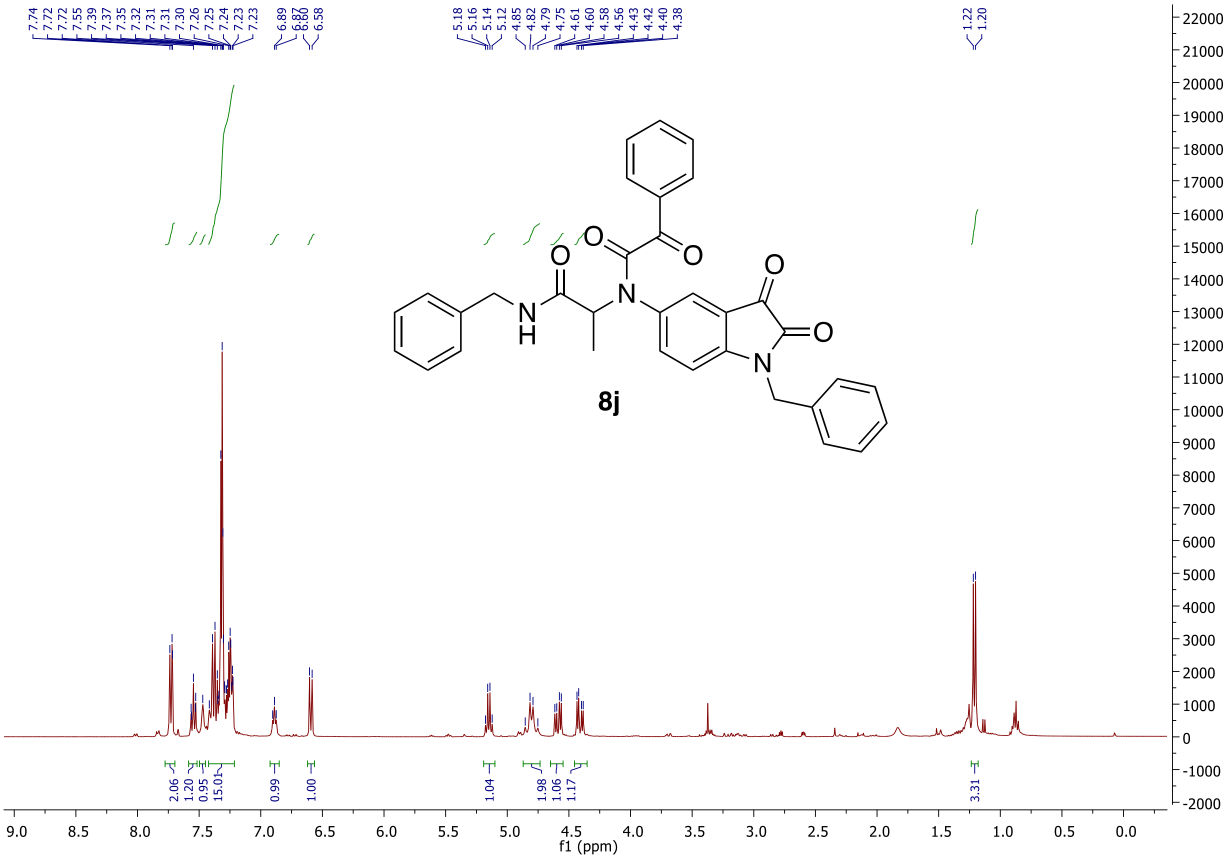
7.74
7.72
7.72
7.55
7.39
7.37
7.35
7.32
7.31
7.31
7.30
7.26
7.25
7.24
7.23
7.23
6.89
6.87
6.60
6.58
5.18
5.16
5.14
5.12
4.85
4.82
4.79
4.75
4.61
4.60
4.58
4.56
4.43
4.42
4.40
4.38
1.22
1.20
8j
2.06
1.20
0.95
15.01
0.99
1.00
1.04
1.98
1.06
1.17
3.31
f1 (ppm)

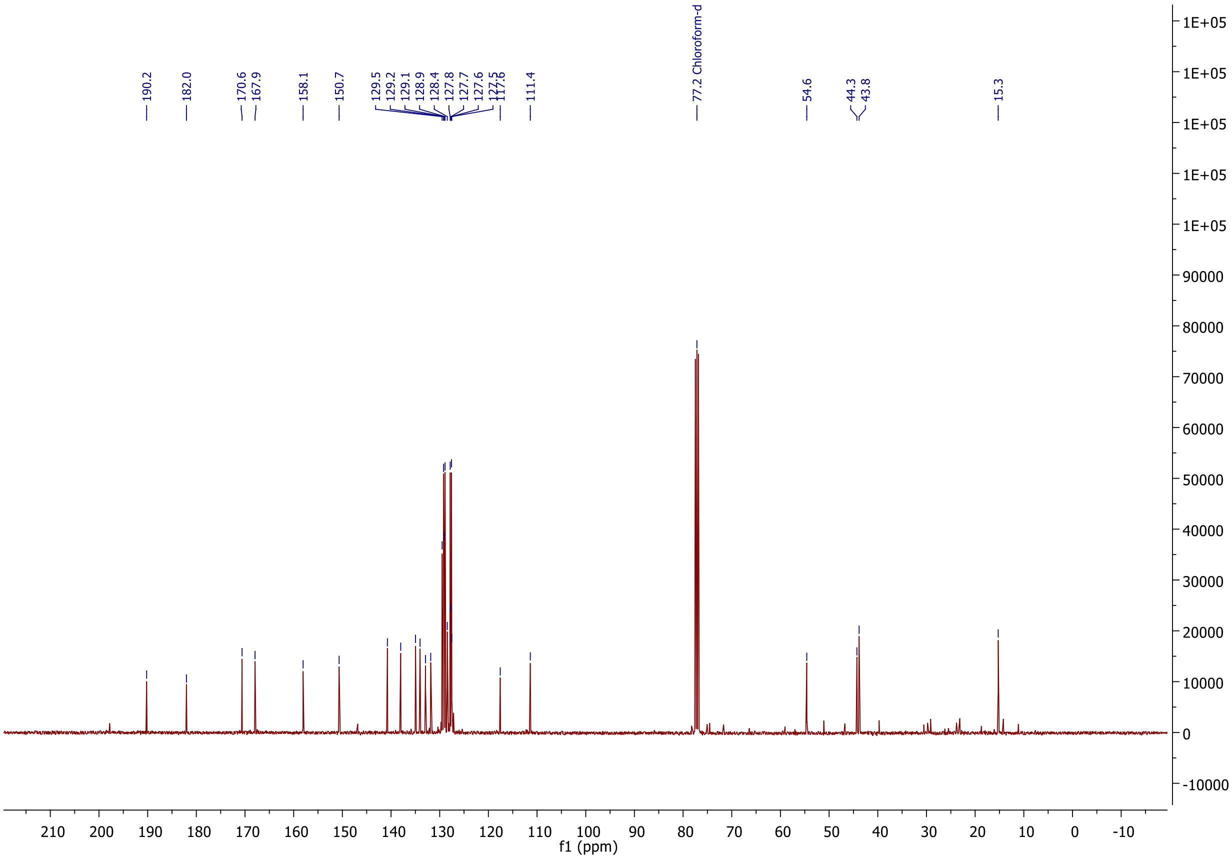
190.2
182.0
170.6
167.9
158.1
150.7
129.5
129.2
129.1
128.9
128.4
127.8
127.7
127.6
127.5
117.6
111.4
77.2 Chloroform-d
54.6
44.3
43.8
15.3
f1 (ppm)

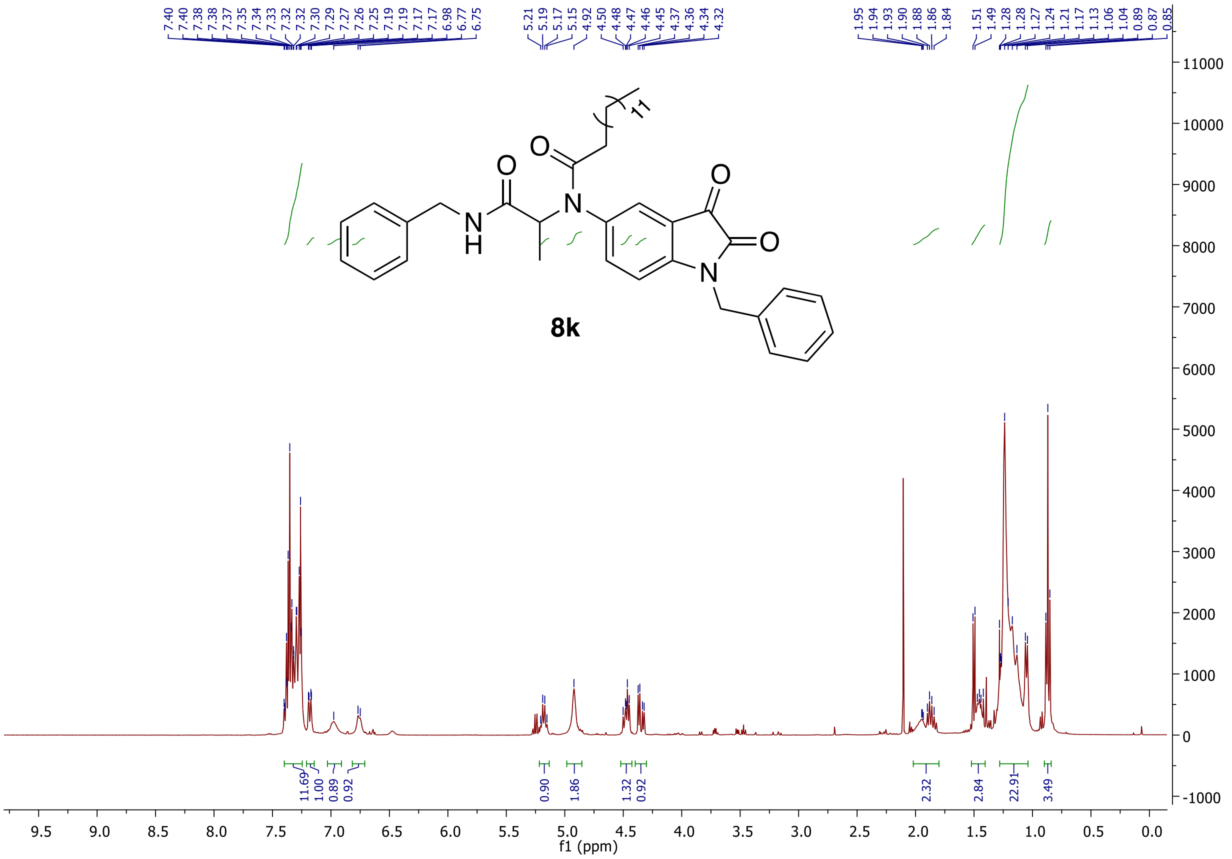

8k

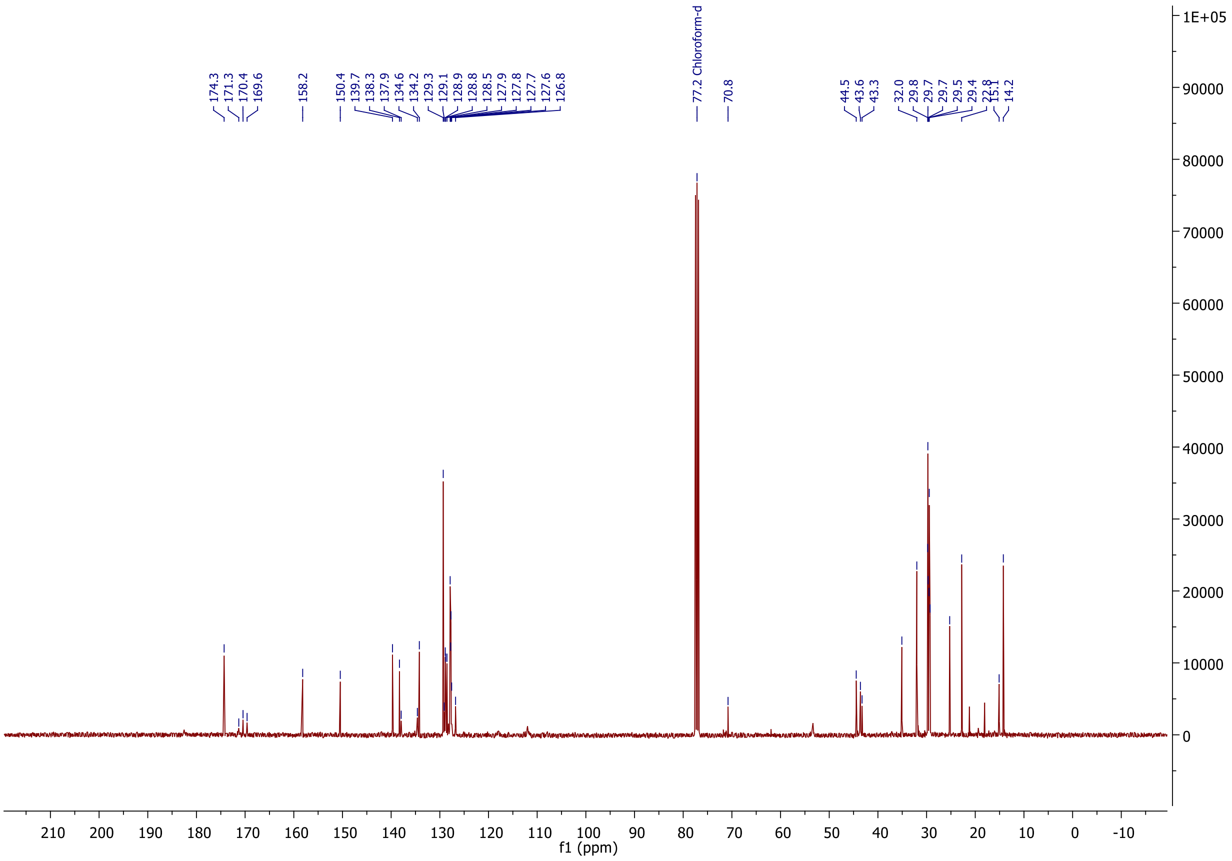

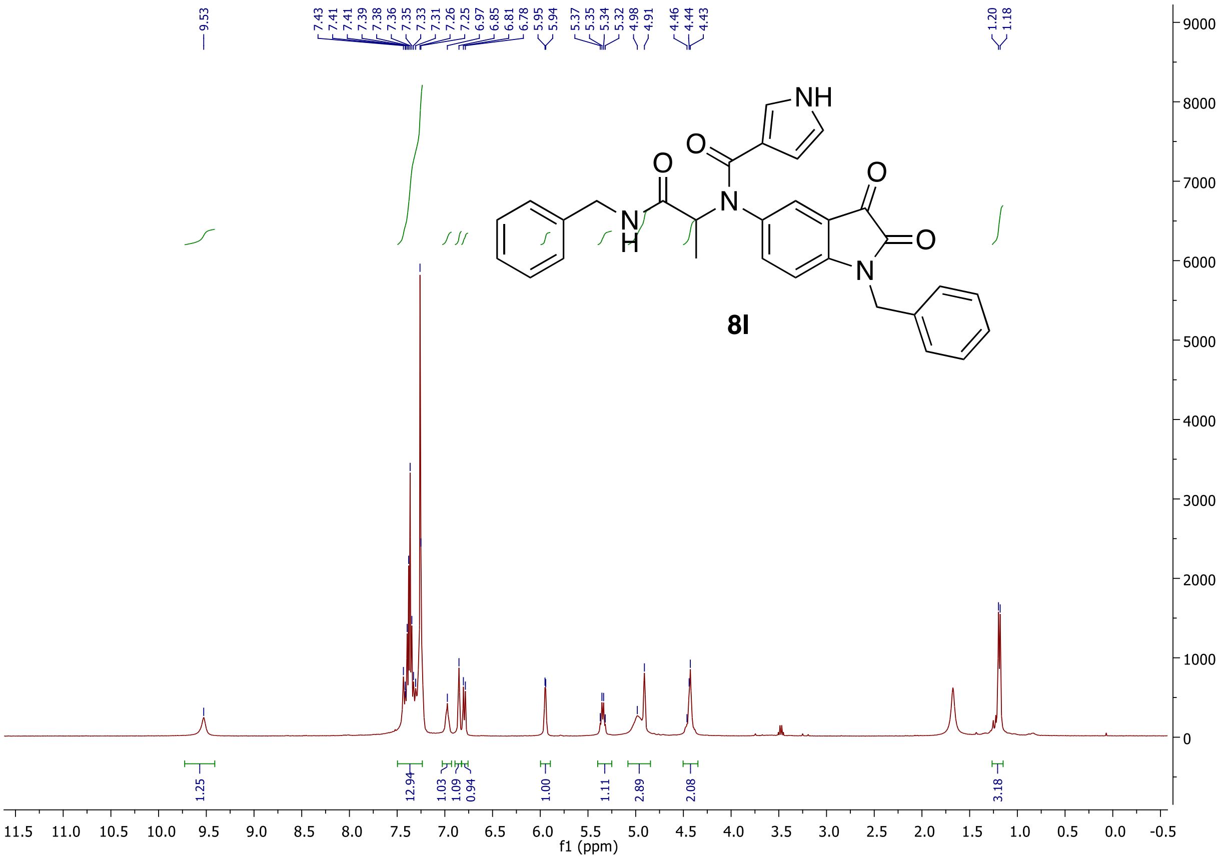
9.53
7.43 7.41 7.41 7.39 7.38 7.36 7.35 7.33 7.31 7.26 7.25 6.97 6.85 6.81 6.78 5.95 5.94
5.37 5.35 5.34 5.32 4.98 4.91 4.46 4.44 4.43
1.20 1.18
NH
O
O
O
O
N
N
H
N
8l
1.25 12.94 1.03 1.09 0.94 1.00 1.11 2.89 2.08 3.18
f1 (ppm)

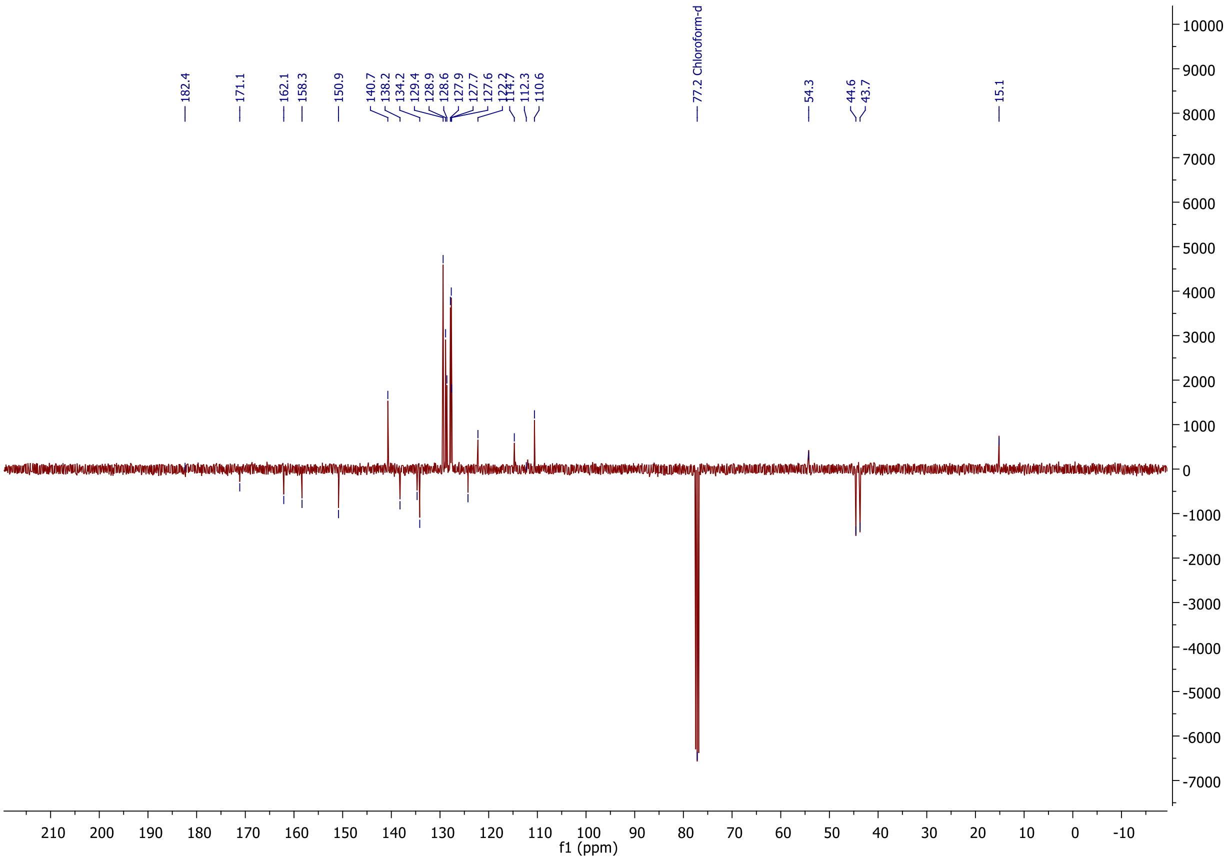
182.4 171.1 162.1 158.3 150.9 140.7 138.2 134.2 129.4 128.9 128.6 127.9 127.7 127.6 122.2 114.7 112.3 110.6
77.2 Chloroform-d
54.3 44.6 43.7 15.1
f1 (ppm)

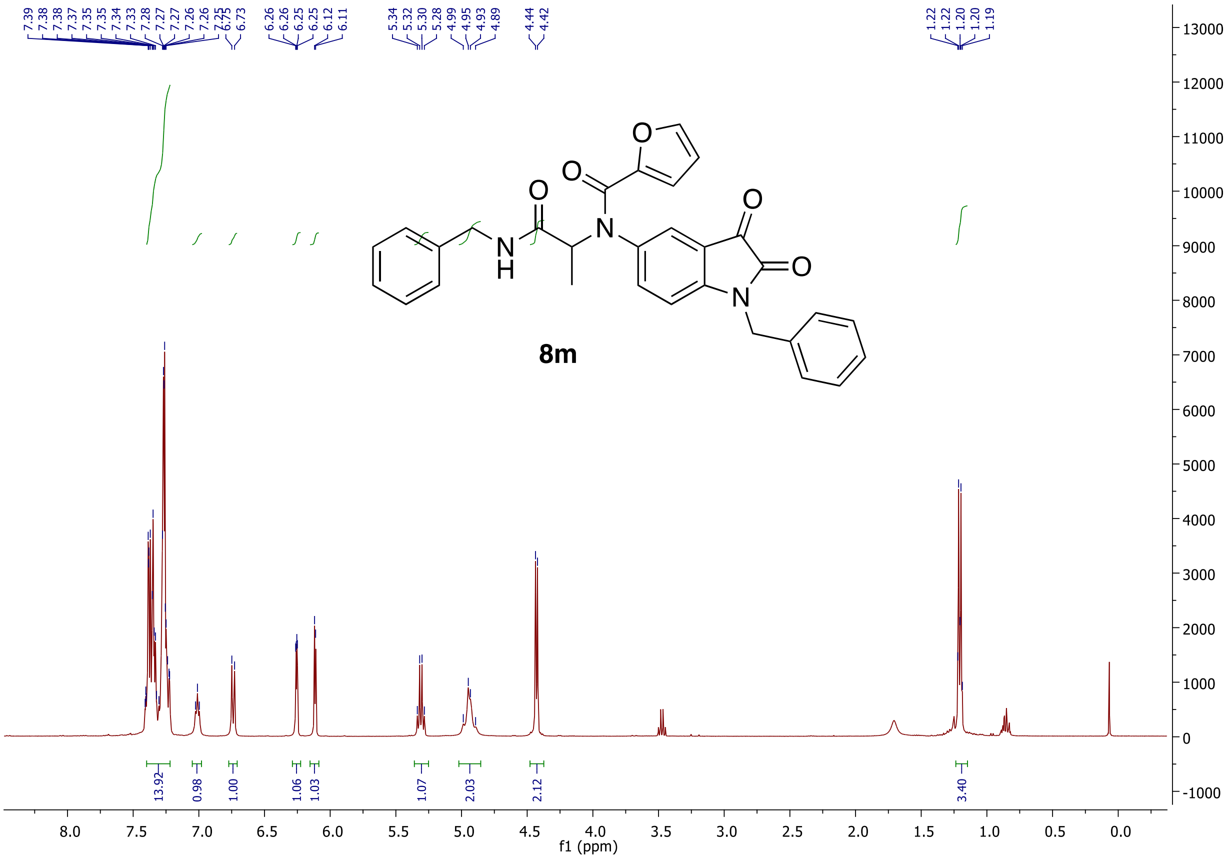
7.39 7.38 7.38 7.37 7.35 7.35 7.34 7.33 7.28 7.27 7.27 7.26 7.25 6.75 6.73
6.26 6.26 6.25 6.25 6.12 6.11
5.34 5.32 5.30 5.28 4.99 4.95 4.93 4.89
4.44 4.42
1.22 1.22 1.20 1.20 1.19
O
O
O
N
N
H
O
O
N
8m
13.92 0.98 1.00 1.06 1.03 1.07 2.03 2.12 3.40
f1 (ppm)

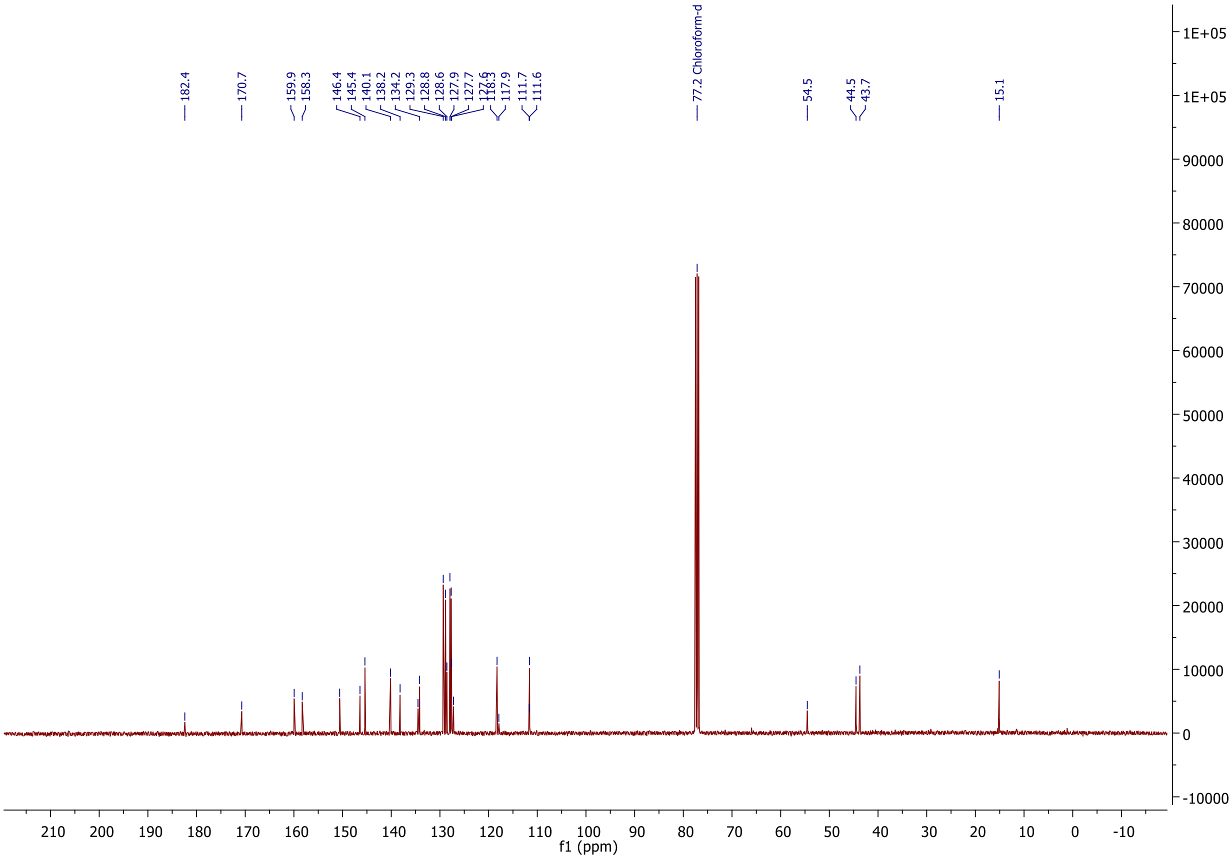
182.4
170.7
159.9 158.3
146.4 145.4 140.1 138.2 134.2 129.3 128.8 128.6 127.9 127.7 127.6 118.3 117.9 111.7 111.6
77.2 Chloroform-d
54.5
44.5 43.7
15.1
f1 (ppm)

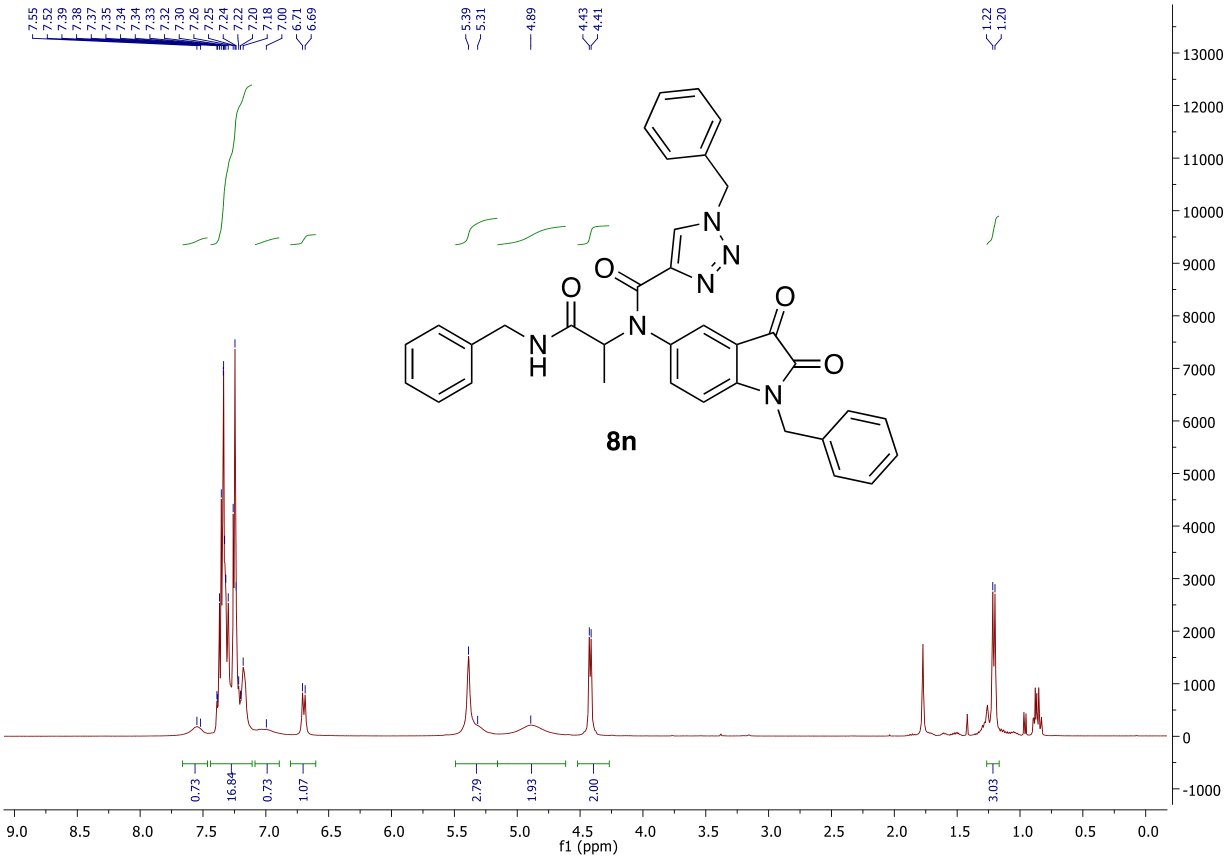
7.55
7.52
7.39
7.38
7.37
7.35
7.34
7.34
7.33
7.32
7.30
7.26
7.25
7.24
7.22
7.20
7.18
7.00
6.71
6.69
5.39
5.31
4.89
4.43
4.41
1.22
1.20
8n
0.73
16.84
0.73
1.07
2.79
1.93
2.00
3.03
f1 (ppm)

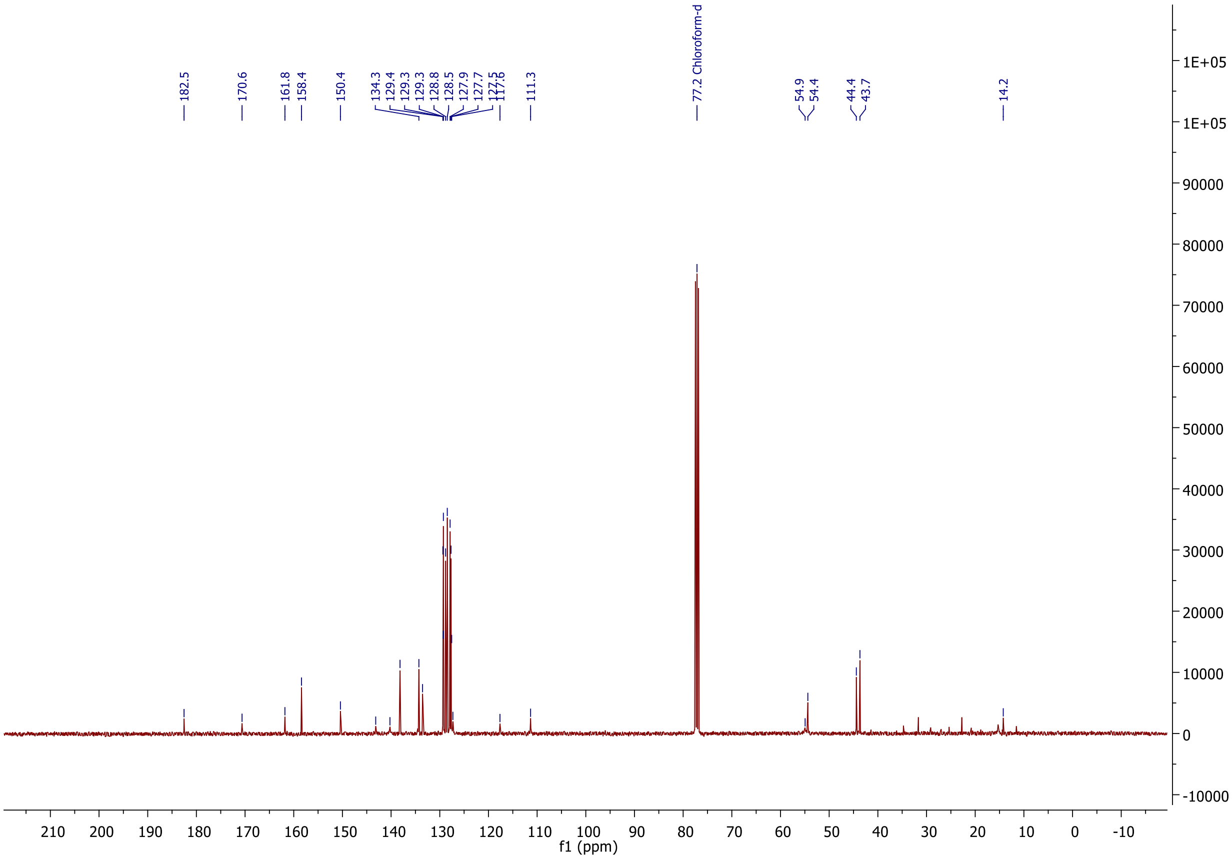
182.5
170.6
161.8
158.4
150.4
134.3
129.4
129.3
129.3
128.8
128.5
127.9
127.7
127.5
117.6
111.3
77.2 Chloroform-d
54.9
54.4
44.4
43.7
14.2
f1 (ppm)
